# Dipolar pathways in multi-spin and multi-dimensional dipolar EPR spectroscopy†

**DOI:** 10.1039/d2cp03048a

**Published:** 2022-09-08

**Authors:** Luis Fábregas-Ibáñez, Valerie Mertens, Irina Ritsch, Tona von Hagens, Stefan Stoll, Gunnar Jeschke

**Affiliations:** ETH Zurich, Laboratory of Physical Chemistry Vladimir-Prelog-Weg 2 Zurich Switzerland; Department of Chemistry, University of Washington Seattle WA 98195 Washington USA

## Abstract

Dipolar electron paramagnetic resonance (EPR) experiments, such as double electron–electron resonance (DEER), measure distributions of nanometer-scale distances between unpaired electrons, which provide valuable information for structural characterization of proteins and other macromolecular systems. We present an extension to our previously published general model based on dipolar pathways valid for multi-dimensional dipolar EPR experiments with more than two spin-1/2 labels. We examine the 4-pulse DEER and TRIER experiments in terms of dipolar pathways and show experimental results confirming the theoretical predictions. This extension to the dipolar pathways model allows the analysis of previously challenging datasets and the extraction of multivariate distance distributions.

## Abstract 2

A dipolar pathway model describing multi-spin and multi-dimensional dipolar EPR experiments and allowing for the extraction of multivariate distance distributions.

## Graphical abstract

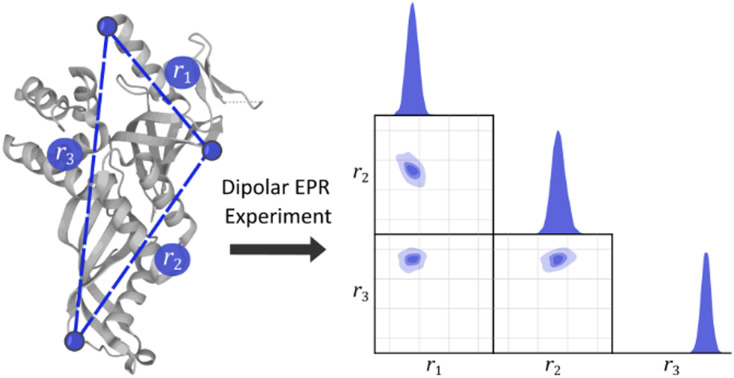

## 1 Introduction

Measuring distributions of nanometer-scale distances between unpaired electrons in naturally occurring or engineered paramagnetic systems is the primary focus of dipolar EPR spectroscopy.^1,2^ These distributions contain valuable information for the structural characterization of proteins and other macromolecular systems,^3,4^ even those lacking long-range order in a sample.^5^ For this reason, dipolar EPR spectroscopy provides unique information about structurally disordered or highly complex systems.

The most broadly applied type of dipolar EPR experiment is double electron–electron resonance (DEER, also known as pulsed electron double resonance, PELDOR). Since the introduction of the 3-pulse DEER experiment,^1^ the 4-pulse DEER experiment has become the most common,^6–8^ but many other variations have been developed.^9–20^ All these experiments use a sequence of microwave pulses to generate a spin echo and record the echo amplitude as a function of pulse timings. From the resulting modulated spin echo signal, the underlying spin–spin distance distribution can be inferred, provided one has an adequate theoretical model to describe the experimental data.

In a previous work,^21^ we introduced a uniform theoretical model of dipolar EPR spectroscopy experiments on two-spin systems based on dipolar pathways.^9,10,22–24^ In general, a dipolar signal is a linear combination of oscillatory contributions from individual dipolar pathways. While single-pathway models have found widespread application in the past, we showed that a multi-pathway model is essential to accurately describe certain experimental dipolar signals that are typically encountered in experiments such as 4-pulse and 5-pulse DEER in two-spin systems.^21^ Multi-spin systems, *i.e.* systems with more than two spins, have been studied by DEER spectroscopy in the past both in model compounds^25–27^ and protein systems.^28–35^ Multi-spin systems can be used to simultaneously infer distances between multiple pairs of spins and help elucidate geometries that would be challenging based solely on single distances obtained from two-spin systems.

In this work, we extend our previous multi-pathway model to multi-spin systems and multi-dimensional experiments, where one or more time delays are shifted separately. This considerably expands upon the previously published models for multi-spin systems measured by 4-pulse DEER.^26,27^ The new model can be used to extract multivariate distance distributions from multi-spin systems and allows the analysis of complex multi-dimensional experiments such as the triple electron resonance (TRIER) experiment,^15^ which had previously remained a challenge to model and analyze.

This paper is structured as follows. In Section 2, we present the theoretical expansion of the current dipolar pathways model to the general multi-spin case. In Section 3, we describe the 4-pulse DEER and TRIER experiments applied to multi-spin systems in terms of dipolar pathways. In Section 4, we analyze a selection of experimental 4-pulse DEER and TRIER datasets acquired on different oligoradical and protein systems to validate our theoretical model and predictions.

## 2 Theoretical model

**Fig. 1:**
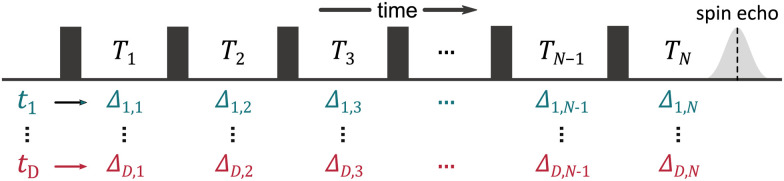
General multi-dimensional pulse sequence. The sequence consists of *N* pulses (black boxes) separated by the time intervals *T*_*n*_. The spin echo is detected after the last time interval *T*_*N*_. Several time intervals can be incremented/decremented (as indicated by the vectors *Δ*_*d*_ = (*Δ*_*d*,1_,…,*Δ*_*d*,*N*_)) according to the different experimental time axes *t*_*d*_. The number of different time axes *t*_*d*_ defines the dimensionality *D* of the experiment.

A specific dipolar EPR experiment is characterized by a pulse sequence with *N* microwave pulses and the time intervals *T*_*n*_ separating them. These timings are chosen such that a spin echo is generated at a time *T*_*N*_ after the *N*-th pulse. The index *n* = (1,2,…,*N*) indicates a time interval. To report on the coupling between electron spins within a molecular system, a modulation of the spin echo is recorded by varying one or more intervals *T*_*n*_ simultaneously or separately. Writing the sequence of delays as a vector **T** = (*T*_1_,*T*_2_,…,*T*_*N*_), the sequence timings of a *D*-dimensional experiment are given by^21,36^1
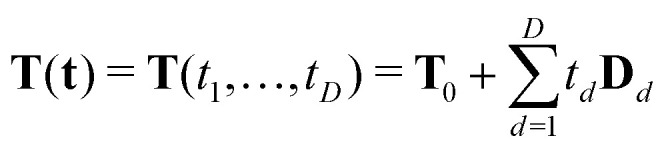
where **T**_0_ is the vector of initial delays and **t** = (*t*_1_,*t*_2_,…,*t*_*D*_) are the time coordinates that are varied during the experiment (see Fig. 1). The unitless vectors *Δ*_*d*_ specify the incrementation schemes of the corresponding time coordinates. The elements of *Δ*_*d*_ indicate whether the interval *T*_*n*_ is incremented (*Δ*_*d*,*n*_ = 1), decremented (*Δ*_*d*,*n*_ = −1) or kept constant (*Δ*_*d*,*n*_ = 0) along the *d*-th dimension. If 
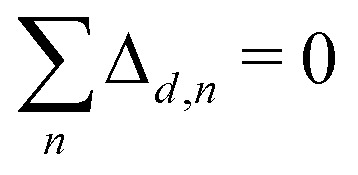
 for all *d*, then the overall length of the pulse sequence is independent of all *t*_*d*_, thus representing a constant-length experiment.

In a highly diluted sample, where the multi-spin-labeled molecules or proteins in the sample are, on average, far from each other, the spin echo amplitude *V*(**t**) is described by the following product model as originally introduced by Milov *et al.*^1^

2 *V*(**t**) = *V*_0_·*V*_intra_(**t**)·*V*_inter_(**t**)

Here, the prefactor *V*_0_ is the echo amplitude if all dipolar interactions were refocused, and *V*_intra_(**t**) and *V*_inter_(**t**) are the echo modulations from intramolecular and intermolecular spin–spin interactions, respectively (also referred to as foreground and background). The intramolecular contribution depends on the distribution of intramolecular spin–spin distances. The intermolecular contribution depends on the overall spin concentration in the sample and their spatial distribution.

In the rest of this section, we derive the expression for the modulation of the spin echo arising from an isolated multi-spin system with fixed geometry and orientation for an arbitrary multi-dimensional pulse sequence, and from there, the expressions for *V*_intra_(**t**) and *V*_inter_(**t**) for a disordered dilute sample. We limit all our theoretical treatments to *S* = 1/2 spins under weak dipolar coupling and in the high-temperature regime.^21,37^ We assume the absence of large *g*-factor anisotropies,^38,39^ exchange coupling,^40–42^ spectral diffusion,^43^ multi-spin multi-quantum coherences,^44,45^ dynamical decoupling effects, and conformer-dependent relaxation rates.^46^ If a sample or dipolar EPR experiment does not satisfy these assumptions, the model is not applicable.

### 2.1 Multi-spin dipolar echo modulation

**Fig. 2:**
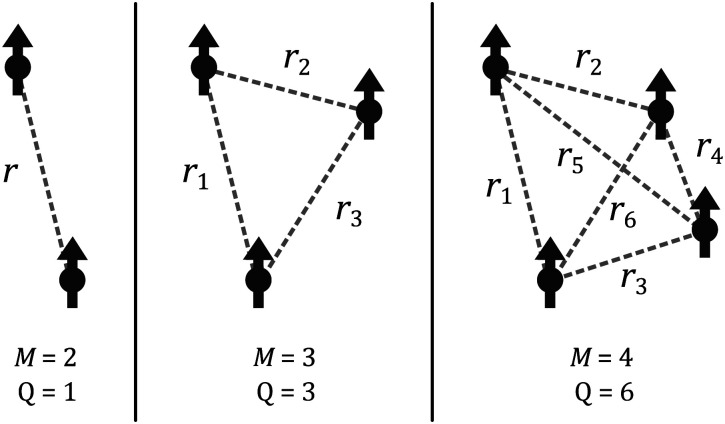
Schematic representation of dipolar interactions in different multi-spin systems. The spins are shown as black arrows and all possible interspin distances *r*_*q*_ are shown as dashed lines for a two-spin system (*M* = 2, *Q* = 1), a three-spin system (*M* = 3, *Q* = 3), and a four-spin system (*M* = 4, *Q* = 6).

We start by considering a group of *M* electron spins. This system has *Q* = *M*(*M* − 1)/2 spin pairs (see Fig. 2) with the corresponding dipolar frequencies3
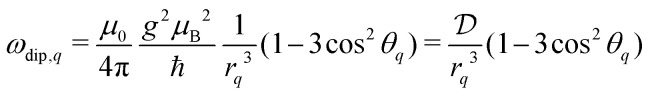
where *θ*_*q*_ is the angle between the *q*-th interspin vector and the applied magnetic field, *r*_*q*_ is the *q*-th interspin distance and the constant 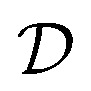 combines the *g*-values of the two electrons (close to the value of the free electron, *g*_e_), the square of the Bohr magneton *μ*_B_, the magnetic constant *μ*_0_ and the reduced Planck constant *ħ*. In addition to the dipole–dipole interaction, the spins have resonance frequency offsets *Ω*_*q*_.

For an *N*-pulse sequence, when starting at thermal equilibrium (*i.e.* any of the 2^*M*^ states are populated) there are 4^*M*(*N*+1)^ possible single-element transfer pathways, of which only those ending in single-quantum coherence after the last pulse are detectable as an FID or echo. In addition, only those lead to a stationary spin echo whose resonance offset phase is refocused at time *T*_*N*_ after the last pulse, independent of **t**.^21^ This allows us to describe the echo modulation as a function of **t** solely in terms of the dipolar phase of the individual pathways. Many of these single-element transfer pathways result in the same dipolar phase evolution for each of the dipolar interactions,^21^ and they can be collected into *K* distinct multi-spin dipolar pathways. The total spin echo amplitude *E* is a sum of the contributions *E*_*k*_ from all these multi-spin dipolar pathways4
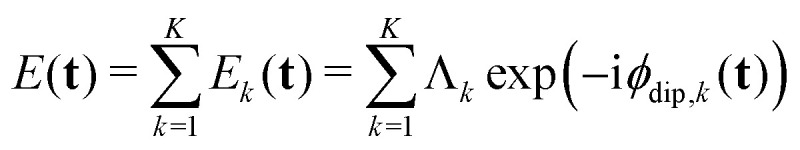
Here, *ϕ*_dip,*k*_(**t**) is the net dipolar phase accumulated along pathway *k*, *i.e.* the sum of the accumulated dipolar phases over all spin pairs *q* and all free-evolution intervals *n*,5
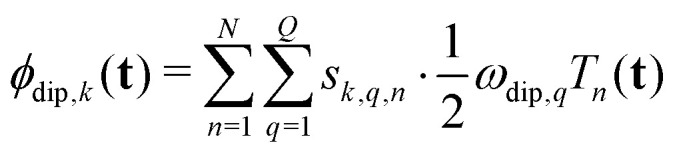
where the dipolar phase accumulation factor *s*_*k*,*q*,*n*_ indicates whether the dipolar phase due to spin pair *q* increases (+1), remains constant (0), or decreases (−1) during interval *n* for pathway *k*. The complex-valued dipolar pathway amplitude *Λ*_*k*_ is the product of the transfer amplitudes of the individual single-element transfer pathways contained in dipolar pathway *k*. Pathway amplitudes are determined by the configuration of the individual pulses such as pulse length, shape, phase and microwave power.^9,47,48^ As in our previous work, we use the scaling convention 
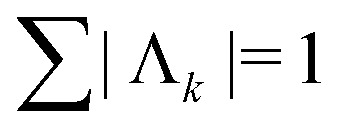
.

To separate the structure of the spin system from the details of the pulse sequence, we rewrite eqn (5) as6
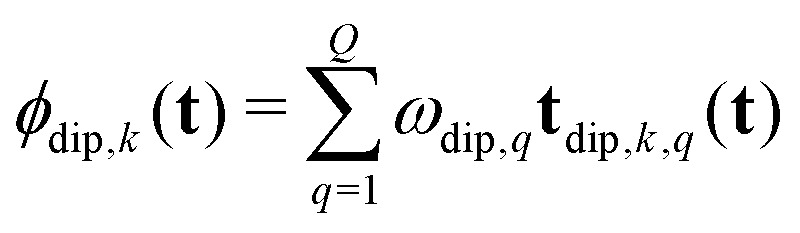
where **t**_dip,*k*,*q*_(**t**) is the *D*-dimensional vector of effective dipolar evolution times of spin pair *q* for dipolar pathway *k* given by7
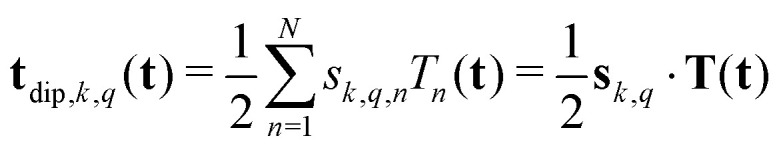


**Fig. 3:**
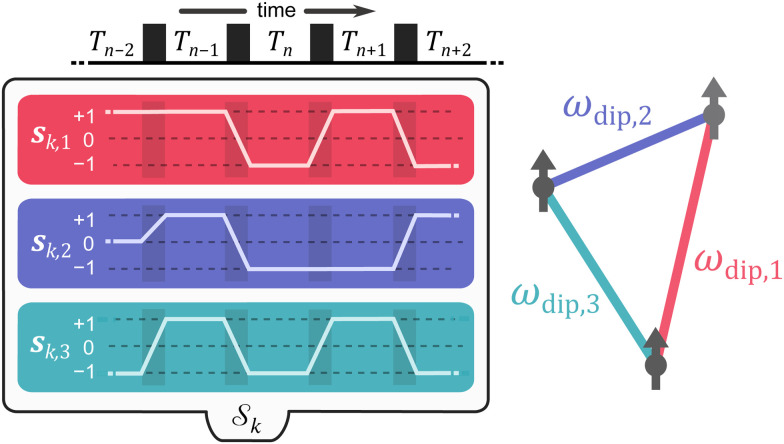
Schematic representation of a multi-spin dipolar pathway, exemplified in a three-spin system. During a sequence of pulses (shown as black boxes) and time intervals *T*_*n*_, a multi-spin dipolar pathway with multi-spin dipolar phase accumulation factors 
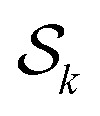
 is composed of *Q* pair dipolar phase accumulation factors **s**_*k*,*q*_ (shown as white lines) that describe whether the dipolar phase corresponding to the *q*-th dipolar interaction frequency *ω*_dip,*q*_ increases (+1), decreases (−1), or remains constant (0). Each pair dipolar pathway is colored as its corresponding dipolar interaction.

The vectors **s**_*k*,*q*_ collect the sequence of *s*_*k*,*q*,*n*_ for a given pathway and spin pair, constituting a pair dipolar pathway. This is illustrated in Fig. 3 for a three-spin system. The set of all pair dipolar accumulation factors **s**_*k*,*q*_ for a given *k* gives the multi-spin dipolar accumulation factors 
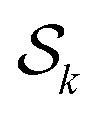
.

We now consider the modulation of a single dipolar pathway echo contribution *E*_*k*_(*t*) along an arbitrary dimension *d*. A dipolar pathway echo contribution *E*_*k*_(**t**) is modulated along the time coordinate *t*_*d*_ with respect to pair *q* only if *t*_dip,*k*,*q*,*d*_(**t**) is not constant. If *t*_dip,*k*,*q*,*d*_(**t**) is constant for all *q*, the whole pathway contribution *E*_*k*_(**t**) will be constant along dimension *d*. If the contribution is modulated, the spins along a pair dipolar pathway refocus at a time when the associated dipolar phase is zero, or, equivalently, when the effective dipolar evolution time is zero (**t**_dip,*k*,*q*_(**t**) = 0). Thus, each modulated pair dipolar pathway has a characteristic *D*-dimensional refocusing time point **t**_ref,*k*,*q*_ with elements given by8
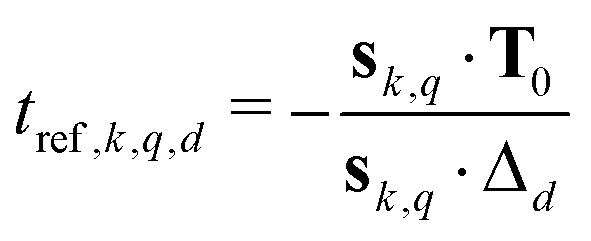
where the dipolar phase of the pathway is zero and its contribution to the total echo amplitude is at its largest.

With eqn (1) and (7), the above equation gives9
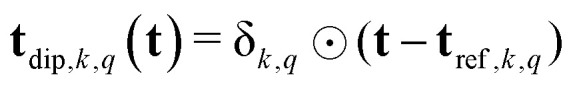
where denotes the Hadamard (element-wise) product and the *D*-dimensional prefactor vector *δ*_*k*,*q*_ has elements10
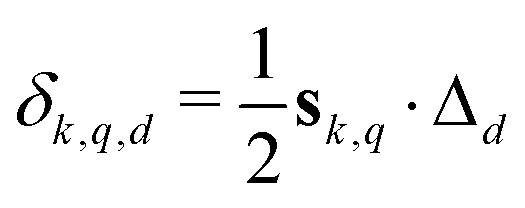
which characterize whether the contribution from spin pair *q* for pathway *k* is modulated (*δ*_*k*,*q*,*d*_ ≠ 0) or unmodulated (*δ*_*k*,*q*,*d*_ = 0) along the *d*-th time dimension. The magnitude of the prefactor also characterizes whether the pathway evolves as a harmonic (|*δ*_*k*,*q*,*d*_| > 1), subharmonic (|*δ*_*k*,*q*,*d*_| < 1), or equal (|*δ*_*k*,*q*,*d*_| = 1) of the *d*-th experimental time vector *t*_*d*_. Finally, we rewrite the total echo amplitude as11
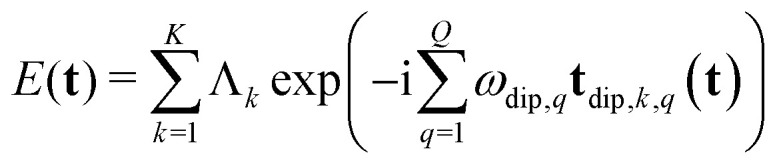
The full set of multi-spin pathways contains pairs *k* and *k*′ with opposite-sign phase accumulation factors 
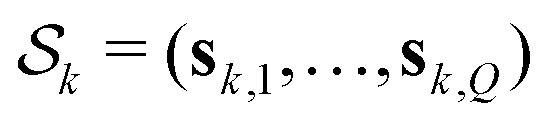
 and 
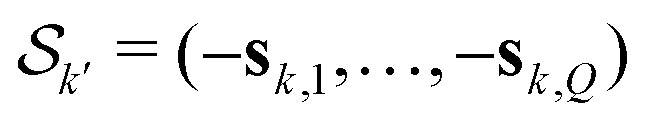
. They accumulate a dipolar phase of opposite sign 
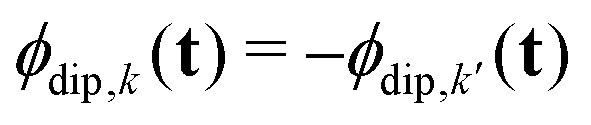
 and, in the high-temperature limit, they present the same amplitude 
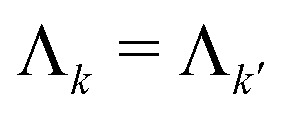
. This results in their echo contributions *E*_*k*_(**t**) and 
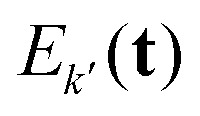
 being complex-conjugate, leading to an echo that is composed purely by real-valued contributions12
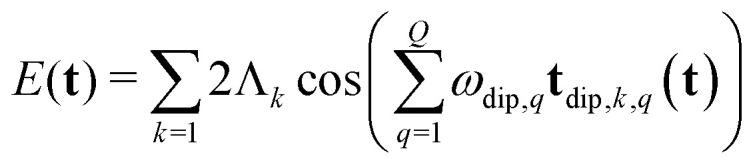
where *k* now runs over the subset of multi-spin dipolar pathways pairs. For simplicity, from now on we will refer to the combination of 
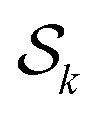
 and 
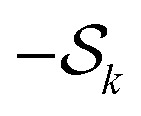
 as just 
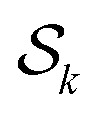
.

Now, if all spins in the system have the same spectral distribution (a common situation, *e.g.*, when all paramagnetic centers are nitroxides) then any of the spins is affected by a given pulse in the sequence with the same probability. As a consequence, all multi-spin dipolar pathways that are related by permutations of spin pairs have identical amplitude. This assumption allows us to account for fewer dipolar pathway amplitudes, considerably reducing the complexity of the model. For multi-spin systems consisting of different spin species (*e.g.*, a combination of nitroxide and trityl radicals), this approximation may not be valid and the full set of dipolar pathways must be considered separately. Likewise, if the sites have strongly different labelling efficiencies, the approximation becomes invalid.

The model derived thus far is exact within our set of assumptions, yet its complexity is cumbersome for an intuitive interpretation or for its practical application. Furthermore, the number of detectable and modulated dipolar pathways increases significantly for each additional spin in a molecule and for each pulse in an experiment.

**Fig. 4:**
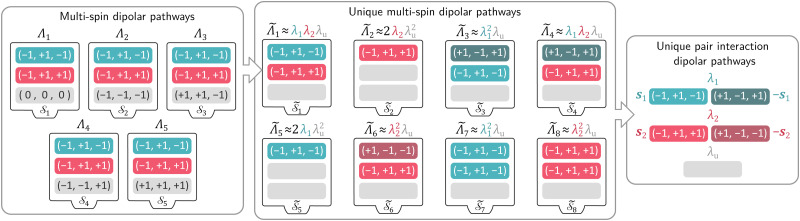
Schematic representation of the sequential simplification of the multi-spin dipolar pathways models. An example subset of five different multi-spin dipolar pathways with multi-spin accumulation factors 
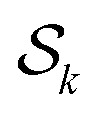
 for the one-dimensional three-pulse DEER experiment on a three-spin system is shown as boxes containing the individual modulated (|*δ*_*p*_| > 0, colored) and unmodulated (|*δ*_*p*_| = 0, grey) pair dipolar pathways **s**_*k*,*q*_, which can be collected into a unique multi-spin dipolar pathway 1 with multi-spin accumulation factors 
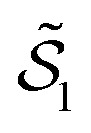
 where all unmodulated pair pathways are represented by a generic pair dipolar pathway. Other sets of multi-spin dipolar pathways can be collected into unique multi-spin dipolar pathways 2 to 8 with accumulation factors 
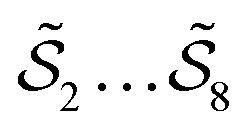
. The set of eight different unique multi-spin dipolar pathways can be described by one unmodulated pathway (grey) and two unique pair dipolar pathways with pair accumulation factors **s**_1_ (turquoise) and **s**_2_ (magenta). The amplitudes of the different pathways are given on top of each box.

To simplify the model, we re-organize the multi-spin dipolar pathways based on their modulation of the spin echo. All multi-spin pathways with the same modulated (|*δ*_*k*,*q*,*d*_| > 0) pair dipolar pathways but different unmodulated (|*δ*_*k*,*q*,*d*_| = 0) pair dipolar pathways will make the same contribution to the echo modulation. We can combine them into what we call a set of unique multi-spin dipolar pathways with multi-spin accumulation factors 
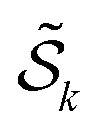
, which consist of combinations of modulated pair dipolar pathways and a single unmodulated pathway that combines all unmodulated pair dipolar pathways. The amplitudes of these unique multi-spin pathways are given by the sum of the amplitudes of the multi-spin pathways described by it. This is illustrated in Fig. 4 for a three-spin system, where five different multi-spin dipolar pathways (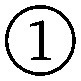 to 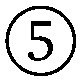) with identical modulated pair dipolar pathways and different unmodulated pair dipolar pathways can be combined into a unique multi-spin dipolar pathway 
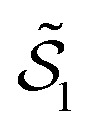
 with amplitude 
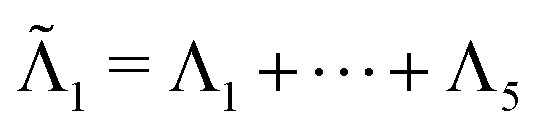
.

This description provides us with an opportunity to simplify the model even further. The set of unique multi-spin dipolar pathways can be constructed from a generic unmodulated pair dipolar pathway and a significantly reduced set of modulated pair dipolar pathways **s**_*p*_ = (**s**_*p*,1_,…,**s**_*p*,*N*_) and −**s**_*p*_ (see Fig. 4). Each of these pair dipolar pathways will have an associated refocusing time **t**_ref,*p*_ = (**t**_ref,*p*,1_,…,**t**_ref,*p*,*D*_), and factor *δ*_*p*_ = (*δ*_*p*,1_,…,*δ*_*p*,*D*_). The pair dipolar pathways in a multi-spin system for a given pulse sequence consist at least of the modulated pathways found in a two-spin system for that pulse sequence. Each unique modulated multi-spin dipolar pathway can in turn can be constructed by combination of *Q*′ < *Q* modulated pair dipolar pathways. This also significantly simplifies the amplitudes of the unique multi-spin dipolar pathways. The amplitude of unique multi-spin pathway *k* composed of *Q*′ modulated pair dipolar pathways can be well-approximately re-parameterized in terms of the amplitudes *λ*_*p*_ associated with the corresponding pair dipolar pathway and the amplitude *λ*_u_ associated with the unmodulated pair dipolar pathway13
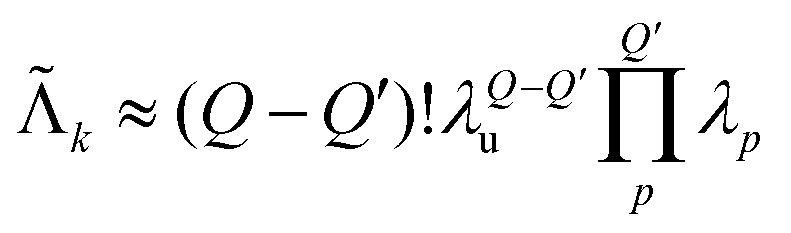
This approximation assumes the amplitude of a pair dipolar pathway to be independent of any other pairs in the system. This is again illustrated in Fig. 4 for three-pulse DEER on a three-spin system, where we can see that the accumulation factors 
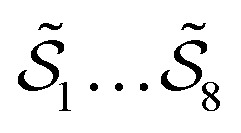
 of the unique multi-spin pathways 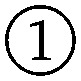 to 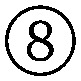 can be fully constructed from one unmodulated pair dipolar pathway and two modulated pair dipolar pathways' accumulation factors **s**_1_ and **s**_2_.

The formalism above has two important consequences. First, it allows us to describe the dipolar signals just in terms of the subset of pair dipolar pathways with accumulation factors *s*_*p*_ instead of the (significantly larger) full set of multi-spin pathways with accumulation factors 
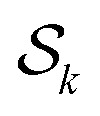
. Second, due to the normalization of the pathway amplitudes and the permutation of the multi-spin pathways it follows that all |*λ*_p_| ≤ 1/*Q*!. Therefore, the amplitude of a multi-spin pathway 
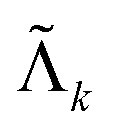
 decreases quickly with the number *Q*′ of modulated pair dipolar pathways contained in it. The presence of three or more modulated pair dipolar pathways results in negligible amplitudes. Therefore, any multi-spin system can be approximately described by just its two-spin and three-spin interactions (*i.e. Q*′ < 3) without relevant loss of accuracy. In this decisive simplification, we can thus approximate the multi-spin echo modulation as14
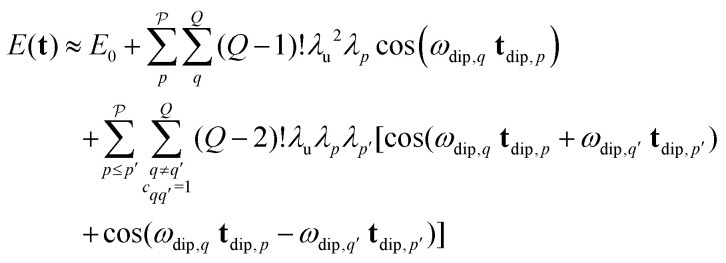
where *E*_0_ collects the constant echo amplitude contributed by all unmodulated dipolar pathways, the first term describes the two-spin contributions, and the second term the three-spin contributions to the echo modulation. The two terms of the three-spin contributions represent the different combinations of pathways with **s**_*p*_ and −**s**_*p*_, respectively, which have identical amplitudes (see Fig. 4) within our approximation. In both terms, the first sum accounts for all combinations of *Q*′ pair dipolar pathways, and the second sum accounts for the permutation symmetry of the spin system. Note that for the three-spin contributions we only need to sum over the cases where the *q*-th and *q*′-th interspin vectors are joined by a common spin 
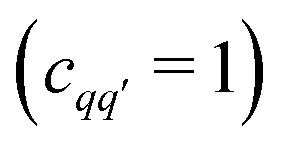
. If they are disjointed 
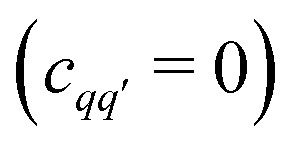
 they will not contribute to the signal. The expression above can be further simplified to15
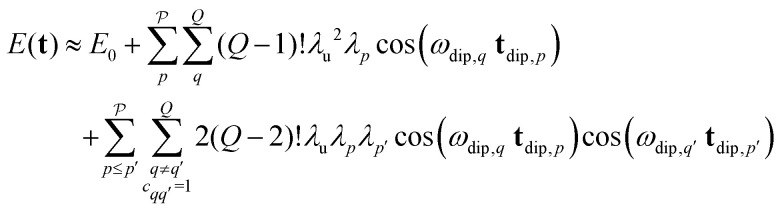
This approximate model represents a significant extension to the similar model previously published by Jeschke *et al.*^26^ Also note that this definition of the term three-spin contributions is broader than the one used by Pribitzer *et al.*,^15^ where it was used for signal contributions modulated by two dipolar frequencies along a single dimension.

### 2.2 Intramolecular contributions

To obtain an expression for the intramolecular contribution *V*_intra_(**t**) in a disordered sample, such as a frozen solution, we average the echo amplitude *E*(**t**) over the uniform orientational distribution of the spin cluster. If the molecule's conformations are distributed, we additionally average over the conformational distribution. Different choices of internal coordinates are possible to represent conformations. Here, we use the set of inter-spin distances **r** = (*r*_1_,…,*r*_*Q*_), which forms a set of non-redundant internal coordinates for *M* ≤ 4 (for larger systems, or in the presence of symmetry, other representations are more efficient). The conformational distribution is described by the *Q*-variate distance distribution *P*(**r**), which is only defined over the region of **r** that fulfills the generalized triangle inequality16
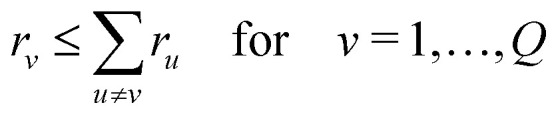
Together, the orientational and conformational averaging is17
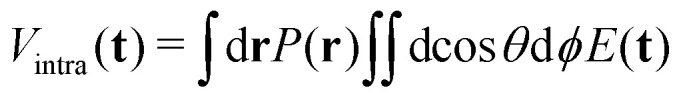
where *θ* and *ϕ* describe the orientation of the spin cluster. Inserting *E*(**t**) from eqn (15) into this gives18
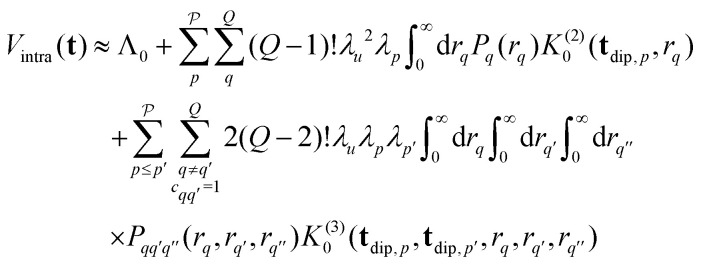
where *Λ*_0_ is the unmodulated contribution that results from integration of *E*_0_, 
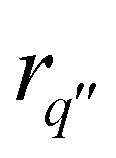
 is the distance of the interspin vector that forms a joint triangle with the *q*-th and *q*′-th interspin vectors, and *P*_*q*_(*r*_*q*_) and 
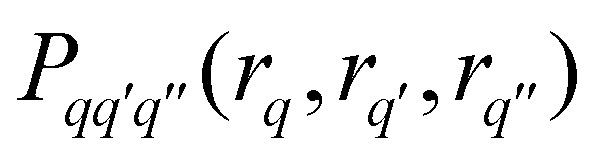
 are the univariate and trivariate marginal distance distributions19
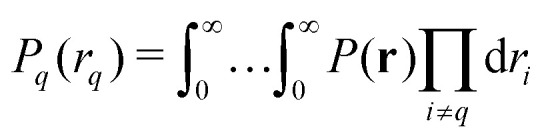
20
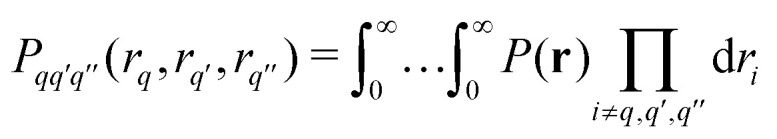
The two-spin dipolar kernel in eqn (18) is given by21
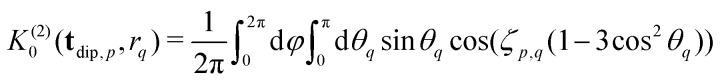
where 
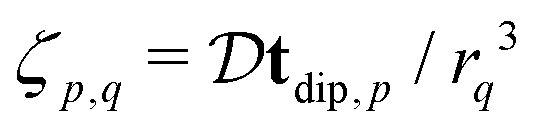
. This can be evaluated analytically^21^ to give22
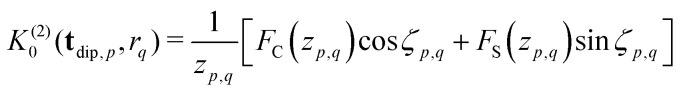
where 
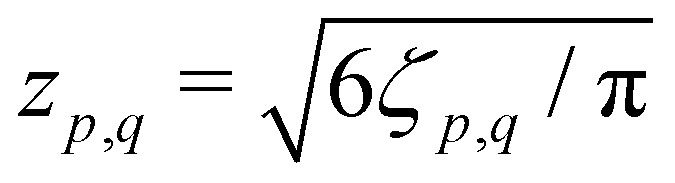
 and *F*_C_ and *F*_S_ are the cosine and sine Fresnel integral functions, respectively. The three-spin dipolar kernel in eqn (18) is given by^26,27,49^23
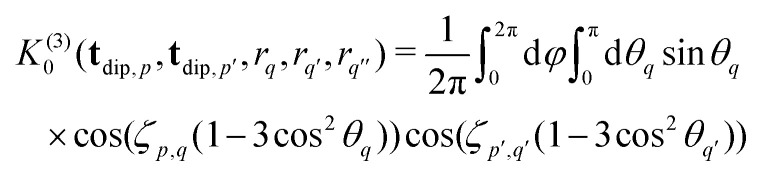
where24
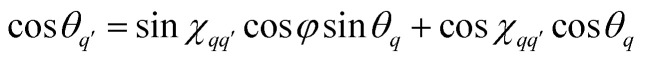
and 
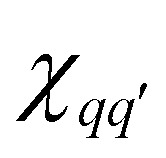
 is the angle between the interspin vectors of the *q*′-th and *q*-th spin pair, given by the law of cosines25
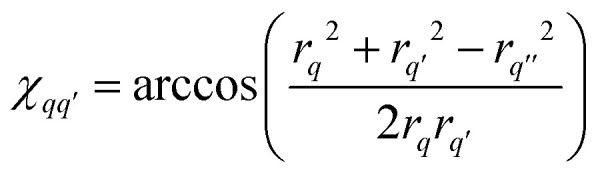
The three-spin dipolar kernel has no closed-form solution and must be evaluated numerically.

It is important to note that while the two-spin intramolecular contributions depend solely on the individual univariate marginal distance distributions, the three-spin contributions depend on the trivariate distance distributions, which encode the correlation information between triads of interspin distances. These correlations provide a new layer of valuable information for the structural characterization of the molecular systems under study.

### 2.3 Intermolecular contributions

To derive an expression for the echo modulation *V*_inter_ due to intermolecular dipolar interactions we must consider the full system with *N*_s_ spins residing in different spin clusters. We assume a random uniform distribution of spin clusters in the sample^1,50^ and disregard any volume exclusion effects.^51^ Under a high dilution of the spin clusters we can reduce the problem to pair interactions between spins and neglect any multi-spin intermolecular interactions between the spins residing in different spin clusters. The total echo arising from these interactions is the product over the individual pair contributions to the echo26
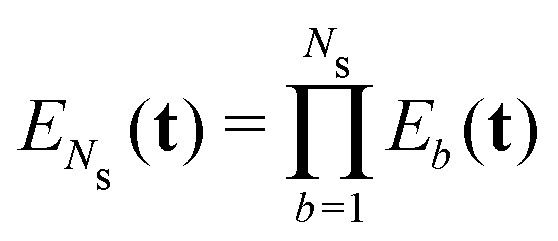
where the index *b* runs over all spins residing in other spin clusters. The total intermolecular contribution can be computed by averaging over all possible orientations and configurations of the spins in other molecules in space. The averaging situation is analogous to the two-spin case for which we already provided a detailed derivation in ref. 21. Inserting *E*(**t**) from eqn (15) into the expression above gives27
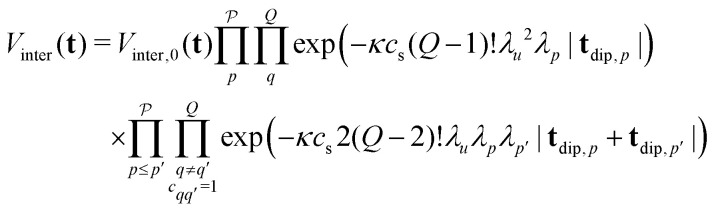
where *c*_s_ is the spin concentration, 
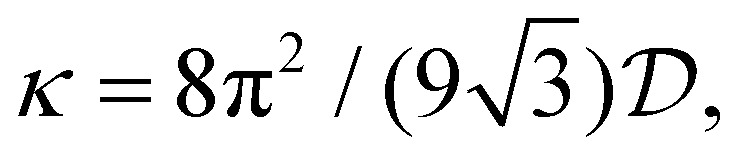
 and *V*_inter,0_ is a constant scaling factor that can be merged into *V*_0_. The intermolecular contribution is thus a product of all the exponential decays arising from each two-spin and three-spin interaction in the system. The different refocusing times of the individual dipolar pathways can result in kinks of the total intermolecular contribution, which were already observed and studied in our previous work.^21^

## 3 Specific experiments

In this section, we discuss the 4-pulse DEER and TRIER experiments in terms of their pair dipolar pathways to understand the different possible contributions to their respective dipolar signals. In the ESI,† we provide a general script to compute all possible modulated dipolar pathways contributing to the dipolar signal (within the assumptions of this work) for arbitrary pulse sequences and multi-spin systems.

### 3.1 4-Pulse DEER

**Fig. 5:**
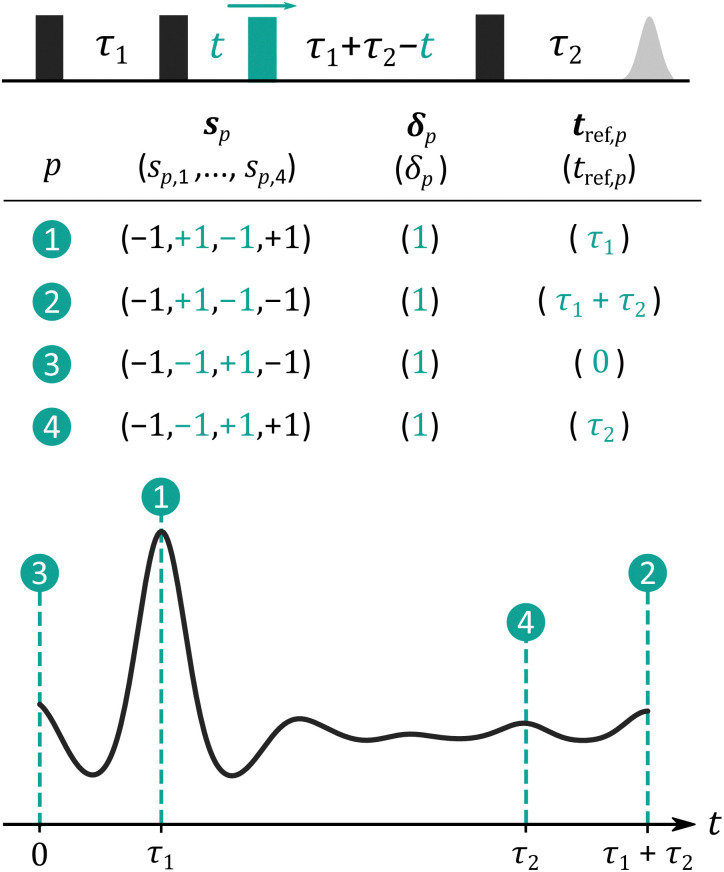
Pair dipolar pathways of the multi-spin 4-pulse DEER experiment. The 4-pulse DEER pulse sequence is shown on top, with every probe pulse represented as a black box and the pump pulse as a green box. The table contains a subset of the most commonly encountered pair dipolar pathways **s**_*p*_ along with their harmonics *δ*_*p*_ and refocusing times *t*_ref,*p*_. The pathways are ordered in decreasing estimated amplitude for commonly reported experimental conditions. The refocusing times are illustrated as dashed turquoise lines on top of a schematic 4-pulse DEER dipolar signal shown as a black line.

The 4-pulse DEER experiment is the most common dipolar EPR experiment, it is one-dimensional (**t** = *t*), and its dipolar pathways in two-spin systems have been discussed extensively.^21–23^ Dipolar signals acquired by this experiment for multi-spin systems are composed of contributions from different multi-spin pathways that can be constructed from the four unique pair dipolar pathways 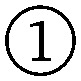 to 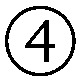 summarized in Fig. 5. The main contribution 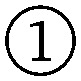 refocuses at *t* = *τ*_1_ and the contributions 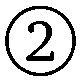 and 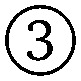 refocus at the edges of the accessible time range. 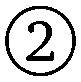 is often referred to as the “2+1” contribution.^4,48^

In the absence of any pulse excitation overlap and using π-pulses of high inversion efficiency, the 4-pulse DEER signal consists of just pathway 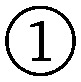. In the presence of excitation overlap, pathways 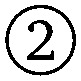 to 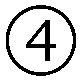 become significant.^21^

In terms of three-spin contributions, we can expect non-negligible contributions from pathways where one or two dipolar interactions evolve according to pair pathway 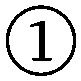. Since the 4-pulse DEER is set up such that 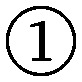 has the largest amplitude, its three-spin counterpart will also have a significant amplitude. For this same reason, we can expect very small or negligible amplitude from three-spin contributions arising from combinations of other pathways such as 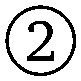 or 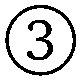. However, if their amplitude becomes significant due to considerable pulse excitation overlap or broadband or shaped pulses, their three-spin contributions can also become non-negligible. For example, the four-pulse version of the nDEER experiment,^11^ which uses such broadband pulses and records 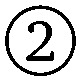 as its main contribution, would have a significant three-spin contribution from pathways where two dipolar interactions evolve according to 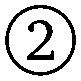 instead of 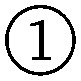.

### 3.2 TRIER

**Fig. 6:**
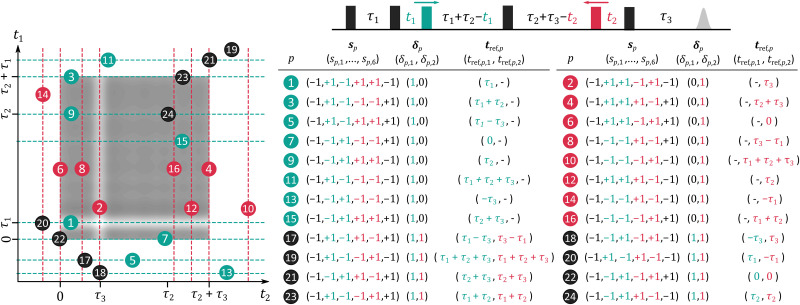
Pair dipolar pathways of the multi-spin TRIER experiment. The TRIER pulse sequence is shown on top, every probe pulse represented as a black box, the *t*_1_-shifted pump pulse as a green box, and the *t*_2_-shifted pump pulse as a red box. The table lists all possible pair dipolar pathways **s**_*p*_ along with their harmonics *δ*_*p*_ and refocusing times *t*_ref,*p*_ modulated along *t*_1_ (green), *t*_2_ (red), and both (black). The pathways are ordered in decreasing estimated amplitude for commonly reported experimental conditions. In the left panel, the refocusing times of the one-dimensional modulated contributions are illustrated as dashed green and red lines, and the two-dimensional modulated contributions as black circles on top of a schematic TRIER dipolar signal shown as greyscale contours.

The triple electron resonance (TRIER) experiment is a two-dimensional form of a six-pulse DEER experiment where the two pump pulses are shifted according to **t** = (*t*_1_,*t*_2_) (see Fig. 6).^15,52^ The experiment was originally designed for the study of frequency-domain correlations between the different dipolar frequencies in a multi-spin system. Its complexity is reflected in the large number of multi-spin pathways that can contribute to its signal. The different multi-spin pathways that can be constructed from the 24 pair pathways are summarized in Fig. 6 ordered by decreasing expected amplitude based on typical experimental conditions.

The TRIER experiment aims to record the contributions arising from the *t*_1_-modulated pathway 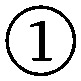 refocusing at *t*_1_ = *τ*_1_ and the *t*_2_-modulated pathway 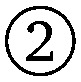 refocusing at *t*_2_ = *τ*_3_. The TRIER model proposed originally^15,52^ contained both the two-spin contributions (modulated along each time dimension) as well the three-spin contributions (modulated along both dimensions) arising from pathways 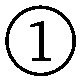 and 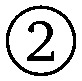. Under typical experimental conditions, both pathways will provide the largest contributions to the TRIER signal. Thus, we can expect the most intensive signal at a time **t** = (*τ*_1_, *τ*_3_).

In addition, the TRIER signal can easily present contributions from pathways where one of the probe pulses fails to invert the dipolar phase (due to insufficient inversion efficiency or pulse excitation bandwidth overlap). This happens due to imperfections of the fourth pulse in pathways 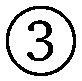 and 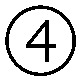, of the last pulse in pathways 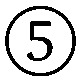 and 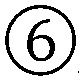, or of the second pulse in 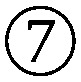 and 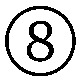. Pathways 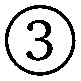 and 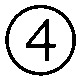 refocus at *t*_1_ = *τ*_2_ + *τ*_3_ and *t*_2_ = *τ*_1_ + *τ*_2_, respectively, at the edges of the measurable signal, analogous to the 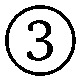 contribution in the 4-pulse DEER experiment. Pathways 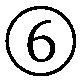 and 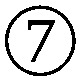 refocus at *t*_1_ = 0 and *t*_2_ = 0, respectively, at the very beginning of the experimental signal, again analogous to the 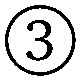 pathway of the 4-pulse DEER experiment. If the same pulse configuration is used for all probe pulses, one can expect all these additional pathways to contribute with essentially the same amplitude.

Similarly, if the inversion efficiency of the probe pulses is not perfect, pathways where two probe pulses fail to invert the dipolar phase can have non-negligible amplitudes. Pathways 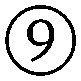, and 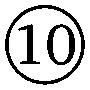 occur due to the second and fourth, pathways 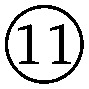 and 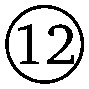 due to the fourth and last, pathways 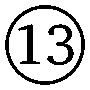 and 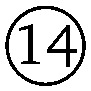 due to the second and last pulses failing to invert the dipolar phase. Similarly, pathways 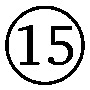 and 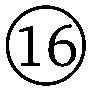 arise from the failure of all three probe pulses.

All these pathways are modulated either along *t*_1_ or *t*_2_. This is a consequence of the three-frequency nature of the experiment. The use of different frequencies for the two pump pulses results in higher amplitudes for those pathways where one pump pulse inverts the dipolar evolution and the other one does not, as in pathways 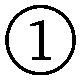 to 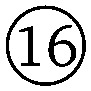. In contrast, pathways 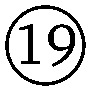 to 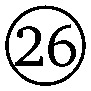 correspond to those cases where both pump pulses invert the dipolar phase evolution resulting in contributions that are modulated along both dimensions.

Another important detail is that, depending on the choice of pulse delays, the refocusing times of some dipolar pathways, *e.g.*, 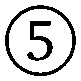, 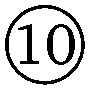 to 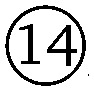, and 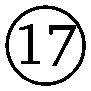 to 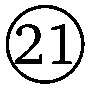 can fall outside the accessible range of *t*_1_ and *t*_2_ (from *t*_1_ = 0 to *τ*_2_ + *τ*_3_ and *t*_1_ = 0 to *τ*_1_ + *τ*_3_). It is important that their amplitudes are minimized to ensure that their contributions are negligible. Otherwise, it can pose a serious challenge to identify their presence and analyze the data.

In terms of three-spin contributions, we can expect non-negligible contributions from pathways where one or two dipolar interactions evolve according to a combination of pair pathways 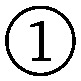 and 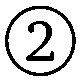. Since the TRIER experiment is set up such that 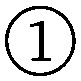 and 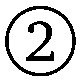 have the largest amplitudes, their one-dimensional modulated three-spin counterparts as well as the two-dimensional modulated three-spin contribution, where one dipolar interaction evolves according to 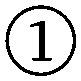 and the other along 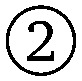, will also have a significant amplitude. For this same reason, we can expect very small or negligible amplitude from three-spin contributions arising from combinations of other pathways. However, if their amplitude becomes significant due to large pulse excitation overlap other three-spin contributions can also become non-negligible.

## 4 Experimental demonstration

To validate our theoretical model and demonstrate its utility, we analyzed a series of experiments on a variety of rigid oligo(*p*-phenyleneethynylene) (oligoPPE) tri- and tetraradicals, as well as a triply labeled protein. All datasets were analyzed using the open-source DeerLab v0.14.3 package^53^ with Python 3.8. The experimental details, as well as all experimental datasets and the corresponding analysis scripts, are provided in the ESI.†

One of the most challenging aspects of the analysis of dipolar signals arising from multi-spin systems is the proper modeling of the multivariate distance distribution *P*(**r**). In contrast to the two-spin scenario, where the distance distribution is defined over a one-dimensional distance domain, for a multi-spin system it is defined over a *Q*-dimensional distance domain. The dimensionality makes non-parametric modeling of the distribution unfeasible, requiring a parametric model for its analysis. For our analyses, based on our choice of molecular systems, we use a *Q*-variate Gaussian distribution28
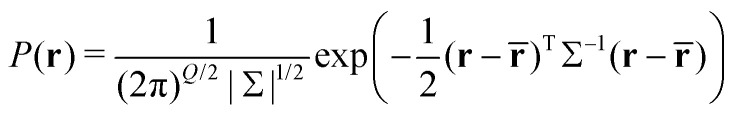
with a covariance matrix *Σ* parameterized by its Cholesky decomposition29
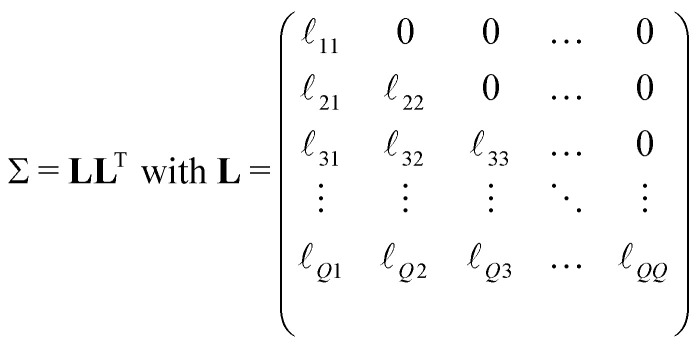
The standard deviations *σ*_*q*_ and correlation coefficients 
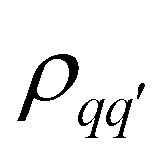
 are related to 
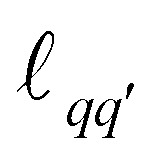
*via*30
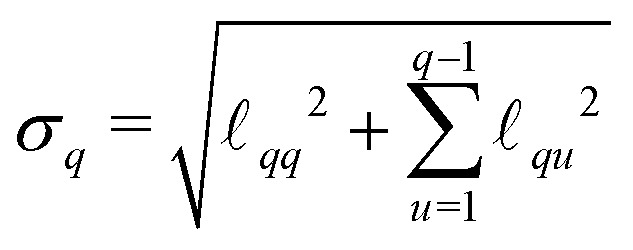
31
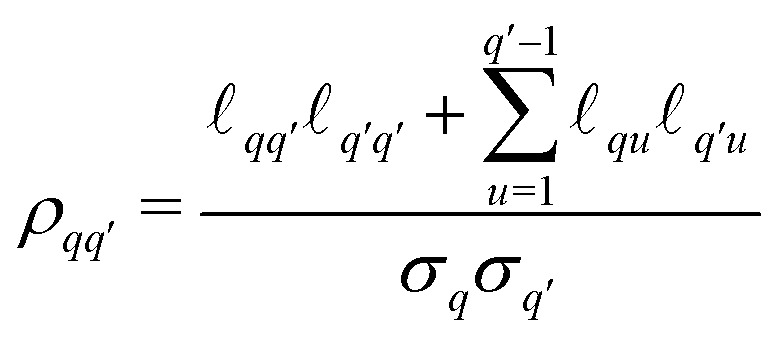
The distribution is parameterized by the mean-distance vector **r̄** = {*r̄*_1_,…,*r̄*_*Q*_}, and the set of matrix elements 
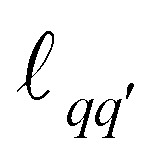
. The parametrization in terms of 
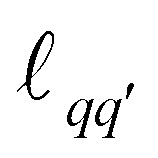
 ensures the positive definiteness of the covariance matrix, a necessary condition for *P*(**r**) to be a well-defined probability density function. Additionally, all elements of *P*(**r**) not fulfilling the generalized triangle condition in eqn (16) are set to zero. Due to this condition, the final shape of the distance distribution no longer necessarily corresponds exactly to a multivariate Gaussian distribution. Due to the large parameter space, we expect a significant risk that the optimization of the model parameters ends up in a local minimum. In order to ensure the global minimum is found, it is recommended the use of global-optimization algorithms (such as multi-start optimization^53,54^) or the use of prior information (*e.g.*, the mean inter-spin distances) to obtain a reasonable guess of the model start values. In our case, we estimated the parameter optimization start values based on information provided by structural simulations (see below). Nevertheless, we want to note that even without prior knowledge of a system's structure, the same global minimum will exist, the only difference being that the task of finding it will be computationally costlier. Another challenging aspect is the evaluation of the three-spin dipolar kernel in eqn (23). A full numerical evaluation is unfeasible, particularly for two-dimensional experiments such as TRIER. To efficiently evaluate the spherical integrals in eqn (23), we employ the SOPHE grid.^55–57^ The distance averaging in eqn (18) over the three-spin contributions was performed by repeated random sampling from the trivariate marginal distance distributions. The two-spin contributions are computed *via* the analytical solutions of the dipolar kernel in eqn (22) and *via* numerical evaluation over the univariate marginal distance distributions.

To estimate the ground truth multivariate distance distributions of the different multi-spin systems, we performed a series of structural simulations. For the oligoPPE radicals we performed simulations based on the harmonic-segmented-chains (HSC) model,^58,59^ which was tested in the past for such shape-persistent oligomers in the context of combined DEER and fluorescence resonance energy transfer (FRET) measurements.^60^ For the proteins, we simulated the multivariate distance distribution based on the protein structure using MMMx.^61^ All HSC and MMMx simulation scripts are provided in the ESI,† as well.

### 4.1 4-Pulse DEER

We first analyzed the X-band 4-pulse DEER data from two solutions of different rigid oligoPPE triradicals in dOTP measured and previously published in the dissertation of von Hagens.^62^ For each system, several 4-pulse DEER traces were recorded using varying pump pulse powers, resulting in different relative amplitudes of the two-spin and three-spin contributions. The first oligoPPE system (T111) has three nitroxide-terminated chains of equal length, each with a terminal nitroxide radical. The second oligoPPE system (T011) has two chains of equal length and one that is shorter. We fitted the datasets using the model given in eqn (18), taking into account the pair dipolar pathway 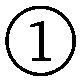 only. Due to the orientation selectivity typically encountered in such systems, the high signal-to-noise ratio of the data, and our inability to accurately model the orientation weights, we included a stretch factor in the exponential function in eqn (27) as an additional fit parameter to accommodate slight deviations from an exponential decay. All the datasets were fitted globally, with a global trivariate distance distribution and background parameters. The refocusing times of the dipolar pathways were computed from the theoretical values based on the experimental pulse delays.

**Fig. 7:**
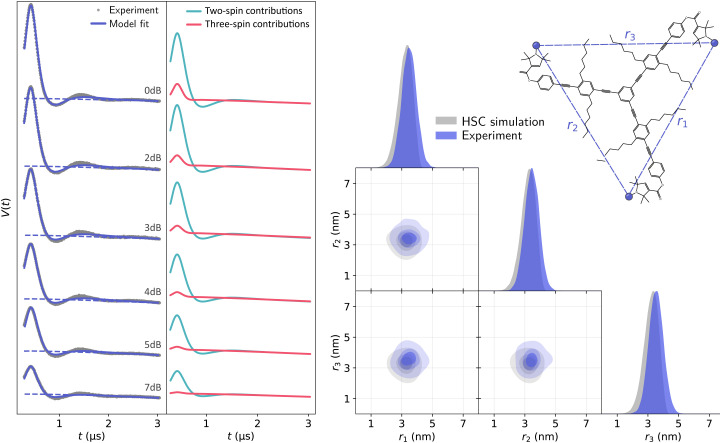
Global analysis with DeerLab of a series of X-band 4-pulse DEER of oligoPPE triradical T111 (top right) acquired with *τ*_1_ = 0.4 μs and *τ*_2_ = 6 μs, and different levels of microwave power attenuation. The experimental datasets are shown in the left panel as grey dots, along with the model fits and unmodulated contributions shown as solid and dashed blue lines, respectively. The contributions from two-spin interactions are shown as turquoise lines, and the contributions arising from three-spin interactions are shown as red lines. For clarity, only the first 3 μs out of the 6.4 μs of the recorded trace are shown (the full traces are shown in Fig. S1 in the ESI†). The right panel shows a globally fitted trivariate distance distribution. The univariate marginal distributions are shown as filled areas, and the bivariate marginal distributions are shown as colored contours. The fitted distribution is shown in blue, and the HSC simulation is shown in grey.

**Fig. 8:**
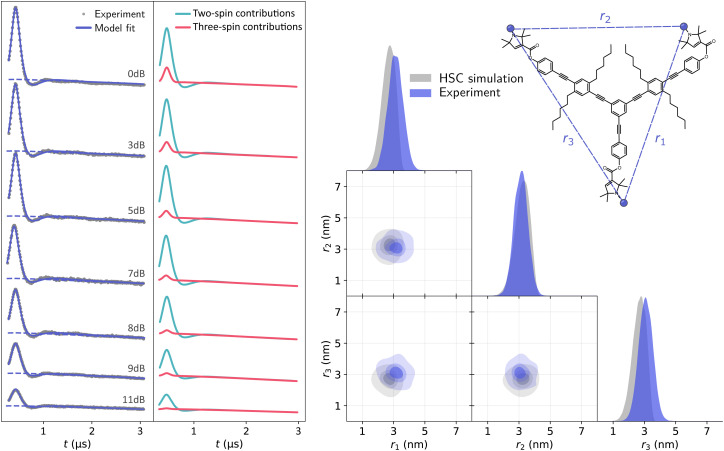
Global analysis with DeerLab of a series of X-band 4-pulse DEER of oligoPPE triradical T011 (top right) acquired with *τ*_1_ = 0.4 μs and *τ*_2_ = 12 μs, and different levels of microwave power attenuation. The experimental datasets are shown in the left panel as grey dots, along with the model fits and unmodulated contributions shown as solid and dashed blue lines, respectively. The contributions from two-spin interactions are shown as turquoise lines, and the contributions arising from three-spin interactions are shown as red lines. For clarity, only the first 3 μs out of the 12.1 μs of the recorded trace are shown (the full traces are shown in Fig. S2 in the ESI†). The right panel shows a globally fitted trivariate distance distribution. The univariate marginal distributions are shown as filled areas, and the bivariate marginal distributions are shown as colored contours. The fitted distribution is shown in blue, and the HSC simulation is shown in grey.

Global analysis results in an accurate description of all datasets by the model as seen in Fig. 7 for T111 and in Fig. 8 for T011. All experimental signals are accurately described by the global models for both the T111 and T011 datasets. Thanks to the relatively high number of high-quality datasets with varying fractions of the two-spin and three-spin contributions, the uncertainty of the results are small (see Tables S3 and S4 in the ESI†). With decreasing power (increasing attenuation) of the pump pulse power, the relative amplitude of the three-spin contributions decreases. In general, the contributions from three-spin interactions are non-negligible and significantly contribute to the overall signal. Even at lower powers (≥7 dB attenuation) the amplitude of the three-spin contributions is still non-negligible despite its considerably reduced amplitude.

The globally fitted distance distribution is in good agreement with the HSC simulations for the T111 system and in reasonable agreement for the T011 systems, with some discrepancies visible in the width of the univariate marginal distributions and a slight overestimate of the shorter distance in T011. These can be caused by minor orientation selection effects, limited accuracy of the bending parameters of the HSC model at the central ring, and the presence of a certain degree of skewness in the HSC simulated distribution not captured by a Gaussian distribution.

**Fig. 9:**
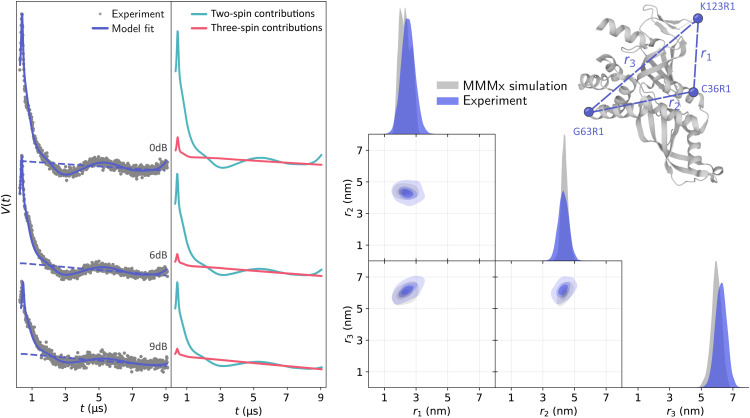
Global analysis with DeerLab of a series of Q-band 4-pulse DEER of triply MTSL-labeled Rpo47 protein (ribbon model top right, PDB : 1GO3) acquired with *τ*_1_ = 0.4 μs and *τ*_2_ = 9 μs, and different levels of microwave power attenuation. The experimental datasets are shown in the left panel as grey dots, along the model fits and unmodulated contributions shown as solid and dashed blue lines, respectively. The contributions from two-spin interactions are shown as turquoise lines, and the contributions arising from three-spin interactions are shown as red lines. The right panel shows a globally fitted trivariate distance distribution. The univariate marginal distributions are shown as filled areas, and the bivariate marginal distributions are shown as colored contours. The fitted distribution is shown in blue, and the MMMx simulation is shown in grey.

Next, we analyzed the Q-band 4-pulse DEER data from a triple MTSL-labeled RNA polymerase protein complex (Rpo47) of Rpo4 (C36R1, G63R1) and Rpo7 (K123R1) measured and previously published in the dissertation of Ritsch.^63^ Again, several 4-pulse DEER traces were recorded using varying pump pulse powers, resulting in different relative amplitudes of the two-spin and three-spin contributions. We fitted the dipolar signals using the same model as with the oligoPPE radicals. All datasets were globally fitted with a trivariate distance distribution and the three-dimensional homogeneous background in eqn (27). Again, the global analysis results in an accurate fit of all datasets by the model as shown in Fig. 9, where we can again see that the model globally fits all datasets accurately. As expected from visual inspection, the analysis confirms the non-negligible two-spin contribution from pathways 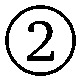 and 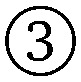 at the edges of the signal. However, the three-spin contributions related to those pathways have negligible contributions. As in the previous examples, the total three-spin contribution in all cases is non-negligible and represents a significant part of the dipolar signal, particularly at early times. The fitted trivariate distance distribution agrees reasonably well with the MMMx simulations, with some minor discrepancies between the widths of the fitted distribution and those of the simulation. These discrepancies can be attributed to the higher noise levels in the datasets and to the limitations of the multivariate Gaussian model to accurately describe the distance distribution.

**Fig. 10:**
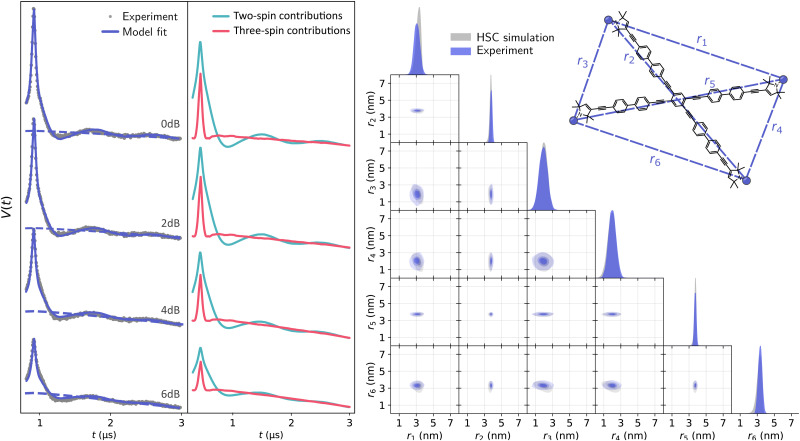
Global analysis with DeerLab of a series of X-band 4-pulse DEER of a oligoPPE tetraradical (top right), acquired with *τ*_1_ = 0.4 μs and *τ*_2_ = 8 μs, and different levels of pump pulse power. The experimental datasets are shown in the left panel as grey dots and the model fits and unmodulated contributions shown as solid and dashed blue lines, respectively. The contributions from two-spin interactions are shown as turquoise lines and the contributions arising from three-spin interactions are shown as red lines. For clarity, only the first 3 μs out of the 8.1 μs of the recorded trace are shown (the full traces are shown in Fig. S3 in the ESI†). The right panel shows the globally fitted hexavariate distance distribution. The univariate marginal distributions are shown as filled areas and the bivariate marginal distributions are shown as colored contours. The fitted distribution is shown in blue and the HSC simulation is shown in grey.

To examine a system with more than three spins, we analyzed another power series of X-band 4-pulse DEER measurements previously published by von Hagens^62^ on an oligoPPE tetraradical in dOTP consisting of four equal polymer branches first published by Schiemann *et al.*^25^ The presence of four spins requires a hexavariate distance distribution. As discussed above, we only need to account for two- and three-spin contributions and can safely neglect any four-spin contributions. We employed the same model for the dipolar signal as with the oligoPPE triradicals. Based on the rigidity of the system, to stabilize and make the analysis more computationally feasible, we assumed all off-diagonal elements of the Cholesky decomposition matrix in eqn (29) to be zero (effectively assuming correlation coefficients of zero between different distances). The global fit results are shown in Fig. 10. The global model fits all datasets accurately at all power levels. In the fit we can see that the relative amplitudes of the total three-spin contributions are significantly larger than in the three-spin cases, particularly at early times, as expected. The globally fitted hexavariate distance distribution is in astonishingly good agreement with the HSC simulation, particularly in terms of the mean distances and with minor discrepancies in the widths of the marginal distributions. These discrepancies can have the same origins as in the oligoPPE triradicals, namely the limitations of a multivariate Gaussian distribution and of the HSC model. Note that trends in distribution width, such as the narrower distributions along the two PPE backbones and broader distributions between sites in different backbones, are reproduced in the analysis of the experimental data.

In general, we can conclude that a multi-pathway model including two- and three-spin contributions provide an accurate description of 4-pulse DEER dipolar signals arising from multi-spin systems. The multivariate distance distributions inferred from the experimental data agree with the ones obtained from structural simulations and avoid the presence of artifacts caused by the use of incomplete models, such as a two-spin model (see Fig. S4–S11 in the ESI†). Note that, while our analysis has primarily profited from a global analysis over multiple datasets acquired at different pulse power levels, this kind of analysis can be formally applied even to a single 4-pulse DEER trace, but we can expect robustness to suffer substantially.

### 4.2 TRIER

To validate the TRIER pathways model, we analyzed Q-band TRIER datasets acquired using a home-built spectrometer^64^ on several three-spin systems. The detected echos were integrated and the two-dimensional signal was phase-corrected without any further signal modifications. The experiments were optimized with respect to minimal pulse excitation band overlap. At the same time, inversion efficiency with frequency-swept pulses in Q-band is known to be imperfect.^47^ Therefore, we included pair pathways 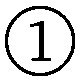 to 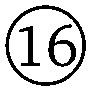 in the model, for which we expect non-negligible amplitudes due to the limited inversion efficiencies. We neglected any contributions from pathways 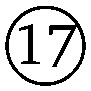 to 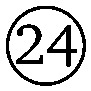 due to the well-separated three frequency bands of the different pulses. To avoid numerical issues during the data analysis we also neglect contributions from pathways 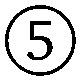, 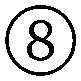, 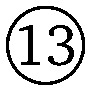, and 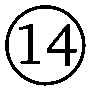 refocusing the detectable range of the signal for which we expect negligible contributions in the detected signal range. Nevertheless, the large number of parameters and complexity of the model makes the analysis of TRIER even more prone to convergence towards local minima during the fit optimization, making the use of global optimization or prior information more relevant.

**Fig. 11:**
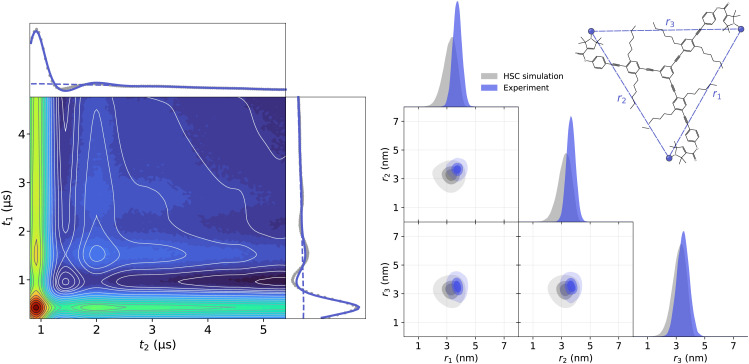
Analysis with DeerLab of the Q-band TRIER data on oligoPPE triradical T111 (top right), acquired with *τ*_1_ = 0.4 μs, *τ*_2_ = 4.6 μs, and *τ*_3_ = 0.9 μs. The two-dimensional experimental dataset is shown in the left panel as filled colored contours along with the model fit shown as greyscale contour lines. The signal integrals along each dimension are shown in the insets as grey dots for the experimental data and as a solid blue line for the model fit and a dashed blue line for the unmodulated contribution. The right panel shows the fitted trivariate distance distribution. The univariate marginal distributions are shown as filled areas and the bivariate marginal distributions are shown as colored contours. The fitted distribution is shown in blue and the HSC simulation is shown in grey for reference.

**Fig. 12:**
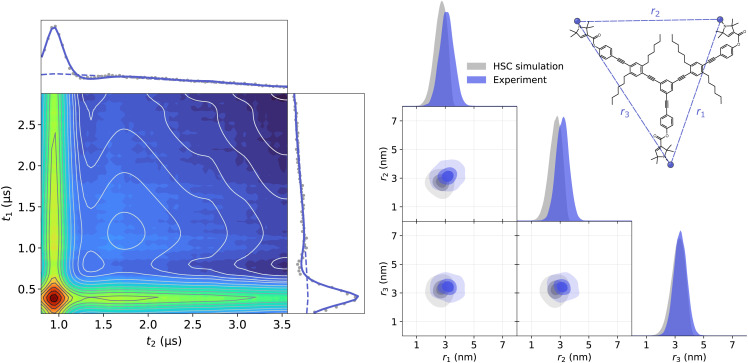
Analysis with DeerLab of the Q-band TRIER data on oligoPPE triradical T011, (top right) acquired with *τ*_1_ = 0.4 μs, *τ*_2_ = 2.8 μs, and *τ*_3_ = 0.9 μs. The two-dimensional experimental dataset is shown in the left panel as filled colored contours along the model fit shown as greyscale contour lines. The signal integrals along each dimension are shown in the insets as grey dots for the experimental data and as a solid blue line for the model fit and a dashed blue line for the unmodulated contribution. The right panel shows the fitted trivariate distance distribution. The univariate marginal distributions are shown as filled areas and the bivariate marginal distributions are shown as colored contours. The fitted distribution is shown in blue and the HSC simulation is shown in grey.

First, we acquired and analyzed new TRIER data from oligoPPEs T111 and T011. The results of the analyses are shown in Fig. 11 and 12. In the case of the T111 TRIER dataset, we can see an overall good agreement of the fitted model with the experimental data. Some discrepancies can be observed in the signal, particularly in the *t*_1_-modulated integral at around *t*_1_ ≈ 1–2 μs, which could not be resolved by considering additional pathways and are possibly due to moderate orientation selection (which has been observed in the past for such rigid polymers in 4-pulse DEER data). The fit reveals that the main contributions are from pathways 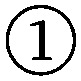 and 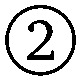, as expected, with other non-negligible minor contributions from 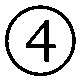, 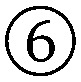, 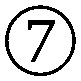, 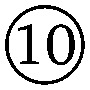, 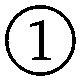, and 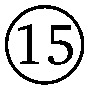. In the case of the T011 TRIER dataset, we also see a high agreement of the fitted model with the experimental data. The fitted main contributions again arise from pathways 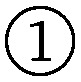 and 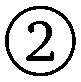, with additional non-negligible contributions from 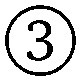, 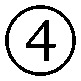, 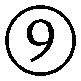, 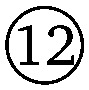, and 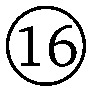.

The presence of these pathways indicates that there is a non-negligible overlap between the excitation bandwidth of the probe and pump pulses or a relatively low inversion efficiency by the probe pulses. Therefore, the presence of these additional pathways is plausible. The fitted trivariate distance distributions also show a good agreement both with the HSC simulations and with the results obtained from the analysis of the 4-pulse DEER datasets. There are again apparent differences in the fitted widths of individual distances, which could be caused by the different factors listed and discussed above.

**Fig. 13:**
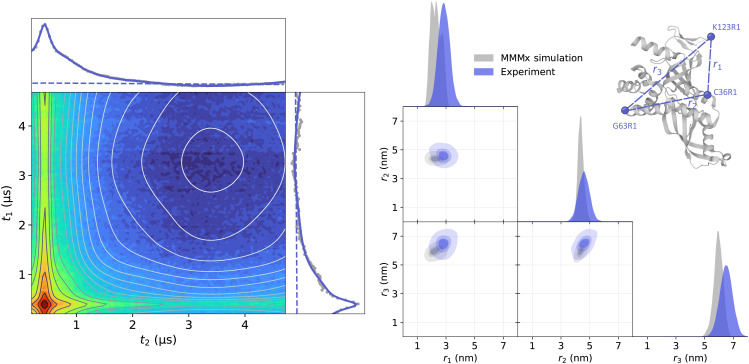
Analysis with DeerLab of the Q-band TRIER data of triply MTSL-labeled Rpo47 protein (ribbon model top right, PDB : 1GO3), acquired with *τ*_1_ = 0.4 μs, *τ*_2_ = 2.8 μs, and *τ*_3_ = 0.4 μs. The two-dimensional experimental dataset is shown in the left panel as filled colored contours along the model fit shown as greyscale contour lines. The signal integrals along each dimension are shown in the insets as grey dots for the experimental data and as a solid blue line for the model fit and a dashed blue line for the unmodulated contribution. The right panel shows the fitted trivariate distance distribution. The univariate marginal distributions are shown as filled areas and the bivariate marginal distributions are shown as colored contours. The fitted distribution is shown in blue and the MMMx simulation is shown in grey.

Next, we analyzed the TRIER data from the triply labeled Rpo47 protein complex previously published by Pribitzer *et al.*^52^ The dataset presents additional noise along the *t*_1_-dimension due to technical issues with the hardware of the home-built spectrometer at the time when this data was acquired. We employed the same TRIER model as with the oligoPPE radicals, including pathways 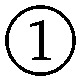 to 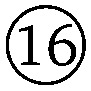 and a three-dimensional homogeneous background model. The results of the analysis are shown in Fig. 13. Despite the increased noise level, the fit shows a good agreement with the experimental data. The fitted distance distribution also agrees reasonably with an MMMx simulation as well as with the results obtained from the 4-pulse DEER experiments.

In general, we see that, despite the high complexity of the TRIER dipolar signal, the multi-pathway model provides accurate descriptions of the experimental data and reasonable estimates of the multivariate distance distributions. We can also see good agreement between the results obtained from a series of 4-pulse DEER experiments with variable pump pulse flip angle and TRIER experiments. In contrast to 4-pulse DEER, the two-dimensional nature of the TRIER signal provides enough information to robustly fit the model without the need for a global-analysis approach. While TRIER data have been analyzed in the frequency domain,^15^ the multi-pathway model provides the first reliable approach for analyzing TRIER data directly in the distance domain. Our previous work^52^ relied on an incomplete model of the dipolar signal as well as on a data analysis method, which we now consider to be inappropriate. This method was based on a simplistic background correction, which fails to account for important background features,^65^ the application of a smoothing filter that can enhance correlations or possibly even introduce spurious ones, and the use of a two-dimensional approximate Pake transformation based on the two-spin dipolar kernel, which is inaccurate for the multi-spin case.

## 5 Conclusions

Multi-spin systems have long been an attractive target of dipolar EPR spectroscopy studies. We have presented a comprehensive model for describing multi-dimensional dipolar EPR experiments on multi-spin systems within the set of assumptions listed in this work. This model expands our previous model for two-spin systems based on dipolar pathways. Under currently achievable experimental conditions, a dipolar signal for any multi-spin system can be described by its contributions arising from two- and three-spin interactions, which in turn can be well approximated in terms of pair dipolar pathways. We have illustrated this by examining the commonly employed one-dimensional 4-pulse DEER experiment and the more recent two-dimensional TRIER experiment. The presence of more than two spins can significantly increase the complexity of the models compared to the two-spin case. However, when applying these models to experimental multi-spin data, a large majority of dipolar pathways can be reasonably neglected under typical experimental conditions. The contributions from many pathways can be minimized experimentally by minimizing the excitation band overlap and maximizing the inversion efficiency of the pulses. Only if the contribution of a dipolar pathway exceeds the noise level must it be included in the analysis of the experimental data. Furthermore, unless the overall modulation depth of a dipolar signal is extremely low, the three-spin contributions cannot be safely neglected, and the analysis of the dipolar signal in terms of just two-spin interactions is inaccurate. We have tested the multi-pathway model by analyzing different series of 4-pulse DEER and TRIER datasets acquired on different three- and four-spin model compounds as well as on a triple-labeled protein complex. The model, with a proper selection of dipolar pathways, is able to accurately describe the experimental data.

The model-based analysis of experimental multi-spin dipolar data allows the extraction of multivariate distance distributions. We have also shown that the extraction of such multivariate distance distributions and the correlation information between the different distances are not limited to three-frequency experiments such as TRIER but can as well rely on a series of two-frequency four-pulse DEER experiments with varying pump pulse flip angles. The fitted multivariate distance distributions are expected to provide precious information, *e.g.*, for the characterization of conformation ensembles of partially disordered protein systems. Since non-parametric approaches are currently unfeasible, the most challenging aspect is the choice of model for the multivariate distance distribution and the size of the parameter space associated with that model.

The multi-pathway model further provides the means to analyze complex experiments, such as the three-frequency TRIER experiment, for which no complete model had been established before to accurately describe the data and analyze them in terms of a complete multivariate distance distribution. While the TRIER experiment is theoretically and experimentally complex, the two-dimensional information provided by its signals represents an important means of studying multi-spin systems.

Future work is needed to address the experimental and numerical conditions under which the model information can be best extracted from multi-spin dipolar signals. Particularly, additional efforts will be required to improve the numerical fitting protocols and to ensure the uniqueness and robustness of the analysis. The study of other types of multivariate distance distribution models will also be required for the extended applicability of model-based analysis of multi-spin dipolar EPR spectroscopy experiments.

## Author contributions

L. F. I., S. S. and G. J. developed the theory. V. M. performed the oligoPPE TRIER measurements, T. v. H. performed the oligoPPE DEER measurements, I. R. performed the Rpo47 DEER and TRIER measurements. L. F. I implemented and performed the analysis of all experimental datasets. L. F. I., V. M., S. S. and G. J. contributed towards writing the manuscript.

## Conflicts of interest

There are no conflicts to declare.

## Supplementary Material

CP-024-D2CP03048A-s001

CP-024-D2CP03048A-s002

CP-024-D2CP03048A-s003

CP-024-D2CP03048A-s004

CP-024-D2CP03048A-s005

CP-024-D2CP03048A-s006

CP-024-D2CP03048A-s007

CP-024-D2CP03048A-s008

CP-024-D2CP03048A-s009

CP-024-D2CP03048A-s010

CP-024-D2CP03048A-s011

CP-024-D2CP03048A-s012

CP-024-D2CP03048A-s013

CP-024-D2CP03048A-s014

CP-024-D2CP03048A-s015

CP-024-D2CP03048A-s016

CP-024-D2CP03048A-s017

CP-024-D2CP03048A-s018

CP-024-D2CP03048A-s019

CP-024-D2CP03048A-s020

CP-024-D2CP03048A-s021

CP-024-D2CP03048A-s022

CP-024-D2CP03048A-s023

CP-024-D2CP03048A-s024

CP-024-D2CP03048A-s025

CP-024-D2CP03048A-s026

CP-024-D2CP03048A-s027

CP-024-D2CP03048A-s028

CP-024-D2CP03048A-s029

CP-024-D2CP03048A-s030

CP-024-D2CP03048A-s031

CP-024-D2CP03048A-s032

CP-024-D2CP03048A-s033

CP-024-D2CP03048A-s034

CP-024-D2CP03048A-s035

CP-024-D2CP03048A-s036

CP-024-D2CP03048A-s037

CP-024-D2CP03048A-s038

CP-024-D2CP03048A-s039

CP-024-D2CP03048A-s040

CP-024-D2CP03048A-s041

CP-024-D2CP03048A-s042

CP-024-D2CP03048A-s043

CP-024-D2CP03048A-s044

CP-024-D2CP03048A-s045

CP-024-D2CP03048A-s046

CP-024-D2CP03048A-s047

CP-024-D2CP03048A-s048

CP-024-D2CP03048A-s049

CP-024-D2CP03048A-s050

CP-024-D2CP03048A-s051

CP-024-D2CP03048A-s052

CP-024-D2CP03048A-s053

CP-024-D2CP03048A-s054

CP-024-D2CP03048A-s055

CP-024-D2CP03048A-s056

CP-024-D2CP03048A-s057

CP-024-D2CP03048A-s058

CP-024-D2CP03048A-s059

CP-024-D2CP03048A-s060

CP-024-D2CP03048A-s061

CP-024-D2CP03048A-s062

CP-024-D2CP03048A-s063
