# Supplementary material for: Dipolar pathways in multi-spin and multi-dimensional dipolar EPR spectroscopy
Source: Phys Chem Chem Phys. 2022 Sep 8;24(37):22645–60. doi: 10.1039/d2cp03048a (PMC9516884; doi:10.1039/d2cp03048a)
Supplement: CP-024-D2CP03048A-s001 [file CP-024-D2CP03048A-s001.html]

analysis\_multispin\_DEER


In [ ]:

```
import warnings
import numpy as  np 
import deerlab as dl 
from multispin_analysis_module import *
from scipy.interpolate import interp1d
from scipy.special import factorial
```

## Model (Three spins)¶

In [ ]:

```
#------------------------------------------------------------------------------
def threespin_dipolarsignal(t,means,cholfactors,tau1,tau2,lamu,lam1,lam23,conc_decay,d,threespin=False,fractal=False):

    trefs,δs,λs= [],[],[]
    Npermutations = int(factorial(len(means)))

    ### Two-spin contributions
    tref = [ tau1, None, None]
    δ    = [    1,    0,    0]
    trefs = add_permutations(trefs,tref)
    δs = add_permutations(δs,δ)
    λs += [lam1*lamu*lamu]*Npermutations

    if lam23>0:
        tref = [ tau1+tau2, None, None]
        δ    = [         1,    0,    0]
        trefs = add_permutations(trefs,tref)
        δs = add_permutations(δs,δ)
        λs += [lam23*lamu*lamu]*Npermutations

        tref = [ 0, None, None]
        δ    = [ 1,    0,    0]
        trefs = add_permutations(trefs,tref)
        δs = add_permutations(δs,δ)
        λs += [lam23*lamu*lamu]*Npermutations

    ### Three-spin contributions
    if threespin:
        tref = [ tau1, tau1, None]
        δ    = [    1,    1,    0]
        trefs = add_permutations(trefs,tref)
        δs = add_permutations(δs,δ)
        λs += [2*lam1*lam1*lamu]*Npermutations

        lam = 2*lam23*lam23*lamu
        if lam>0.001:
            tref = [ tau1+tau2, tau1+tau2, None]
            δ    = [         1,         1, 0]
            trefs = add_permutations(trefs,tref)
            δs = add_permutations(δs,δ)
            λs += [lam]*Npermutations
        
            tref = [ 0,    0, None]
            δ    = [ 1,    1,    0]
            trefs = add_permutations(trefs,tref)
            δs = add_permutations(δs,δ)
            λs += [lam]*Npermutations

    # Monte-Carlo multivariate distance integration
    Nsamples = 500000
    np.random.seed(seed=1)
    rsamples = Pmultivar(means,cholfactors).rvs(Nsamples)
    rsamples = np.maximum(rsamples,1e-16) # Avoid values exactly at zero 

    # Triangle inequalities
    Ndistances = np.shape(rsamples)[1]
    idx = np.arange(Ndistances)
    triangle_condition = np.full_like(rsamples,False)
    for n in range(Ndistances):
        idx = np.roll(idx,-1,axis=0)
        triangle_condition[:,n] = np.sum(rsamples[:,idx[:-1]],axis=1) > rsamples[:,idx[-1]]
    triangle_condition = np.all(triangle_condition,axis=1)
    # Discard samples that do not satisfy triangle inequalities
    rsamples = rsamples[triangle_condition,:]

    # Construct the dipolar signal 
    λ0 = np.maximum(1 - np.sum(λs),0)
    Vintra,Vinter = λ0,1
    twospin_contributions,threespin_contributions = [],[]
    # Loop over all dipolar pathways
    for δ,tref,λ in zip(δs,trefs,λs):

        # Set trefs defined as None to an arbitrary numerical value
        tref = [tref[n] if δn!=0 else 0 for n,δn in enumerate(δ)]

        # Number of spins participating in the pathway
        Nspin = np.sum(abs(δ))+1 

        if Nspin==2:
            n = int(np.where(δ==1)[0])

            # Estimate the 1D-marginal distance distribution
            Pmarginal,bins = np.histogram(rsamples[:,n], bins=300)
            rgrid = (bins[:-1] + bins[1:])/2
            Pmarginal = Pmarginal/np.trapz(Pmarginal,rgrid)

            # Two-spin intramolecular contribution
            Vintra_contr = dl.dipolarkernel(δ[n]*(t-tref[n]),rgrid)@Pmarginal
            twospin_contributions.append(λ*Vintra_contr)

        if Nspin==3:
            # Non-uniformly subsample the time-domain (speedup)
            n = int(np.where(δ==1)[0][0])
            np.random.seed(0)
            subsampling = np.exp(-6*abs(t-tref[n])/max(t))*np.random.rand(len(t))>0.05
            subsampling[-1] = 1
            tsub = t[subsampling]
            # Average three-spin contribution over all distances
            Nsamples = 150
            Vintra_contr = 0
            for rs in rsamples[:Nsamples,:]: 
                r1,r2,r3 = rs
                # Three-spin intramolecular contribution
                Vintra_contr += threespin_dipolarkernel(tsub,r1,r2,r3,δ,tref) 
            Vintra_contr /= Nsamples
            Vintra_contr = interp1d(tsub,Vintra_contr, fill_value='extrapolate')(t)

            threespin_contributions.append(λ*Vintra_contr)

        Vintra += λ*Vintra_contr

        # Intermolecular contribution
        Vinter *= Bbasis(t,λ,conc_decay,d,δ,tref,fractal=fractal) 

    Vinter /= np.max(Vinter)
    V = Vintra*Vinter

    return V,λ0,Vinter,Vintra,twospin_contributions,threespin_contributions
#------------------------------------------------------------------------------

#------------------------------------------------------------------------------
def construct_threespin_dipolarmodel(t,tau1_exp,tau2_exp,threespin=False,fractal=False):

    def _threespin_dipolarsignal(mean1,mean2,mean3,chol11,chol22,chol33,chol21,chol31,chol32,tau1,tau2,lamu,lam1,lam23,conc_decay,d):
            Vfit = threespin_dipolarsignal(t,[mean1,mean2,mean3],[chol11,chol22,chol33,chol21,chol31,chol32],tau1,tau2,lamu,lam1,lam23,conc_decay,d,threespin=threespin, fractal=fractal)[0]
            return Vfit 
    # Construct model 
    Vmultispin = dl.Model(_threespin_dipolarsignal)
    Vmultispin.description = 'Three-spin dipolar signal arising from a 4-pulse DEER experiment'
    # Set parameter properties
    Vmultispin.addlinear('scale',lb=0, description='Echo amplitude in the abscense of dipolar modulations')
    Vmultispin.mean1.set(lb=1.0, ub=8.0, par0=3.0, unit='nm', description='Average inter-spin distance  #1')
    Vmultispin.mean2.set(lb=1.0, ub=8.0, par0=3.0, unit='nm', description='Average inter-spin distance  #2')
    Vmultispin.mean3.set(lb=1.0, ub=8.0, par0=3.0, unit='nm', description='Average inter-spin distance  #3')
    Vmultispin.chol11.set(lb=0.0, ub=0.8, par0=0.40, unit='nm', description='Cholesky factor ℓ11' )
    Vmultispin.chol22.set(lb=0.0, ub=0.8, par0=0.40, unit='nm', description='Cholesky factor ℓ22' )
    Vmultispin.chol33.set(lb=0.0, ub=0.8, par0=0.40, unit='nm', description='Cholesky factor ℓ33' )
    Vmultispin.chol21.set(lb=-1.0, ub=1.0, par0=0.0, unit='nm', description='Cholesky factor ℓ21' )
    Vmultispin.chol31.set(lb=-1.0, ub=1.0, par0=0.0, unit='nm', description='Cholesky factor ℓ31' )
    Vmultispin.chol32.set(lb=-1.0, ub=1.0, par0=0.0, unit='nm', description='Cholesky factor ℓ32' )
    Vmultispin.tau1.set(lb=tau1_exp-0.10, ub=tau1_exp+0.10, par0=tau1_exp, unit='μs', description='First inter-pulse delay' )
    Vmultispin.tau2.set(lb=tau2_exp-0.10, ub=tau2_exp+0.10, par0=tau2_exp, unit='μs', description='Second inter-pulse delay' )
    Vmultispin.lamu.set(lb=0.0, ub=2.00, par0=1, unit=None, description='Amplitude of unmodulated pairwise pathway' )
    Vmultispin.lam1.set(lb=0.0, ub=1.00, par0=0.020, unit=None, description='Amplitude of pairwise pathway #1' )
    Vmultispin.lam23.set(lb=0.0, ub=1.00, par0=0.002, unit=None, description='Amplitude of pairwise pathways #2 and #3' )
    Vmultispin.conc_decay.set(lb=0, ub=500, par0=40, unit='μM', description='Spin concentration / Decay rate' )
    Vmultispin.d.set(lb=0, ub=6, par0=1, unit='μM', description='Fractal dimension' )
    if not fractal:
        Vmultispin.d.freeze(1)
        Vmultispin.conc_decay.set(lb=0, ub=500, par0=40, unit='μM', description='Spin concentration / Decay rate' )
    else:
        Vmultispin.conc_decay.set(lb=0, ub=5, par0=0.05, unit='μM', description='Spin concentration / Decay rate' )

    return Vmultispin
#------------------------------------------------------------------------------
```

---

## Triradical T111¶

---

In [ ]:

```
threespin=True
fractal=True

# Load experimental data
dBs = [7,5,4,3,2,0]
HSCfile=r'.\\HSC_simulations\\HSCsim_T111_100000samples.txt'
files = [f'.\\data\\triradical_T111_DEER_{dB}dB.DTA' for dB in dBs]
Vmodels,Vexps,ts,Vexps_sub,ts_sub = [],[],[],[],[]
for n,file in enumerate(files):
    with warnings.catch_warnings():
        warnings.simplefilter("ignore")
        t,Vexp, descriptor = dl.deerload(file,full_output=True)
        t0, tau1_exp, tau2_exp = get_experimental_taus(descriptor)
    t += t0

    # Pre-processing
    Vexp = Vexp[:-10]
    t = t[:-10]
    Vexp = dl.correctphase(Vexp)
    Vexp /= np.max(Vexp) 
    Vexps.append(Vexp)
    ts.append(t) 

    # Subsampling
    sampling = np.arange(0,len(t),2)
    t_sub = t[sampling]
    Vexp_sub = Vexp[sampling]
    Vexps_sub.append(Vexp_sub)
    ts_sub.append(t_sub) 

    # Construct the dipolar multispin model
    Vmodels.append(construct_threespin_dipolarmodel(t_sub,tau1_exp,tau2_exp, threespin=threespin, fractal=fractal))

Vglobal = dl.merge(*Vmodels)
Vglobal = dl.link(Vglobal,
        mean1=[f'mean1_{n+1}' for n in range(len(Vmodels))],
        mean2=[f'mean2_{n+1}' for n in range(len(Vmodels))],
        mean3=[f'mean3_{n+1}' for n in range(len(Vmodels))],
        chol11=[f'chol11_{n+1}' for n in range(len(Vmodels))],
        chol22=[f'chol22_{n+1}' for n in range(len(Vmodels))],    
        chol33=[f'chol33_{n+1}' for n in range(len(Vmodels))],    
        chol21=[f'chol21_{n+1}' for n in range(len(Vmodels))],    
        chol31=[f'chol31_{n+1}' for n in range(len(Vmodels))],    
        chol32=[f'chol32_{n+1}' for n in range(len(Vmodels))],    
        tau1=[f'tau1_{n+1}' for n in range(len(Vmodels))],    
        tau2=[f'tau2_{n+1}' for n in range(len(Vmodels))],    
        conc_decay=[f'conc_decay_{n+1}' for n in range(len(Vmodels))],  
        d=[f'd_{n+1}' for n in range(len(Vmodels))],    
        lam23=[f'lam23_{n+1}' for n in range(len(Vmodels))],      
)

# Use the HSC simulation to get a good starting point
HSCdata = np.loadtxt(HSCfile)
for n in range(HSCdata.shape[1]):
    hist,bins = np.histogram(HSCdata[:,n],bins=200)
    bins = bins[:-1] + (bins[1]-bins[0])/2 
    fit = dl.fit(dl.dd_gauss,hist,bins)
    getattr(Vglobal,f'mean{n+1}').par0 = fit.mean
    getattr(Vglobal,f'chol{n+1}{n+1}').par0 = fit.std
corrs = get_corrs(HSCfile)
Vglobal.chol21.par0 = corrs[0]
Vglobal.chol31.par0 = corrs[1]
Vglobal.chol32.par0 = corrs[2]

# No 2+1 contributions
Vglobal.lam23.freeze(0)

# Fit the model to the data 
results = dl.fit(Vglobal, Vexps_sub, reg=False, ftol=1e-5, max_nfev=100) 

# Print the fit summary 
print(results)
 
# Plot the multivariate distance distribution
means = [results.mean1, results.mean2, results.mean3]
cholesky_factors = [results.chol11,results.chol22,results.chol33,results.chol21, results.chol31, results.chol32] 

plot_multivariate(means,cholesky_factors,xlim=[0,8], saveas='DEER_global_T111_Pfit.svg', HSCfile=HSCfile)

lamus = [getattr(results,f'lamu_{n+1}') for n in range(len(Vmodels))]
lam1s = [getattr(results,f'lam1_{n+1}') for n in range(len(Vmodels))]
lam23s = [results.lam23]*len(Vmodels)
params = [[ts[n],means,cholesky_factors,results.tau1,results.tau2,lamus[n],lam1s[n],lam23s[n],results.conc_decay,results.d,threespin,fractal] for n in range(len(Vmodels))]
scales = [getattr(results,f'scale_{n+1}') for n in range(len(Vmodels))]

plot_dataset_fit(ts,Vexps,threespin_dipolarsignal,params,scales,saveas='DEER_global_T111_Vfit.svg')
```

```
Goodness-of-fit: 
========= ============= ============ ======= =========== 
 Dataset   Noise level   Reduced 𝛘2   RMSD       AIC     
========= ============= ============ ======= =========== 
   #1         0.002        5.249      0.004   -1920.826  
   #2         0.001        11.465     0.005   -1785.179  
   #3         0.002        9.042      0.005   -1689.361  
   #4         0.002        7.355      0.006   -1600.525  
   #5         0.002        9.332      0.006   -1635.156  
   #6         0.001        16.154     0.005   -1703.425  
========= ============= ============ ======= =========== 
Model parameters: 
============ ======== ========================= ====== ======================================================= 
 Parameter    Value    95%-Confidence interval   Unit   Description                                            
============ ======== ========================= ====== ======================================================= 
 mean1        3.481    (3.396,3.565)              nm    Average inter-spin distance  #1                        
 mean2        3.460    (3.327,3.594)              nm    Average inter-spin distance  #2                        
 mean3        3.537    (3.420,3.654)              nm    Average inter-spin distance  #3                        
 chol11       0.464    (0.464,0.464)              nm    Cholesky factor ℓ11                                    
 chol22       0.462    (0.462,0.463)              nm    Cholesky factor ℓ22                                    
 chol33       0.461    (0.461,0.461)              nm    Cholesky factor ℓ33                                    
 chol21       0.002    (0.002,0.002)              nm    Cholesky factor ℓ21                                    
 chol31       0.000    (0.000,0.000)              nm    Cholesky factor ℓ31                                    
 chol32       -0.001   (-0.001,-0.000)            nm    Cholesky factor ℓ32                                    
 tau1         0.411    (0.411,0.412)              μs    First inter-pulse delay                                
 tau2         6.099    (6.099,6.099)              μs    Second inter-pulse delay                               
 lamu_1       1.077    (0.720,1.433)             None   Amplitude of unmodulated pairwise pathway              
 lam1_1       0.031    (0.012,0.049)             None   Amplitude of pairwise pathway #1                       
 lam23        0.000    (frozen)                  None   Amplitude of pairwise pathways #2 and #3               
 conc_decay   0.144    (0.128,0.160)              μM    Spin concentration / Decay rate                        
 d            1.368    (1.322,1.414)              μM    Fractal dimension                                      
 lamu_2       1.092    (0.831,1.353)             None   Amplitude of unmodulated pairwise pathway              
 lam1_2       0.045    (0.026,0.064)             None   Amplitude of pairwise pathway #1                       
 lamu_3       1.069    (0.865,1.272)             None   Amplitude of unmodulated pairwise pathway              
 lam1_3       0.057    (0.038,0.076)             None   Amplitude of pairwise pathway #1                       
 lamu_4       1.027    (0.870,1.184)             None   Amplitude of unmodulated pairwise pathway              
 lam1_4       0.072    (0.054,0.091)             None   Amplitude of pairwise pathway #1                       
 lamu_5       1.034    (0.909,1.158)             None   Amplitude of unmodulated pairwise pathway              
 lam1_5       0.084    (0.067,0.101)             None   Amplitude of pairwise pathway #1                       
 lamu_6       0.949    (0.873,1.026)             None   Amplitude of unmodulated pairwise pathway              
 lam1_6       0.111    (0.098,0.125)             None   Amplitude of pairwise pathway #1                       
 scale_1      1.005    (1.004,1.005)             None   Echo amplitude in the abscense of dipolar modulations  
 scale_2      1.006    (1.005,1.006)             None   Echo amplitude in the abscense of dipolar modulations  
 scale_3      1.007    (1.006,1.009)             None   Echo amplitude in the abscense of dipolar modulations  
 scale_4      1.003    (1.001,1.006)             None   Echo amplitude in the abscense of dipolar modulations  
 scale_5      1.001    (0.996,1.005)             None   Echo amplitude in the abscense of dipolar modulations  
 scale_6      0.995    (0.992,0.998)             None   Echo amplitude in the abscense of dipolar modulations  
============ ======== ========================= ====== =======================================================
```

---

## Triradial T011¶

---

In [ ]:

```
threespin=True
fractal=True

# Load experimental data
dBs = [11,9,8,7,5,3,0]
HSCfile=r'.\\HSC_simulations\\HSCsim_triradical_T011_100000samples.txt'
files = [f'.\\data\\triradical_T011_DEER_{dB}dB.DTA' for dB in dBs]
Vmodels,Vexps,ts,Vexps_sub,ts_sub = [],[],[],[],[]
for n,file in enumerate(files):
    with warnings.catch_warnings():
        warnings.simplefilter("ignore")
        t,Vexp, descriptor = dl.deerload(file,full_output=True)
        t0, tau1_exp, tau2_exp = get_experimental_taus(descriptor)
    t += t0

    # Pre-processing
    Vexp = Vexp[:-10]
    t = t[:-10]
    Vexp = dl.correctphase(Vexp)
    Vexp /= np.max(Vexp) 
    Vexps.append(Vexp)
    ts.append(t) 

    # Subsampling
    sampling = np.arange(0,len(t),2)
    t_sub = t[sampling]
    Vexp_sub = Vexp[sampling]
    Vexps_sub.append(Vexp_sub)
    ts_sub.append(t_sub) 

    # Construct the dipolar multispin model
    Vmodels.append(construct_threespin_dipolarmodel(t_sub,tau1_exp,tau2_exp, threespin=threespin, fractal=fractal))

Vglobal = dl.merge(*Vmodels)
Vglobal = dl.link(Vglobal,
        mean1=[f'mean1_{n+1}' for n in range(len(Vmodels))],
        mean2=[f'mean2_{n+1}' for n in range(len(Vmodels))],
        mean3=[f'mean3_{n+1}' for n in range(len(Vmodels))],
        chol11=[f'chol11_{n+1}' for n in range(len(Vmodels))],
        chol22=[f'chol22_{n+1}' for n in range(len(Vmodels))],    
        chol33=[f'chol33_{n+1}' for n in range(len(Vmodels))],    
        chol21=[f'chol21_{n+1}' for n in range(len(Vmodels))],    
        chol31=[f'chol31_{n+1}' for n in range(len(Vmodels))],    
        chol32=[f'chol32_{n+1}' for n in range(len(Vmodels))],    
        tau1=[f'tau1_{n+1}' for n in range(len(Vmodels))],    
        tau2=[f'tau2_{n+1}' for n in range(len(Vmodels))],    
        conc_decay=[f'conc_decay_{n+1}' for n in range(len(Vmodels))],  
        d=[f'd_{n+1}' for n in range(len(Vmodels))],    
        lam23=[f'lam23_{n+1}' for n in range(len(Vmodels))],      
)

# Use the HSC simulation to get a good starting point
HSCdata = np.loadtxt(HSCfile)
for n in range(HSCdata.shape[1]):
    hist,bins = np.histogram(HSCdata[:,n],bins=200)
    bins = bins[:-1] + (bins[1]-bins[0])/2 
    fit = dl.fit(dl.dd_gauss,hist,bins)
    getattr(Vglobal,f'mean{n+1}').par0 = fit.mean
    getattr(Vglobal,f'chol{n+1}{n+1}').par0 = fit.std

corrs = get_corrs(HSCfile)
Vglobal.chol21.par0 = corrs[0]
Vglobal.chol31.par0 = corrs[1]
Vglobal.chol32.par0 = corrs[2]

Vglobal.lam23.freeze(0)

# Fit the model to the data 
results = dl.fit(Vglobal, Vexps_sub, reg=False, ftol=1e-5, max_nfev=100) 

# Print the fit summary 
print(results)
 
# Plot the multivariate distance distribution
means = [results.mean1, results.mean2, results.mean3]
cholesky_factors = [results.chol11,results.chol22,results.chol33,results.chol21, results.chol31, results.chol32] 

plot_multivariate(means,cholesky_factors,xlim=[0,8], saveas='DEER_global_T011_Pfit.svg', HSCfile=HSCfile)

lamus = [getattr(results,f'lamu_{n+1}') for n in range(len(Vmodels))]
lam1s = [getattr(results,f'lam1_{n+1}') for n in range(len(Vmodels))]
lam23s = [results.lam23]*len(Vmodels)
params = [[ts[n],means,cholesky_factors,results.tau1,results.tau2,lamus[n],lam1s[n],lam23s[n],results.conc_decay,results.d,threespin,fractal] for n in range(len(Vmodels))]
scales = [getattr(results,f'scale_{n+1}') for n in range(len(Vmodels))]

plot_dataset_fit(ts,Vexps,threespin_dipolarsignal,params,scales,saveas='DEER_global_T011_Vfit.svg')
```

```
Goodness-of-fit: 
========= ============= ============ ======= =========== 
 Dataset   Noise level   Reduced 𝛘2   RMSD       AIC     
========= ============= ============ ======= =========== 
   #1         0.002        3.252      0.004   -2463.820  
   #2         0.004        1.673      0.005   -2162.126  
   #3         0.004        1.238      0.005   -2239.828  
   #4         0.006        1.021      0.006   -2032.513  
   #5         0.003        1.852      0.004   -2376.629  
   #6         0.003        1.869      0.003   -2583.196  
   #7         0.002        1.412      0.003   -2758.198  
========= ============= ============ ======= =========== 
Model parameters: 
============ ======== ========================= ====== ======================================================= 
 Parameter    Value    95%-Confidence interval   Unit   Description                                            
============ ======== ========================= ====== ======================================================= 
 mean1        3.121    (3.093,3.150)              nm    Average inter-spin distance  #1                        
 mean2        3.123    (3.097,3.149)              nm    Average inter-spin distance  #2                        
 mean3        3.091    (3.055,3.127)              nm    Average inter-spin distance  #3                        
 chol11       0.465    (0.465,0.465)              nm    Cholesky factor ℓ11                                    
 chol22       0.436    (0.436,0.436)              nm    Cholesky factor ℓ22                                    
 chol33       0.437    (0.437,0.437)              nm    Cholesky factor ℓ33                                    
 chol21       0.035    (0.034,0.035)              nm    Cholesky factor ℓ21                                    
 chol31       -0.023   (-0.023,-0.022)            nm    Cholesky factor ℓ31                                    
 chol32       0.042    (0.042,0.042)              nm    Cholesky factor ℓ32                                    
 tau1         0.418    (0.417,0.418)              μs    First inter-pulse delay                                
 tau2         12.099   (12.099,12.099)            μs    Second inter-pulse delay                               
 lamu_1       0.908    (0.813,1.002)             None   Amplitude of unmodulated pairwise pathway              
 lam1_1       0.121    (0.102,0.140)             None   Amplitude of pairwise pathway #1                       
 lam23        0.000    (frozen)                  None   Amplitude of pairwise pathways #2 and #3               
 conc_decay   0.251    (0.237,0.266)              μM    Spin concentration / Decay rate                        
 d            1.246    (1.236,1.256)              μM    Fractal dimension                                      
 lamu_2       1.013    (0.881,1.146)             None   Amplitude of unmodulated pairwise pathway              
 lam1_2       0.095    (0.075,0.115)             None   Amplitude of pairwise pathway #1                       
 lamu_3       1.145    (0.959,1.330)             None   Amplitude of unmodulated pairwise pathway              
 lam1_3       0.073    (0.053,0.094)             None   Amplitude of pairwise pathway #1                       
 lamu_4       1.176    (0.945,1.406)             None   Amplitude of unmodulated pairwise pathway              
 lam1_4       0.060    (0.039,0.080)             None   Amplitude of pairwise pathway #1                       
 lamu_5       1.169    (0.900,1.438)             None   Amplitude of unmodulated pairwise pathway              
 lam1_5       0.050    (0.030,0.070)             None   Amplitude of pairwise pathway #1                       
 lamu_6       1.007    (0.793,1.220)             None   Amplitude of unmodulated pairwise pathway              
 lam1_6       0.047    (0.030,0.065)             None   Amplitude of pairwise pathway #1                       
 lamu_7       0.839    (0.643,1.035)             None   Amplitude of unmodulated pairwise pathway              
 lam1_7       0.037    (0.022,0.051)             None   Amplitude of pairwise pathway #1                       
 scale_1      1.010    (1.008,1.012)             None   Echo amplitude in the abscense of dipolar modulations  
 scale_2      1.001    (1.000,1.001)             None   Echo amplitude in the abscense of dipolar modulations  
 scale_3      1.000    (0.994,1.006)             None   Echo amplitude in the abscense of dipolar modulations  
 scale_4      1.014    (1.002,1.025)             None   Echo amplitude in the abscense of dipolar modulations  
 scale_5      1.008    (0.991,1.024)             None   Echo amplitude in the abscense of dipolar modulations  
 scale_6      1.018    (1.017,1.019)             None   Echo amplitude in the abscense of dipolar modulations  
 scale_7      1.010    (1.010,1.011)             None   Echo amplitude in the abscense of dipolar modulations  
============ ======== ========================= ====== =======================================================
```

---

## Rpo47 (global)¶

---

In [ ]:

```
threespin = True
fractal = False

# Load experimental data
dBs = [0,6,9]
files = [f'.\\data\\triradical_rpo47_DEER_{dB}dB.DTA' for dB in dBs]
MMMfile = r".\\MMMx_simulations\\MMMx_Rpo47_triple_labelled_sampled.mat"
Vmodels,Vexps,ts,Vexps_sub,ts_sub = [],[],[],[],[]
for n,file in enumerate(files):
    with warnings.catch_warnings():
        warnings.simplefilter("ignore")
        t,Vexp, descriptor = dl.deerload(file,full_output=True)
        t0, tau1_exp, tau2_exp = get_experimental_taus(descriptor)
    t += t0

    # Pre-processing
    Vexp = dl.correctphase(Vexp)
    Vexp /= np.max(Vexp) 
    Vexps.append(Vexp)
    ts.append(t) 

    # Subsampling
    sampling = np.arange(0,len(t),1)
    t_sub = t[sampling]
    Vexp_sub = Vexp[sampling]
    Vexps_sub.append(Vexp_sub)
    ts_sub.append(t_sub) 

    # Construct the dipolar multispin model
    Vmodels.append(construct_threespin_dipolarmodel(t_sub,tau1_exp,tau2_exp, threespin=threespin, fractal=fractal))

Vglobal = dl.merge(*Vmodels)
Vglobal = dl.link(Vglobal,
        mean1=[f'mean1_{n+1}' for n in range(len(Vmodels))],
        mean2=[f'mean2_{n+1}' for n in range(len(Vmodels))],
        mean3=[f'mean3_{n+1}' for n in range(len(Vmodels))],
        chol11=[f'chol11_{n+1}' for n in range(len(Vmodels))],
        chol22=[f'chol22_{n+1}' for n in range(len(Vmodels))],    
        chol33=[f'chol33_{n+1}' for n in range(len(Vmodels))],    
        chol21=[f'chol21_{n+1}' for n in range(len(Vmodels))],    
        chol31=[f'chol31_{n+1}' for n in range(len(Vmodels))],    
        chol32=[f'chol32_{n+1}' for n in range(len(Vmodels))],    
        tau1=[f'tau1_{n+1}' for n in range(len(Vmodels))],    
        tau2=[f'tau2_{n+1}' for n in range(len(Vmodels))],    
        conc_decay=[f'conc_decay_{n+1}' for n in range(len(Vmodels))],  
        d=[f'd_{n+1}' for n in range(len(Vmodels))],    
)

# Use the HSC simulation to get a good starting point
from scipy.io import loadmat
MMMdata = loadmat(MMMfile)['rlist']
for n in range(MMMdata.shape[1]):
    hist,bins = np.histogram(MMMdata[:,n],bins=200)
    bins = bins[:-1] + (bins[1]-bins[0])/2 
    fit = dl.fit(dl.dd_gauss,hist,bins)
    getattr(Vglobal,f'mean{n+1}').par0 = fit.mean
    getattr(Vglobal,f'chol{n+1}{n+1}').par0 = fit.std
corrs = get_corrs(MMMfile)
Vglobal.chol21.par0 = corrs[0]
Vglobal.chol31.par0 = corrs[1]
Vglobal.chol32.par0 = corrs[2]

Vglobal.conc_decay.par0 = 100

# Fit the model to the data 
results = dl.fit(Vglobal, Vexps_sub, reg=False, ftol=1e-5, max_nfev=100) 

# Print the fit summary 
print(results)
 
# Plot the multivariate distance distribution
means = [results.mean1, results.mean2, results.mean3]
cholesky_factors = [results.chol11,results.chol22,results.chol33,results.chol21, results.chol31, results.chol32] 

plot_multivariate(means,cholesky_factors,xlim=[0,8], saveas='DEER_global_Rpo47_Pfit.svg', MMMfile=MMMfile)

lamus = [getattr(results,f'lamu_{n+1}') for n in range(len(Vmodels))]
lam1s = [getattr(results,f'lam1_{n+1}') for n in range(len(Vmodels))]
lam23s = [getattr(results,f'lam23_{n+1}') for n in range(len(Vmodels))]
params = [[ts[n],means,cholesky_factors,results.tau1,results.tau2,lamus[n],lam1s[n],lam23s[n],results.conc_decay,results.d,threespin,fractal] for n in range(len(Vmodels))]
scales = [getattr(results,f'scale_{n+1}') for n in range(len(Vmodels))]

plot_dataset_fit(ts,Vexps,threespin_dipolarsignal,params,scales,saveas='DEER_global_Rpo47_Vfit.svg')
```

```
d:\lufa\projects\deerlab\deerlab\deerlab\utils\gof.py:53: RuntimeWarning: divide by zero encountered in double_scalars
  chi2red = 1/Ndof*np.linalg.norm(x - xfit)**2/sigma**2
```

```
Goodness-of-fit: 
========= ============= ============ ======= =========== 
 Dataset   Noise level   Reduced 𝛘2   RMSD       AIC     
========= ============= ============ ======= =========== 
   #1         0.013        1.787      0.017   -1171.946  
   #2         0.010        1.823      0.014   -1516.456  
   #3         0.015        1.257      0.017   -1179.920  
========= ============= ============ ======= =========== 
Model parameters: 
============ ========= ========================= ====== ======================================================= 
 Parameter    Value     95%-Confidence interval   Unit   Description                                            
============ ========= ========================= ====== ======================================================= 
 mean1        2.404     (2.322,2.485)              nm    Average inter-spin distance  #1                        
 mean2        4.265     (4.180,4.350)              nm    Average inter-spin distance  #2                        
 mean3        6.264     (6.145,6.382)              nm    Average inter-spin distance  #3                        
 chol11       0.449     (0.449,0.450)              nm    Cholesky factor ℓ11                                    
 chol22       0.332     (0.328,0.337)              nm    Cholesky factor ℓ22                                    
 chol33       0.281     (0.245,0.317)              nm    Cholesky factor ℓ33                                    
 chol21       -0.012    (-0.043,0.019)             nm    Cholesky factor ℓ21                                    
 chol31       0.144     (0.096,0.193)              nm    Cholesky factor ℓ31                                    
 chol32       0.137     (0.087,0.187)              nm    Cholesky factor ℓ32                                    
 tau1         0.404     (0.401,0.408)              μs    First inter-pulse delay                                
 tau2         9.006     (8.900,9.100)              μs    Second inter-pulse delay                               
 lamu_1       0.930     (0.365,1.495)             None   Amplitude of unmodulated pairwise pathway              
 lam1_1       0.051     (0.000,0.102)             None   Amplitude of pairwise pathway #1                       
 lam23_1      0.010     (0.000,0.027)             None   Amplitude of pairwise pathways #2 and #3               
 conc_decay   101.089   (89.530,112.649)           μM    Spin concentration / Decay rate                        
 d            1.000     (frozen)                   μM    Fractal dimension                                      
 lamu_2       1.012     (0.186,1.837)             None   Amplitude of unmodulated pairwise pathway              
 lam1_2       0.035     (0.000,0.087)             None   Amplitude of pairwise pathway #1                       
 lam23_2      0.006     (0.000,0.018)             None   Amplitude of pairwise pathways #2 and #3               
 lamu_3       1.014     (0.000,2.000)             None   Amplitude of unmodulated pairwise pathway              
 lam1_3       0.027     (0.000,0.079)             None   Amplitude of pairwise pathway #1                       
 lam23_3      0.002     (0.000,0.009)             None   Amplitude of pairwise pathways #2 and #3               
 scale_1      1.197     (1.190,1.204)             None   Echo amplitude in the abscense of dipolar modulations  
 scale_2      1.146     (1.146,1.147)             None   Echo amplitude in the abscense of dipolar modulations  
 scale_3      1.074     (1.072,1.075)             None   Echo amplitude in the abscense of dipolar modulations  
============ ========= ========================= ====== =======================================================
```

## Model (Four spins)¶

In [ ]:

```
#------------------------------------------------------------------------------
def fourspin_dipolarsignal(t,means,cholfactors,tau1,tau2,lamu,lam1,lam23,conc_decay,d,threespin=False,fractal=False):

    trefs,δs,λs= [],[],[]
    Npermutations = 720
    ### Two-spin contributions
    tref = [ tau1, None, None, None, None, None]
    δ    = [    1,    0,    0,    0,    0,    0]
    trefs = add_circlularpermutations(trefs,tref)
    δs = add_circlularpermutations(δs,δ)
    λs += [Npermutations/6*lam1*lamu**5]*6

    if lam23>0:
        tref = [ tau1+tau2, None, None, None, None, None]
        δ    = [         1,    0,    0,    0,    0,    0]
        trefs = add_circlularpermutations(trefs,tref)
        δs = add_circlularpermutations(δs,δ)
        λs += [Npermutations/6*lam23*lamu**5]*6

        tref = [ 0, None, None, None, None, None]
        δ    = [ 1,    0,    0,    0,    0,    0]
        trefs = add_circlularpermutations(trefs,tref)
        δs = add_circlularpermutations(δs,δ)
        λs += [Npermutations/6*lam23*lamu**5]*6

    ### Three-spin pathways
    if threespin:
        # Pathways (4)
        tref = [ tau1, tau1, None, None, None, None]
        δ    = [    1,    1,    0,    0,    0,    0]
        trefs = add_permutations(trefs,tref)
        δs = add_permutations(δs,δ)
        _,idx = np.unique(np.array(δs), axis=0, return_index=True)
        trefs = [trefs[i] for i in idx]
        δs = [δs[i] for i in idx]
        λs += [Npermutations/len(δs)*2*lam1*lam1*lamu**4]*len(δs)


    # Monte-Carlo multivariate distance integration
    Nsamples = 10000
    np.random.seed(seed=1)
    rsamples = Pmultivar(means,cholfactors).rvs(Nsamples)
    rsamples = np.maximum(rsamples,1e-16) # Avoid values exactly at zero 

    # Triangle inequalities
    Ndistances = np.shape(rsamples)[1]
    triangles = [[0,1,5],[0,3,4],[1,2,4],[2,3,5]]
    for triangle in triangles: # Loop over all triangle combinations
        idx = triangle
        triangle_condition = np.full_like(rsamples,False)
        for n in range(Ndistances): # Evaluate all triangle inequalities
            idx = np.roll(idx,-1,axis=0)
            triangle_condition[:,n] = np.sum(rsamples[:,idx[:-1]],axis=1) > rsamples[:,idx[-1]]
        triangle_condition = np.all(triangle_condition,axis=1)
        # Discard samples that do not satisfy triangle inequalities
        rsamples = rsamples[triangle_condition,:]

    # Construct the dipolar signal 
    λ0 = np.maximum(1 - np.sum(λs),0)
    Vintra,Vinter = λ0,1
    twospin_contributions,threespin_contributions = [],[]
    # Loop over all dipolar pathways
    for δ,tref,λ in zip(δs,trefs,λs):

        # Set trefs defined as None to an arbitrary numerical value
        tref = [tref[n] if δn!=0 else 0 for n,δn in enumerate(δ)]

        # Number of spins participating in the pathway
        Nspin = np.sum(δ)+1 

        if Nspin==2:
            n = int(np.where(δ==1)[0])
            # Estimate the 1D-marginal distance distribution
            Pmarginal,bins = np.histogram(rsamples[:,n], bins=100)
            rgrid = (bins[:-1] + bins[1:])/2
            Pmarginal = Pmarginal/np.trapz(Pmarginal,rgrid)

            # Two-spin intramolecular contribution
            Vintra_contr = dl.dipolarkernel(δ[n]*(t-tref[n]),rgrid)@Pmarginal
            twospin_contributions.append(λ*Vintra_contr)

        if Nspin==3:
            # Non-uniformly subsample the time-domain (speedup)
            n = int(np.where(δ==1)[0][0])
            np.random.seed(0)
            subsampling = np.exp(-6*abs(t-tref[n])/max(t))*np.random.rand(len(t))>0.15
            subsampling[-1] = 1
            tsub = t[subsampling]
            # Average three-spin contribution over all distances
            Nsamples = 80
            n1,n2 = np.where(δ==1)[0].astype(int)

            if (n1 in [4,5] and n2 in [4,5]) or (n1 in [0,2] and n2 in [0,2]) or (n1 in [1,3] and n2 in [1,3]):
                continue
            idx = np.where([n1 in triangle and n2 in triangle for triangle in triangles])[0][0]
            triangle = triangles[idx].copy()
            triangle.remove(n1)
            triangle.remove(n2)
            n3 = triangle
            Vintra_contr = 0
            for rs in rsamples[:Nsamples,:]: 
                # Three-spin intramolecular contribution
                Vintra_contr += threespin_dipolarkernel(tsub,rs[n1],rs[n2],rs[n3],[δ[n1],δ[n2],0],[tref[n1],tref[n2],0]) 
            Vintra_contr /= Nsamples
            Vintra_contr = interp1d(tsub,Vintra_contr, fill_value='extrapolate')(t)
            threespin_contributions.append(λ*Vintra_contr)

        Vintra += λ*Vintra_contr

        # Intermolecular contribution
        Vinter *= Bbasis(t,λ,conc_decay,d,δ,tref,fractal=fractal) 

    Vinter /= np.max(Vinter)
    V = Vintra*Vinter

    return V,λ0,Vinter,Vintra,twospin_contributions,threespin_contributions
#------------------------------------------------------------------------------

#------------------------------------------------------------------------------
def construct_fourspin_dipolarmodel(t,tau1_exp,tau2_exp,threespin=False,fractal=False):

    def _fourspin_dipolarsignal(mean1,mean2,mean3,mean4,mean5,mean6,chol11,chol22,chol33,chol44,chol55,chol66,
                                chol21,chol31,chol41,chol51,chol61,chol32,chol42,chol52,chol62,chol43,chol53,
                                chol63,chol54,chol64,chol65,
                                tau1,tau2,lamu,lam1,lam23,conc_decay,d):
        means  = [mean1,mean2,mean3,mean4,mean5,mean6]
        cholfactors = [chol11,chol22,chol33,chol44,chol55,chol66,chol21,chol31,chol41,chol51,chol61,chol32,chol42,chol52,chol62,chol43,chol53,chol63,chol54,chol64,chol65]
        Vfit = fourspin_dipolarsignal(t,means,cholfactors,tau1,tau2,lamu,lam1,lam23,conc_decay,d,threespin=threespin, fractal=fractal)[0]
        return Vfit 
    # Construct model 
    Vmultispin = dl.Model(_fourspin_dipolarsignal)
    Vmultispin.description = 'Multi-spin dipolar signal arising from a DEER experiment'
    # Set parameter properties
    Vmultispin.addlinear('scale',lb=0, description='Echo amplitude in the abscense of dipolar modulations')
    Vmultispin.mean1.set(lb=1.0, ub=8.0, par0=4.0, unit='nm', description='Average inter-spin distance  #1')
    Vmultispin.mean2.set(lb=1.0, ub=8.0, par0=4.0, unit='nm', description='Average inter-spin distance  #2')
    Vmultispin.mean3.set(lb=1.0, ub=8.0, par0=3.0, unit='nm', description='Average inter-spin distance  #3')
    Vmultispin.mean4.set(lb=1.0, ub=8.0, par0=3.0, unit='nm', description='Average inter-spin distance  #4')
    Vmultispin.mean5.set(lb=1.0, ub=8.0, par0=2.0, unit='nm', description='Average inter-spin distance  #5')
    Vmultispin.mean6.set(lb=1.0, ub=8.0, par0=2.0, unit='nm', description='Average inter-spin distance  #6')
    Vmultispin.chol11.set(lb=0.1, ub=0.8, par0=0.40, unit='nm', description='Cholesky factor ℓ11' )
    Vmultispin.chol22.set(lb=0.1, ub=0.8, par0=0.40, unit='nm', description='Cholesky factor ℓ22' )
    Vmultispin.chol33.set(lb=0.1, ub=0.8, par0=0.40, unit='nm', description='Cholesky factor ℓ33' )
    Vmultispin.chol44.set(lb=0.1, ub=0.8, par0=0.40, unit='nm', description='Cholesky factor ℓ44' )
    Vmultispin.chol55.set(lb=0.1, ub=0.8, par0=0.40, unit='nm', description='Cholesky factor ℓ55' )
    Vmultispin.chol66.set(lb=0.1, ub=0.8, par0=0.40, unit='nm', description='Cholesky factor ℓ66' )
    Vmultispin.chol21.set(lb=-1.0, ub=1.0, par0=0.0, unit='nm', description='Cholesky factor ℓ21' )
    Vmultispin.chol31.set(lb=-1.0, ub=1.0, par0=0.0, unit='nm', description='Cholesky factor ℓ31' )
    Vmultispin.chol41.set(lb=-1.0, ub=1.0, par0=0.0, unit='nm', description='Cholesky factor ℓ41' )
    Vmultispin.chol51.set(lb=-1.0, ub=1.0, par0=0.0, unit='nm', description='Cholesky factor ℓ51' )
    Vmultispin.chol61.set(lb=-1.0, ub=1.0, par0=0.0, unit='nm', description='Cholesky factor ℓ61' )
    Vmultispin.chol32.set(lb=-1.0, ub=1.0, par0=0.0, unit='nm', description='Cholesky factor ℓ32' )
    Vmultispin.chol42.set(lb=-1.0, ub=1.0, par0=0.0, unit='nm', description='Cholesky factor ℓ42' )
    Vmultispin.chol52.set(lb=-1.0, ub=1.0, par0=0.0, unit='nm', description='Cholesky factor ℓ52' )
    Vmultispin.chol62.set(lb=-1.0, ub=1.0, par0=0.0, unit='nm', description='Cholesky factor ℓ62' )
    Vmultispin.chol43.set(lb=-1.0, ub=1.0, par0=0.0, unit='nm', description='Cholesky factor ℓ43' )
    Vmultispin.chol53.set(lb=-1.0, ub=1.0, par0=0.0, unit='nm', description='Cholesky factor ℓ53' )
    Vmultispin.chol63.set(lb=-1.0, ub=1.0, par0=0.0, unit='nm', description='Cholesky factor ℓ63' )
    Vmultispin.chol54.set(lb=-1.0, ub=1.0, par0=0.0, unit='nm', description='Cholesky factor ℓ54' )
    Vmultispin.chol65.set(lb=-1.0, ub=1.0, par0=0.0, unit='nm', description='Cholesky factor ℓ65' )
    Vmultispin.tau1.set(lb=tau1_exp-0.10, ub=tau1_exp+0.10, par0=tau1_exp, unit='μs', description='First inter-pulse delay' )
    Vmultispin.tau2.set(lb=tau2_exp-0.10, ub=tau2_exp+0.10, par0=tau2_exp, unit='μs', description='Second inter-pulse delay' )
    Vmultispin.lamu.set(lb=0.0, ub=10.00, par0=1, unit=None, description='Amplitude of unmodulated pairwise pathway')
    Vmultispin.lam1.set(lb=0.0, ub=1.00, par0=0.0005, unit=None, description='Amplitude of pairwise pathway #1' )
    Vmultispin.lam23.set(lb=0.0, ub=1.00, par0=0.0002, unit=None, description='Amplitude of pairwise pathways #2 and #3' )
    Vmultispin.conc_decay.set(lb=0, ub=500, par0=40, unit='μM', description='Spin concentration / Decay rate' )
    Vmultispin.d.set(lb=0, ub=6, par0=1.4, unit='μM', description='Fractal dimension' )
    if not fractal:
        Vmultispin.d.freeze(1)
        Vmultispin.conc_decay.set(lb=0, ub=500, par0=40, unit='μM', description='Spin concentration / Decay rate' )
    else:
        Vmultispin.conc_decay.set(lb=0, ub=1, par0=0.5, unit='μM', description='Spin concentration / Decay rate' )

    return Vmultispin
#------------------------------------------------------------------------------
```

---

## Q5¶

---

In [ ]:

```
threespin = True
fractal = True

# Load experimental data
dBs = [11,13,15,17]
files = [f'.\\data\\tetraradical_DEER_{dB}dB.DSC' for dB in dBs]
HSCfile=r'.\\HSC_simulations\HSCsim_tetraradical_100000samples.txt'
Vmodels,Vexps,ts,Vexps_sub,ts_sub = [],[],[],[],[]
for file in files:
    t,Vexp, descriptor = dl.deerload(file,full_output=True)
    t0, tau1_exp, tau2_exp = get_experimental_taus(descriptor)
    t += t0

    # Pre-processing
    Vexp = dl.correctphase(Vexp)
    Vexp /= np.max(Vexp) 
    Vexps.append(Vexp)
    ts.append(t) 

    # Subsampling
    sampling = np.arange(0,len(t),3)
    t_sub = t[sampling]
    Vexp_sub = Vexp[sampling]
    Vexps_sub.append(Vexp_sub)
    ts_sub.append(t_sub) 

    # Construct the dipolar multispin model
    Vmodels.append(construct_fourspin_dipolarmodel(t_sub,tau1_exp,tau2_exp, threespin=threespin, fractal=fractal))

Vglobal = dl.merge(*Vmodels)
Vglobal = dl.link(Vglobal,
        mean1=[f'mean1_{n+1}' for n in range(len(Vmodels))],
        mean2=[f'mean2_{n+1}' for n in range(len(Vmodels))],
        mean3=[f'mean3_{n+1}' for n in range(len(Vmodels))],
        mean4=[f'mean4_{n+1}' for n in range(len(Vmodels))],
        mean5=[f'mean5_{n+1}' for n in range(len(Vmodels))],
        mean6=[f'mean6_{n+1}' for n in range(len(Vmodels))],    
        chol11=[f'chol11_{n+1}' for n in range(len(Vmodels))],  
        chol22=[f'chol22_{n+1}' for n in range(len(Vmodels))],    
        chol33=[f'chol33_{n+1}' for n in range(len(Vmodels))],    
        chol44=[f'chol44_{n+1}' for n in range(len(Vmodels))],    
        chol55=[f'chol55_{n+1}' for n in range(len(Vmodels))],    
        chol66=[f'chol66_{n+1}' for n in range(len(Vmodels))],    
        chol21=[f'chol21_{n+1}' for n in range(len(Vmodels))],    
        chol31=[f'chol31_{n+1}' for n in range(len(Vmodels))],    
        chol41=[f'chol41_{n+1}' for n in range(len(Vmodels))],    
        chol51=[f'chol51_{n+1}' for n in range(len(Vmodels))],    
        chol61=[f'chol61_{n+1}' for n in range(len(Vmodels))],    
        chol32=[f'chol32_{n+1}' for n in range(len(Vmodels))],    
        chol42=[f'chol42_{n+1}' for n in range(len(Vmodels))],    
        chol52=[f'chol52_{n+1}' for n in range(len(Vmodels))],    
        chol62=[f'chol62_{n+1}' for n in range(len(Vmodels))],    
        chol43=[f'chol43_{n+1}' for n in range(len(Vmodels))],    
        chol53=[f'chol53_{n+1}' for n in range(len(Vmodels))],    
        chol63=[f'chol63_{n+1}' for n in range(len(Vmodels))],    
        chol54=[f'chol54_{n+1}' for n in range(len(Vmodels))],    
        chol64=[f'chol64_{n+1}' for n in range(len(Vmodels))],    
        chol65=[f'chol65_{n+1}' for n in range(len(Vmodels))],    
        tau1=[f'tau1_{n+1}' for n in range(len(Vmodels))],    
        tau2=[f'tau2_{n+1}' for n in range(len(Vmodels))],    
        conc_decay=[f'conc_decay_{n+1}' for n in range(len(Vmodels))],    
        lam23=[f'lam23_{n+1}' for n in range(len(Vmodels))],     
        d=[f'd_{n+1}' for n in range(len(Vmodels))],    
)


# No 2+1 contributions
Vglobal.lam23.freeze(0)
#Vglobal.tau1.freeze(tau1_exp)
Vglobal.tau2.freeze(tau2_exp)


# Use the HSC simulation to get a good starting point
from scipy.io import loadmat
data = np.loadtxt(HSCfile)
for n in range(data.shape[1]):
    hist,bins = np.histogram(data[:,n],bins=200)
    bins = bins[:-1] + (bins[1]-bins[0])/2 
    fit = dl.fit(dl.dd_gauss,hist,bins)
    getattr(Vglobal,f'mean{n+1}').par0 = fit.mean
    getattr(Vglobal,f'chol{n+1}{n+1}').par0 = np.maximum(fit.std,getattr(Vglobal,f'chol{n+1}{n+1}').lb)
corrs = get_corrs_4spin(HSCfile)
Vglobal.chol21.par0 = corrs[0]
Vglobal.chol31.par0 = corrs[1]
Vglobal.chol41.par0 = corrs[2]
Vglobal.chol51.par0 = corrs[3]
Vglobal.chol61.par0 = corrs[4]
Vglobal.chol32.par0 = corrs[5]
Vglobal.chol42.par0 = corrs[6]
Vglobal.chol52.par0 = corrs[7]
Vglobal.chol62.par0 = corrs[8]
Vglobal.chol43.par0 = corrs[9]
Vglobal.chol53.par0 = corrs[10]
Vglobal.chol63.par0 = corrs[11]
Vglobal.chol54.par0 = corrs[12]
Vglobal.chol64.par0 = corrs[13]
Vglobal.chol65.par0 = corrs[14]

Vglobal.chol21.freeze(Vglobal.chol21.par0)
Vglobal.chol31.freeze(Vglobal.chol31.par0)
Vglobal.chol41.freeze(Vglobal.chol41.par0)
Vglobal.chol51.freeze(Vglobal.chol51.par0)
Vglobal.chol61.freeze(Vglobal.chol61.par0)
Vglobal.chol32.freeze(Vglobal.chol32.par0)
Vglobal.chol42.freeze(Vglobal.chol42.par0)
Vglobal.chol52.freeze(Vglobal.chol52.par0)
Vglobal.chol62.freeze(Vglobal.chol62.par0)
Vglobal.chol43.freeze(Vglobal.chol43.par0)
Vglobal.chol53.freeze(Vglobal.chol53.par0)
Vglobal.chol63.freeze(Vglobal.chol63.par0)
Vglobal.chol54.freeze(Vglobal.chol54.par0)
Vglobal.chol64.freeze(Vglobal.chol64.par0)
Vglobal.chol65.freeze(Vglobal.chol65.par0)

Vglobal.chol21.freeze(0)
Vglobal.chol31.freeze(0)
Vglobal.chol41.freeze(0)
Vglobal.chol51.freeze(0)
Vglobal.chol61.freeze(0)
Vglobal.chol32.freeze(0)
Vglobal.chol42.freeze(0)
Vglobal.chol52.freeze(0)
Vglobal.chol62.freeze(0)
Vglobal.chol43.freeze(0)
Vglobal.chol53.freeze(0)
Vglobal.chol63.freeze(0)
Vglobal.chol54.freeze(0)
Vglobal.chol64.freeze(0)
Vglobal.chol65.freeze(0)

# Fit the model to the data 
results = dl.fit(Vglobal, Vexps_sub, reg=False, ftol=1e-2, max_nfev=300, verbose=2) 

# Print the fit summary 
print(results) 
 
# Plot the multivariate distance distribution 
means  = [results.mean1, results.mean2, results.mean3, results.mean4, results.mean5, results.mean6] 
cholesky_factors = [results.chol11, results.chol22, results.chol33, results.chol44, results.chol55, results.chol66, 
                    results.chol21, results.chol31, results.chol41, results.chol51, results.chol61, 
                    results.chol32, results.chol42, results.chol52, results.chol62, 
                    results.chol43, results.chol53, results.chol63, 
                    results.chol54, results.chol64, 
                    results.chol65] 

plot_multivariate(means,cholesky_factors,xlim=[0,8], saveas='DEER_global_Q5_Pfit.svg',HSCfile=HSCfile)

lamus = [getattr(results,f'lamu_{n+1}') for n in range(len(Vmodels))]
lam1s = [getattr(results,f'lam1_{n+1}') for n in range(len(Vmodels))]
lam23s = [results.lam23]*len(Vmodels)
params = [[ts[n],means,cholesky_factors,results.tau1,results.tau2,lamus[n],lam1s[n],lam23s[n],results.conc_decay,results.d,threespin,fractal] for n in range(len(Vmodels))]
scales = [getattr(results,f'scale_{n+1}') for n in range(len(Vmodels))]


plot_dataset_fit(ts,Vexps,fourspin_dipolarsignal,params,scales,saveas='DEER_global_Q5_Vfit.svg')
```

```
[30-5-2022 16:58:56] Preparing the SNLLS analysis...
[30-5-2022 16:58:59] Preparations completed.
[30-5-2022 16:58:59] Non-linear least-squares routine in progress...
   Iteration     Total nfev        Cost      Cost reduction    Step norm     Optimality   
       0              1         1.3749e+05                                    1.30e+09    
       1              3         1.1147e+05      2.60e+04       1.66e-01       7.87e+06    
       2              4         1.4251e+04      9.72e+04       1.50e-01       2.24e+06    
       3              5         2.9497e+03      1.13e+04       1.58e-01       9.82e+06    
       4              7         2.8484e+03      1.01e+02       7.20e-02       1.30e+07    
       5              8         2.3725e+03      4.76e+02       3.44e-02       2.41e+05    
       6             10         2.3679e+03      4.65e+00       1.25e-02       4.99e+06    
       7             12         2.3509e+03      1.70e+01       1.30e-03       9.95e+05    
`ftol` termination condition is satisfied.
Function evaluations 12, initial cost 1.3749e+05, final cost 2.3509e+03, first-order optimality 9.95e+05.
[30-5-2022 17:24:12] Least-squares routine finished.
[30-5-2022 17:24:12] Uncertainty analysis in progress...
[30-5-2022 17:29:18] Uncertainty analysis completed.
[30-5-2022 17:29:18] Model evaluation in progress...
[30-5-2022 17:40:57] Model evaluation completed.
Goodness-of-fit: 
========= ============= ============ ======= =========== 
 Dataset   Noise level   Reduced 𝛘2   RMSD       AIC     
========= ============= ============ ======= =========== 
   #1         0.004        5.013      0.008   -1312.661  
   #2         0.004        3.527      0.008   -1369.426  
   #3         0.006        1.402      0.007   -1469.284  
   #4         0.004        6.440      0.009   -1303.175  
========= ============= ============ ======= =========== 
Model parameters: 
============ ======= ========================= ====== ======================================================= 
 Parameter    Value   95%-Confidence interval   Unit   Description                                            
============ ======= ========================= ====== ======================================================= 
 mean1        3.074   (2.661,3.487)              nm    Average inter-spin distance  #1                        
 mean2        3.793   (1.000,8.000)              nm    Average inter-spin distance  #2                        
 mean3        1.899   (1.762,2.037)              nm    Average inter-spin distance  #3                        
 mean4        2.006   (1.998,2.014)              nm    Average inter-spin distance  #4                        
 mean5        3.735   (3.714,3.755)              nm    Average inter-spin distance  #5                        
 mean6        3.347   (3.280,3.414)              nm    Average inter-spin distance  #6                        
 chol11       0.332   (0.100,0.692)              nm    Cholesky factor ℓ11                                    
 chol22       0.107   (0.100,0.800)              nm    Cholesky factor ℓ22                                    
 chol33       0.465   (0.305,0.625)              nm    Cholesky factor ℓ33                                    
 chol44       0.427   (0.417,0.437)              nm    Cholesky factor ℓ44                                    
 chol55       0.106   (0.100,0.125)              nm    Cholesky factor ℓ55                                    
 chol66       0.219   (0.176,0.262)              nm    Cholesky factor ℓ66                                    
 chol21       0.000   (frozen)                   nm    Cholesky factor ℓ21                                    
 chol31       0.000   (frozen)                   nm    Cholesky factor ℓ31                                    
 chol41       0.000   (frozen)                   nm    Cholesky factor ℓ41                                    
 chol51       0.000   (frozen)                   nm    Cholesky factor ℓ51                                    
 chol61       0.000   (frozen)                   nm    Cholesky factor ℓ61                                    
 chol32       0.000   (frozen)                   nm    Cholesky factor ℓ32                                    
 chol42       0.000   (frozen)                   nm    Cholesky factor ℓ42                                    
 chol52       0.000   (frozen)                   nm    Cholesky factor ℓ52                                    
 chol62       0.000   (frozen)                   nm    Cholesky factor ℓ62                                    
 chol43       0.000   (frozen)                   nm    Cholesky factor ℓ43                                    
 chol53       0.000   (frozen)                   nm    Cholesky factor ℓ53                                    
 chol63       0.000   (frozen)                   nm    Cholesky factor ℓ63                                    
 chol54       0.000   (frozen)                   nm    Cholesky factor ℓ54                                    
 chol64       0.000   (frozen)                  None   None                                                   
 chol65       0.000   (frozen)                   nm    Cholesky factor ℓ65                                    
 tau1         0.412   (0.411,0.413)              μs    First inter-pulse delay                                
 tau2         8.000   (frozen)                   μs    Second inter-pulse delay                               
 lamu_1       1.019   (0.000,10.000)            None   Amplitude of unmodulated pairwise pathway              
 lam1_1       0.001   (0.000,0.140)             None   Amplitude of pairwise pathway #1                       
 lam23        0.000   (frozen)                  None   Amplitude of pairwise pathways #2 and #3               
 conc_decay   0.360   (0.307,0.413)              μM    Spin concentration / Decay rate                        
 d            1.295   (1.255,1.336)              μM    Fractal dimension                                      
 lamu_2       1.000   (0.000,10.000)            None   Amplitude of unmodulated pairwise pathway              
 lam1_2       0.001   (0.000,0.118)             None   Amplitude of pairwise pathway #1                       
 lamu_3       0.994   (0.000,10.000)            None   Amplitude of unmodulated pairwise pathway              
 lam1_3       0.001   (0.000,0.102)             None   Amplitude of pairwise pathway #1                       
 lamu_4       0.983   (0.000,10.000)            None   Amplitude of unmodulated pairwise pathway              
 lam1_4       0.001   (0.000,0.094)             None   Amplitude of pairwise pathway #1                       
 scale_1      1.089   (1.088,1.090)             None   Echo amplitude in the abscense of dipolar modulations  
 scale_2      1.146   (1.145,1.147)             None   Echo amplitude in the abscense of dipolar modulations  
 scale_3      1.176   (1.175,1.177)             None   Echo amplitude in the abscense of dipolar modulations  
 scale_4      1.157   (1.154,1.159)             None   Echo amplitude in the abscense of dipolar modulations  
============ ======= ========================= ====== =======================================================
```

In [ ]:

```

```
