# Supplementary material for: Dipolar pathways in multi-spin and multi-dimensional dipolar EPR spectroscopy
Source: Phys Chem Chem Phys. 2022 Sep 8;24(37):22645–60. doi: 10.1039/d2cp03048a (PMC9516884; doi:10.1039/d2cp03048a)
Supplement: CP-024-D2CP03048A-s004 [file CP-024-D2CP03048A-s004.html]

analysis\_multispin\_TRIER


In [ ]:

```
import numpy as  np 
import matplotlib.pyplot as plt 
import deerlab as dl 
from multispin_analysis_module import *
from scipy.interpolate import interp2d
```

In [ ]:

```
def get_corrs(file):
    if 'txt' in file:
        HSCdata = np.loadtxt(file)
    if 'mat' in file:
        HSCdata = loadmat(file)['rlist']
    corrs = []
    for n1,n2 in [[0,1],[1,2],[2,0]]:
        hist,bins1,bins2 = np.histogram2d(HSCdata[:,n1],HSCdata[:,n2],bins=30)
        bins1 = bins1[:-1] + (bins1[1]-bins1[0])/2 
        bins2 = bins2[:-1] + (bins2[1]-bins2[0])/2
        xx, yy = np.meshgrid(bins1, bins2)
        grid2d = np.empty(xx.shape + (2,))
        grid2d[:, :, 0] = xx
        grid2d[:, :, 1] = yy
        
        def Pmodelflat(mean1,mean2,std1,std2,corr):
            L = np.atleast_2d([
                [std1,    0],
                [corr, std2]])
            P = Pbivar([mean1,mean2],L@L.T).pdf(grid2d)
            return P.flatten() 
        model = dl.Model(Pmodelflat)
        model.mean1.set(par0=3,lb=0,ub=8)
        model.mean2.set(par0=3,lb=0,ub=8)
        model.std1.set(par0=0.2,lb=0.01,ub=1)
        model.std2.set(par0=0.2,lb=0.01,ub=1) 
        model.corr.set(par0=0,lb=-0.5,ub=0.5)

        fit = dl.fit(model,hist.flatten())
        corrs.append(fit.corr) 
    return corrs
```

## Model (Three spins)¶

In [ ]:

```
#------------------------------------------------------------------------------
def threespin_dipolarsignal(t1,t2,means,cholfactors,tau1,tau2,tau3,
            lamu,lam1,lam2,lam3,lam4,lam5,lam6,lam7,lam8,lam9,lam10,lam11,lam12,lam13,lam14,lam15,lam16,lam17,lam18,lam19,lam20,lam21,lam22,lam23,lam24,
            conc_decay,d,threespin=False,fractal=False):

    tref1s,δ1s,tref2s,δ2s,λs= [],[],[],[],[]

    # Pairwise pathways modulated along t1
    reftimes_t1 = [tau1,  tau1+tau2, tau1-tau2,    0,  tau2,  tau2+tau3+tau1, -tau3, tau2+tau3]
    lams_t1     = [lam1,       lam3,      lam5, lam7,  lam9,           lam11, lam13,     lam15]

    # Pairwise pathways modulated along t2
    reftimes_t2 = [tau3, tau2+tau3,    0, tau3-tau1, tau2+tau3+tau1,  tau2, -tau1, tau1+tau2]
    lams_t2     = [lam2,      lam4, lam6,      lam8,          lam10, lam12, lam14,     lam16]

    # Pairwise pathways modulated along t2
    reftimes_t1t2 = [[-tau3,tau3], [tau2,tau2], [0, 0], [tau2+tau3+tau1,tau2+tau3+tau1], [tau2+tau3,tau2+tau3], [tau1,-tau1], [tau1+tau2,tau1+tau2], [tau1-tau3,tau3-tau1]]
    lams_t1t2     = [       lam17,       lam18,  lam19,                           lam20,                 lam21,        lam22,                 lam23,                 lam24]

    Npermutations = 6

    # Two-spin contributions
    for reftime,lam in zip(reftimes_t1,lams_t1): 
        # Pathway set 1 - two-spin, t1-modulated
        tref1, tref2 = [ reftime, None, None], [None, None, None]
        δ1, δ2       = [       1,    0,    0], [   0,    0,    0]
        tref1s, tref2s = [add_permutations(trefs,tref) for trefs,tref in zip([tref1s,tref2s],[tref1,tref2])]
        δ1s, δ2s = [add_permutations(δs,δ) for δs,δ in zip([δ1s,δ2s],[δ1,δ2])]
        λs += [lam*lamu*lamu]*Npermutations

    for reftime,lam in zip(reftimes_t2,lams_t2): 
        # Pathway set 1 - two-spin, t1-modulated
        tref1, tref2 = [ None, None, None], [ reftime, None, None]
        δ1, δ2       = [    0,    0,    0], [       1,    0,    0]
        tref1s, tref2s = [add_permutations(trefs,tref) for trefs,tref in zip([tref1s,tref2s],[tref1,tref2])]
        δ1s, δ2s = [add_permutations(δs,δ) for δs,δ in zip([δ1s,δ2s],[δ1,δ2])]
        λs += [lam*lamu*lamu]*Npermutations

    for reftime,lam in zip(reftimes_t1t2,lams_t1t2): 
        if lam>0.001:
            # Pathway set 1 - two-spin, t1-modulated
            tref1, tref2 = [ reftime[0], None, None], [ reftime[1], None, None]
            δ1, δ2       = [          1,    0,    0], [          1,    0,    0]
            tref1s, tref2s = [add_permutations(trefs,tref) for trefs,tref in zip([tref1s,tref2s],[tref1,tref2])]
            δ1s, δ2s = [add_permutations(δs,δ) for δs,δ in zip([δ1s,δ2s],[δ1,δ2])]
            λs += [lam*lamu*lamu]*Npermutations

    ### Three-spin contributions
    if threespin:
        for reftime1,lam1 in zip(reftimes_t1,lams_t1): 
            for reftime2,lam2 in zip(reftimes_t1,lams_t1): 
                if lam1*lam2*lamu>0.001:
                    # Pathway set 7 - three-spin, t1,t2-modulated
                    tref1, tref2 = [ reftime1, reftime2, None], [None, None, None]
                    δ1, δ2       = [        1,        1,    0], [   0,    0,    0]
                    tref1s, tref2s = [add_permutations(trefs,tref) for trefs,tref in zip([tref1s,tref2s],[tref1,tref2])]
                    δ1s, δ2s = [add_permutations(δs,δ) for δs,δ in zip([δ1s,δ2s],[δ1,δ2])]
                    λs += [lam1*lam2*lamu]*Npermutations
        for reftime1,lam1 in zip(reftimes_t2,lams_t2): 
            for reftime2,lam2 in zip(reftimes_t2,lams_t2): 
                if lam1*lam2*lamu>0.001:
                    # Pathway set 7 - three-spin, t1,t2-modulated
                    tref1, tref2 = [ None, None, None], [ reftime1, reftime2, None]
                    δ1, δ2       = [    0,    0,    0], [        1,        1,    0]
                    tref1s, tref2s = [add_permutations(trefs,tref) for trefs,tref in zip([tref1s,tref2s],[tref1,tref2])]
                    δ1s, δ2s = [add_permutations(δs,δ) for δs,δ in zip([δ1s,δ2s],[δ1,δ2])]
                    λs += [lam1*lam2*lamu]*Npermutations
        for reftime1,lam1 in zip(reftimes_t1,lams_t1): 
            for reftime2,lam2 in zip(reftimes_t2,lams_t2): 
                if lam1*lam2*lamu>0.001:
                    # Pathway set 7 - three-spin, t1,t2-modulated
                    tref1, tref2 = [ reftime1, None, None], [ None, reftime2, None]
                    δ1, δ2       = [        1,    0,    0], [    0,        1,    0]
                    tref1s, tref2s = [add_permutations(trefs,tref) for trefs,tref in zip([tref1s,tref2s],[tref1,tref2])]
                    δ1s, δ2s = [add_permutations(δs,δ) for δs,δ in zip([δ1s,δ2s],[δ1,δ2])]
                    λs += [lam1*lam2*lamu]*Npermutations

    # Monte-Carlo multivariate distance integration
    Nsamples = 500000
    np.random.seed(seed=1)
    rsamples = Pmultivar(means,cholfactors).rvs(Nsamples)
    rsamples = np.maximum(rsamples,1e-16) # Avoid values exactly at zero 

    # Triangle inequalities
    Ndistances = np.shape(rsamples)[1]
    idx = np.arange(Ndistances)
    triangle_condition = np.full_like(rsamples,False)
    for n in range(Ndistances):
        idx = np.roll(idx,-1,axis=0)
        triangle_condition[:,n] = np.sum(rsamples[:,idx[:-1]],axis=1) > rsamples[:,idx[-1]]
    triangle_condition = np.all(triangle_condition,axis=1)
    # Discard samples that do not satisfy triangle inequalities
    rsamples = rsamples[triangle_condition,:]

    # Construct the dipolar signal 
    λ0 = np.maximum(1 - np.sum(λs),0)
    Vintra,Vinter = λ0,1
    twospin_contributions,threespin_contributions = [],[]
    # Loop over all dipolar pathways
    for δ1,tref1,δ2,tref2,λ in zip(δ1s,tref1s,δ2s,tref2s,λs):
        
        # Set trefs defined as None to an arbitrary numerical value
        tref1,tref2 = [[tref[n] if δn!=0 else 0 for n,δn in enumerate(δ)] for tref,δ in zip([tref1,tref2],[δ1,δ2])]

        # Number of spins participating in the pathway
        Nspin = np.sum(δ1)+np.sum(δ2)+1 
                    
        # Two-spin intramolecular contributions
        if Nspin==2:
            Vintra_contr = 0
            if np.sum(δ1)>0:
                n = int(np.where(δ1==1)[0])

                # Estimate the 1D-marginal distance distribution
                Pmarginal,bins = np.histogram(rsamples[:,n], bins=300)
                rgrid = (bins[:-1] + bins[1:])/2
                Pmarginal = Pmarginal/np.trapz(Pmarginal,rgrid)

                # Two-spin intramolecular contribution modulated along t1-dimension
                Vintra_contr_ = dl.dipolarkernel(δ1[n]*(t1-tref1[n]),rgrid)@Pmarginal
                Vintra_contr += np.repeat(Vintra_contr_[:,np.newaxis] ,len(t2),axis=1)

            if np.sum(δ2)>0:
                n = int(np.where(δ2==1)[0])

                # Estimate the 1D-marginal distance distribution
                Pmarginal,bins = np.histogram(rsamples[:,n], bins=300)
                rgrid = (bins[:-1] + bins[1:])/2
                Pmarginal = Pmarginal/np.trapz(Pmarginal,rgrid)

                # Two-spin intramolecular contribution modulated along t2-dimension
                Vintra_contr_ = dl.dipolarkernel(δ2[n]*(t2-tref2[n]),rgrid)@Pmarginal
                Vintra_contr += np.repeat(Vintra_contr_[np.newaxis,:] ,len(t1),axis=0)
                
            twospin_contributions.append(λ*Vintra_contr)


        # Three-spin intramolecular contributions
        if Nspin==3:

            # Non-uniformly subsample the time-domain (speedup)
            n1 = int(np.where(δ1==1)[0][0]) if np.sum(δ1)>0 else 0
            n2 = int(np.where(δ2==1)[0][0]) if np.sum(δ2)>0 else 0
            np.random.seed(0)
            subsampling1 = np.exp(-6*abs(t1-tref1[n1])/max(t1))*np.random.rand(len(t1))>0.025
            subsampling2 = np.exp(-6*abs(t2-tref2[n2])/max(t2))*np.random.rand(len(t2))>0.025
            subsampling1[-1] = 1
            subsampling2[-1] = 1
            t1sub = t1[subsampling1]
            t2sub = t2[subsampling2]

            # Average three-spin contribution over all distances
            Nsamples = 100
            Vintra_contr = 0
            for rs in rsamples[:Nsamples,:]: 
                r1,r2,r3 = rs
                # Three-spin intramolecular contribution
                Vintra_contr += threespin_dipolarkernel2D(t1sub,t2sub,r1,r2,r3,δ1,tref1,δ2,tref2) 
            Vintra_contr /= Nsamples
            Vintra_contr = interp2d(t1sub,t2sub,Vintra_contr.T)(t1,t2).T
            threespin_contributions.append(λ*Vintra_contr)

        # Incorporate intermolecular contribution
        Vintra += λ*Vintra_contr

        # Intermolecular contribution
        Vinter *= Bbasis2D(t1,t2,λ,conc_decay,d,δ1,δ2,tref1,tref2,fractal=fractal)

    Vinter = Vinter/np.max(Vinter)
    
    # Construct the total dipolar signal
    V = Vintra*Vinter
    V = np.maximum(V,1e-16)

    return V,λ0,Vinter,Vintra,twospin_contributions,threespin_contributions
#------------------------------------------------------------------------------

#------------------------------------------------------------------------------
def construct_threespin_dipolarmodel(t1,t2,tau1_exp,tau2_exp,tau3_exp,threespin=False,fractal=False):

    def _threespin_dipolarsignal(mean1,mean2,mean3,chol11,chol22,chol33,chol21,chol31,chol32,tau1,tau2,tau3,
                            lamu,lam1,lam2,lam3,lam4,lam5,lam6,lam7,lam8,lam9,lam10,lam11,lam12,lam13,lam14,lam15,
                        lam16,lam17,lam18,lam19,lam20,lam21,lam22,lam23,lam24,conc_decay,d):
            Vfit = threespin_dipolarsignal(t1,t2,[mean1,mean2,mean3],[chol11,chol22,chol33,chol21,chol31,chol32],tau1,tau2,tau3,
                                    lamu,lam1,lam2,lam3,lam4,lam5,lam6,lam7,lam8,lam9,lam10,lam11,lam12,lam13,lam14,lam15,
                            lam16,lam17,lam18,lam19,lam20,lam21,lam22,lam23,lam24,conc_decay,d,threespin=threespin, fractal=fractal)[0]
            Vfit = np.atleast_2d(Vfit.flatten()).T # Flatten to 1D-vector
            return Vfit 
    # Construct model 
    Vmultispin = dl.Model(_threespin_dipolarsignal)
    Vmultispin.description = 'Multi-spin dipolar signal arising from a three-spin TRIER experiment'
    # Set parameter properties
    Vmultispin.addlinear('scale',lb=0, description='Echo amplitude in the abscense of dipolar modulations')
    Vmultispin.mean1.set(lb=1.0, ub=8.0, par0=3.0, unit='nm', description='Average inter-spin distance  #1')
    Vmultispin.mean2.set(lb=1.0, ub=8.0, par0=3.0, unit='nm', description='Average inter-spin distance  #2')
    Vmultispin.mean3.set(lb=1.0, ub=8.0, par0=3.0, unit='nm', description='Average inter-spin distance  #3')
    Vmultispin.chol11.set(lb=0.0, ub=0.8, par0=0.40, unit='nm', description='Cholesky factor ℓ11' )
    Vmultispin.chol22.set(lb=0.0, ub=0.8, par0=0.40, unit='nm', description='Cholesky factor ℓ22' )
    Vmultispin.chol33.set(lb=0.0, ub=0.8, par0=0.40, unit='nm', description='Cholesky factor ℓ33' )
    Vmultispin.chol21.set(lb=-1.0, ub=1.0, par0=0.0, unit='nm', description='Cholesky factor ℓ21' )
    Vmultispin.chol31.set(lb=-1.0, ub=1.0, par0=0.0, unit='nm', description='Cholesky factor ℓ31' )
    Vmultispin.chol32.set(lb=-1.0, ub=1.0, par0=0.0, unit='nm', description='Cholesky factor ℓ32' )
    Vmultispin.tau1.set(lb=tau1_exp-0.2, ub=tau1_exp+0.2, par0=tau1_exp, unit='μs', description='First inter-pulse delay' )
    Vmultispin.tau2.set(lb=tau2_exp-0.2, ub=tau2_exp+0.2, par0=tau2_exp, unit='μs', description='Second inter-pulse delay' )
    Vmultispin.tau3.set(lb=tau3_exp-0.2, ub=tau3_exp+0.2, par0=tau3_exp, unit='μs', description='Third inter-pulse delay' )
    Vmultispin.lamu.set(lb=0.00, ub=2.00, par0=1.00, unit=None, description='Amplitude of unmodulated pairwise pathway' )
    Vmultispin.lam1.set(lb=0.00, ub=1.00, par0=0.02, unit=None, description='Dipolar pathway #1 probability' )
    Vmultispin.lam2.set(lb=0.00, ub=1.00, par0=0.02, unit=None, description='Dipolar pathway #2 probability' )
    Vmultispin.lam3.set(lb=0.00, ub=1.00, par0=0.00, unit=None, description='Dipolar pathway #3 probability' )
    Vmultispin.lam4.set(lb=0.00, ub=1.00, par0=0.00, unit=None, description='Dipolar pathway #4 probability' )
    Vmultispin.lam5.set(lb=0.00, ub=1.00, par0=0.00, unit=None, description='Dipolar pathway #5 probability' )
    Vmultispin.lam6.set(lb=0.00, ub=1.00, par0=0.00, unit=None, description='Dipolar pathway #6 probability' )
    Vmultispin.lam7.set(lb=0.00, ub=1.00, par0=0.00, unit=None, description='Dipolar pathway #7 probability' )
    Vmultispin.lam8.set(lb=0.00, ub=1.00, par0=0.00, unit=None, description='Dipolar pathway #8 probability' )
    Vmultispin.lam9.set(lb=0.00, ub=1.00, par0=0.00, unit=None, description='Dipolar pathway #9 probability' )
    Vmultispin.lam10.set(lb=0.00, ub=1.00, par0=0.00, unit=None, description='Dipolar pathway #10 probability' )
    Vmultispin.lam11.set(lb=0.00, ub=1.00, par0=0.00, unit=None, description='Dipolar pathway #11 probability' )
    Vmultispin.lam12.set(lb=0.00, ub=1.00, par0=0.00, unit=None, description='Dipolar pathway #12 probability' )
    Vmultispin.lam13.set(lb=0.00, ub=1.00, par0=0.00, unit=None, description='Dipolar pathway #13 probability' )
    Vmultispin.lam14.set(lb=0.00, ub=1.00, par0=0.00, unit=None, description='Dipolar pathway #14 probability' )
    Vmultispin.lam15.set(lb=0.00, ub=1.00, par0=0.00, unit=None, description='Dipolar pathway #15 probability' )
    Vmultispin.lam16.set(lb=0.00, ub=1.00, par0=0.00, unit=None, description='Dipolar pathway #16 probability' )
    Vmultispin.lam17.set(lb=0.00, ub=1.00, par0=0.00, unit=None, description='Dipolar pathway #17 probability' )
    Vmultispin.lam18.set(lb=0.00, ub=1.00, par0=0.00, unit=None, description='Dipolar pathway #18 probability' )
    Vmultispin.lam19.set(lb=0.00, ub=1.00, par0=0.00, unit=None, description='Dipolar pathway #19 probability' )
    Vmultispin.lam20.set(lb=0.00, ub=1.00, par0=0.00, unit=None, description='Dipolar pathway #20 probability' )
    Vmultispin.lam21.set(lb=0.00, ub=1.00, par0=0.00, unit=None, description='Dipolar pathway #21 probability' )
    Vmultispin.lam22.set(lb=0.00, ub=1.00, par0=0.00, unit=None, description='Dipolar pathway #22 probability' )
    Vmultispin.lam23.set(lb=0.00, ub=1.00, par0=0.00, unit=None, description='Dipolar pathway #23 probability' )
    Vmultispin.lam24.set(lb=0.00, ub=1.00, par0=0.00, unit=None, description='Dipolar pathway #24 probability' )
    Vmultispin.conc_decay.set(lb=0, ub=50, par0=0.005, unit='μM', description='Spin concentration / Decay rate' )
    Vmultispin.d.set(lb=1, ub=3, par0=1.20, unit='μM', description='Stretch factor' )
    if not fractal:
        Vmultispin.d.freeze(1)
        Vmultispin.conc_decay.set(lb=0, ub=500, par0=40, unit='μM', description='Spin concentration / Decay rate' )
    else:
        Vmultispin.conc_decay.set(lb=0, ub=5, par0=0.05, unit='μM', description='Spin concentration / Decay rate' )

    return Vmultispin
#------------------------------------------------------------------------------
```

---

## Triradical T111¶

---

In [ ]:

```
threespin = True 
fractal = True

# Load experimental data
data = loadmat(r'.\\data\\triradical_T111_TRIER.mat')
HSCfile=r'.\\HSC_simulations\\HSCsim_T111_100000samples.txt'
t1 = np.squeeze(data['t1'])/1000 # ns -> μs
t2 = np.squeeze(data['t2'])/1000 # ns -> μs
Vexp = np.squeeze(data['V2d']).T
tau1_exp = np.squeeze(data['tau1'])/1000 # ns -> μs
tau2_exp = np.squeeze(data['tau2'])/1000 # ns -> μs
tau3_exp = np.squeeze(data['tau3'])/1000 # ns -> μs
Vexp /= np.max(Vexp) 

# Subsample the signal due to limited memory
subsampling1 = np.arange(0,len(t1),3)
subsampling2 = np.arange(0,len(t2),3)
t1_sub = t1[subsampling1]
t2_sub = t2[subsampling2]
Vexp_sub = Vexp[np.ix_(subsampling1,subsampling2)]

# Construct the three-spin TRIER model
Vmodel_TRIER = construct_threespin_dipolarmodel(t1_sub,t2_sub,tau1_exp,tau2_exp,tau3_exp,threespin=threespin,fractal=fractal)

# Use the HSC simulation to get a good starting point
HSCdata = np.loadtxt(HSCfile)
for n in range(HSCdata.shape[1]):
    hist,bins = np.histogram(HSCdata[:,n],bins=200)
    bins = bins[:-1] + (bins[1]-bins[0])/2 
    fit = dl.fit(dl.dd_gauss,hist,bins)
    getattr(Vmodel_TRIER,f'mean{n+1}').par0 = fit.mean
    getattr(Vmodel_TRIER,f'chol{n+1}{n+1}').par0 = fit.std
corrs = get_corrs(HSCfile)
Vmodel_TRIER.chol21.par0 = corrs[0]
Vmodel_TRIER.chol31.par0 = corrs[1]
Vmodel_TRIER.chol32.par0 = corrs[2]

# Ignore pathways refocusing outside of the detected signal
Vmodel_TRIER.lam5.freeze(0)
Vmodel_TRIER.lam6.freeze(0)
Vmodel_TRIER.lam7.freeze(0)
Vmodel_TRIER.lam8.freeze(0)
Vmodel_TRIER.lam13.freeze(0)
Vmodel_TRIER.lam14.freeze(0)
Vmodel_TRIER.lam17.freeze(0)
Vmodel_TRIER.lam18.freeze(0)
Vmodel_TRIER.lam19.freeze(0)
Vmodel_TRIER.lam20.freeze(0)
Vmodel_TRIER.lam21.freeze(0)
Vmodel_TRIER.lam22.freeze(0)
Vmodel_TRIER.lam23.freeze(0)
Vmodel_TRIER.lam24.freeze(0)

# Fit the model to the data 
results = dl.fit(Vmodel_TRIER, Vexp_sub.flatten(), reg=False, ftol=1e-3, max_nfev=400,verbose=2) 

# Print the fit summary 
print(results)

# Plot the multivariate distance distribution
means = [results.mean1, results.mean2, results.mean3]
cholesky_factors = [results.chol11,results.chol22,results.chol33,results.chol21, results.chol31, results.chol32] 
plot_multivariate(means,cholesky_factors,xlim=[0,8],saveas='TRIER_T111_Pfit.svg',HSCfile=HSCfile)

# Plot the fitted TRIER signal
params = [t1,t2,means,cholesky_factors,results.tau1,results.tau2,results.tau3,results.lamu] + [getattr(results,f'lam{n+1}') for n in range(24)] + [results.conc_decay,results.d,threespin,fractal]
plot_trier_fit(t1,t2,Vexp,threespin_dipolarsignal,params,results.scale,saveas='TRIER_T111_Vfit.svg',levels=60)
```

```
[30-5-2022 18:33:41] Preparing the SNLLS analysis...
[30-5-2022 18:33:42] Preparations completed.
[30-5-2022 18:33:42] Non-linear least-squares routine in progress...
   Iteration     Total nfev        Cost      Cost reduction    Step norm     Optimality   
       0              1         2.9169e+00                                    6.77e+01    
       1              3         1.6672e+00      1.25e+00       1.38e-02       6.92e+01    
       2              5         1.0079e+00      6.59e-01       5.85e-03       6.62e+01    
       3              6         4.3092e-01      5.77e-01       1.18e-02       9.86e+00    
       4              7         3.4385e-01      8.71e-02       4.23e-02       1.17e+01    
       5              8         2.3168e-01      1.12e-01       8.99e-02       1.05e+01    
       6              9         1.6744e-01      6.42e-02       8.90e-02       9.13e+00    
       7             10         8.1512e-02      8.59e-02       1.96e-01       1.62e+01    
       8             11         7.3916e-02      7.60e-03       4.48e-02       3.66e+00    
       9             12         6.7411e-02      6.51e-03       4.50e-02       5.93e+00    
      10             13         5.6784e-02      1.06e-02       1.43e-01       2.81e+00    
      11             16         5.6783e-02      1.02e-06       1.61e-05       5.02e+00    
      12             17         5.6781e-02      1.32e-06       6.08e-06       1.27e+00    
      13             19         5.6781e-02      1.32e-08       3.21e-07       1.78e+00    
      14             20         5.6781e-02      3.82e-07       7.33e-08       4.14e+00    
Both `ftol` and `xtol` termination conditions are satisfied.
Function evaluations 20, initial cost 2.9169e+00, final cost 5.6781e-02, first-order optimality 4.14e+00.
[30-5-2022 19:19:8] Least-squares routine finished.
[30-5-2022 19:19:8] Uncertainty analysis in progress...
[30-5-2022 19:24:0] Uncertainty analysis completed.
[30-5-2022 19:24:0] Model evaluation in progress...
[30-5-2022 19:26:37] Model evaluation completed.
Goodness-of-fit: 
========= ============= ============ ======= =========== 
 Dataset   Noise level   Reduced 𝛘2   RMSD       AIC     
========= ============= ============ ======= =========== 
   #1         0.003        8.329      0.009   -3386.798  
========= ============= ============ ======= =========== 
Model parameters: 
============ ======== ========================= ====== ======================================================= 
 Parameter    Value    95%-Confidence interval   Unit   Description                                            
============ ======== ========================= ====== ======================================================= 
 mean1        3.570    (2.910,4.230)              nm    Average inter-spin distance  #1                        
 mean2        3.610    (2.853,4.366)              nm    Average inter-spin distance  #2                        
 mean3        3.623    (2.904,4.342)              nm    Average inter-spin distance  #3                        
 chol11       0.463    (0.462,0.463)              nm    Cholesky factor ℓ11                                    
 chol22       0.459    (0.459,0.459)              nm    Cholesky factor ℓ22                                    
 chol33       0.459    (0.459,0.459)              nm    Cholesky factor ℓ33                                    
 chol21       0.001    (0.001,0.002)              nm    Cholesky factor ℓ21                                    
 chol31       -0.001   (-0.001,-0.000)            nm    Cholesky factor ℓ31                                    
 chol32       -0.001   (-0.001,-0.001)            nm    Cholesky factor ℓ32                                    
 tau1         0.428    (0.425,0.430)              μs    First inter-pulse delay                                
 tau2         4.557    (4.400,4.800)              μs    Second inter-pulse delay                               
 tau3         0.911    (0.909,0.914)              μs    Third inter-pulse delay                                
 lamu         0.981    (0.216,1.746)             None   Amplitude of unmodulated pairwise pathway              
 lam1         0.034    (0.000,0.085)             None   Dipolar pathway #1 probability                         
 lam2         0.048    (0.000,0.116)             None   Dipolar pathway #2 probability                         
 lam3         0.001    (0.000,0.006)             None   Dipolar pathway #3 probability                         
 lam4         0.000    (0.000,0.002)             None   Dipolar pathway #4 probability                         
 lam5         0.000    (frozen)                  None   Dipolar pathway #5 probability                         
 lam6         0.000    (frozen)                  None   Dipolar pathway #6 probability                         
 lam7         0.000    (frozen)                  None   Dipolar pathway #7 probability                         
 lam8         0.000    (frozen)                  None   Dipolar pathway #8 probability                         
 lam9         0.000    (0.000,0.002)             None   Dipolar pathway #9 probability                         
 lam10        0.001    (0.000,0.014)             None   Dipolar pathway #10 probability                        
 lam11        0.001    (0.000,0.028)             None   Dipolar pathway #11 probability                        
 lam12        0.000    (0.000,0.001)             None   Dipolar pathway #12 probability                        
 lam13        0.000    (frozen)                  None   Dipolar pathway #13 probability                        
 lam14        0.000    (frozen)                  None   Dipolar pathway #14 probability                        
 lam15        0.002    (0.000,0.023)             None   Dipolar pathway #15 probability                        
 lam16        0.000    (0.000,0.001)             None   Dipolar pathway #16 probability                        
 lam17        0.000    (frozen)                  None   Dipolar pathway #17 probability                        
 lam18        0.000    (frozen)                  None   Dipolar pathway #18 probability                        
 lam19        0.000    (frozen)                  None   Dipolar pathway #19 probability                        
 lam20        0.000    (frozen)                  None   Dipolar pathway #20 probability                        
 lam21        0.000    (frozen)                  None   Dipolar pathway #21 probability                        
 lam22        0.000    (frozen)                  None   Dipolar pathway #22 probability                        
 lam23        0.000    (frozen)                  None   Dipolar pathway #23 probability                        
 lam24        0.000    (frozen)                  None   Dipolar pathway #24 probability                        
 conc_decay   0.139    (0.050,0.228)              μM    Spin concentration / Decay rate                        
 d            1.110    (1.000,1.344)              μM    Stretch factor                                         
 scale        1.000    (1.000,1.000)             None   Echo amplitude in the abscense of dipolar modulations  
============ ======== ========================= ====== =======================================================
```

---

## Triradical T011¶

---

In [ ]:

```
threespin = True 
fractal = True

# Load experimental data
data = loadmat(r'.\\data\\triradical_T011_TRIER.mat')
HSCfile=r'.\\HSC_simulations\\HSCsim_triradical_T011_100000samples.txt'
t1 = np.squeeze(data['t1'])/1000 # ns -> μs
t2 = np.squeeze(data['t2'])/1000 # ns -> μs
Vexp = np.squeeze(data['V2d']).T
tau1_exp = np.squeeze(data['tau1'])/1000 # ns -> μs
tau2_exp = np.squeeze(data['tau2'])/1000 # ns -> μs
tau3_exp = np.squeeze(data['tau3'])/1000 # ns -> μs
Vexp /= np.max(Vexp) 

# Subsample the signal due to limited memory
subsampling1 = np.arange(0,len(t1),2)
subsampling2 = np.arange(0,len(t2),2)
t1_sub = t1[subsampling1]
t2_sub = t2[subsampling2]
Vexp_sub = Vexp[np.ix_(subsampling1,subsampling2)]

# Construct the three-spin TRIER model
Vmodel_TRIER = construct_threespin_dipolarmodel(t1_sub,t2_sub,tau1_exp,tau2_exp,tau3_exp,threespin=threespin,fractal=fractal)

# Use the HSC simulation to get a good starting point
HSCdata = np.loadtxt(HSCfile)
for n in range(HSCdata.shape[1]):
    hist,bins = np.histogram(HSCdata[:,n],bins=200)
    bins = bins[:-1] + (bins[1]-bins[0])/2 
    fit = dl.fit(dl.dd_gauss,hist,bins)
    getattr(Vmodel_TRIER,f'mean{n+1}').par0 = fit.mean
    getattr(Vmodel_TRIER,f'chol{n+1}{n+1}').par0 = fit.std
corrs = get_corrs(HSCfile)
Vmodel_TRIER.chol21.par0 = corrs[0]
Vmodel_TRIER.chol31.par0 = corrs[1]
Vmodel_TRIER.chol32.par0 = corrs[2]

# Ignore pathways refocusing outside of the detected signal
Vmodel_TRIER.lam5.freeze(0)
Vmodel_TRIER.lam6.freeze(0)
Vmodel_TRIER.lam7.freeze(0)
Vmodel_TRIER.lam8.freeze(0)
Vmodel_TRIER.lam13.freeze(0)
Vmodel_TRIER.lam14.freeze(0)
# Ignore two-dimensionally modulated pathways 
Vmodel_TRIER.lam17.freeze(0)
Vmodel_TRIER.lam18.freeze(0)
Vmodel_TRIER.lam19.freeze(0)
Vmodel_TRIER.lam20.freeze(0)
Vmodel_TRIER.lam21.freeze(0)
Vmodel_TRIER.lam22.freeze(0)
Vmodel_TRIER.lam23.freeze(0)
Vmodel_TRIER.lam24.freeze(0)

# Fit the model to the data 
results = dl.fit(Vmodel_TRIER, Vexp_sub.flatten(), reg=False, ftol=1e-4, max_nfev=350) 

# Print the fit summary 
print(results)

# Plot the multivariate distance distribution
means = [results.mean1, results.mean2, results.mean3]
cholesky_factors = [results.chol11,results.chol22,results.chol33,results.chol21, results.chol31, results.chol32] 
plot_multivariate(means,cholesky_factors,xlim=[0,8],saveas='TRIER_T011_Pfit.svg', HSCfile=HSCfile)

# Plot the fitted TRIER signal
params = [t1,t2,means,cholesky_factors,results.tau1,results.tau2,results.tau3, results.lamu] + [getattr(results,f'lam{n+1}') for n in range(24)] + [results.conc_decay,results.d,threespin,fractal]
plot_trier_fit(t1,t2,Vexp,threespin_dipolarsignal,params,results.scale,saveas='TRIER_T011_Vfit.svg',levels=45)
```

```
Goodness-of-fit: 
========= ============= ============ ======= =========== 
 Dataset   Noise level   Reduced 𝛘2   RMSD       AIC     
========= ============= ============ ======= =========== 
   #1         0.004        3.420      0.007   -3604.884  
========= ============= ============ ======= =========== 
Model parameters: 
============ ======== ========================= ====== ======================================================= 
 Parameter    Value    95%-Confidence interval   Unit   Description                                            
============ ======== ========================= ====== ======================================================= 
 mean1        3.178    (2.436,3.921)              nm    Average inter-spin distance  #1                        
 mean2        3.170    (2.131,4.209)              nm    Average inter-spin distance  #2                        
 mean3        3.167    (1.997,4.338)              nm    Average inter-spin distance  #3                        
 chol11       0.465    (0.464,0.467)              nm    Cholesky factor ℓ11                                    
 chol22       0.435    (0.434,0.437)              nm    Cholesky factor ℓ22                                    
 chol33       0.441    (0.440,0.442)              nm    Cholesky factor ℓ33                                    
 chol21       0.027    (0.027,0.028)              nm    Cholesky factor ℓ21                                    
 chol31       -0.024   (-0.025,-0.024)            nm    Cholesky factor ℓ31                                    
 chol32       0.050    (-0.399,0.498)             nm    Cholesky factor ℓ32                                    
 tau1         0.387    (0.384,0.391)              μs    First inter-pulse delay                                
 tau2         2.778    (2.701,2.856)              μs    Second inter-pulse delay                               
 tau3         0.947    (0.945,0.949)              μs    Third inter-pulse delay                                
 lamu         0.885    (0.416,1.354)             None   Amplitude of unmodulated pairwise pathway              
 lam1         0.033    (0.002,0.064)             None   Dipolar pathway #1 probability                         
 lam2         0.035    (0.003,0.067)             None   Dipolar pathway #2 probability                         
 lam3         0.001    (0.000,0.027)             None   Dipolar pathway #3 probability                         
 lam4         0.000    (0.000,0.004)             None   Dipolar pathway #4 probability                         
 lam5         0.000    (frozen)                  None   Dipolar pathway #5 probability                         
 lam6         0.000    (frozen)                  None   Dipolar pathway #6 probability                         
 lam7         0.000    (frozen)                  None   Dipolar pathway #7 probability                         
 lam8         0.000    (frozen)                  None   Dipolar pathway #8 probability                         
 lam9         0.000    (0.000,0.002)             None   Dipolar pathway #9 probability                         
 lam10        0.006    (0.000,0.027)             None   Dipolar pathway #10 probability                        
 lam11        0.002    (0.000,0.180)             None   Dipolar pathway #11 probability                        
 lam12        0.000    (0.000,0.001)             None   Dipolar pathway #12 probability                        
 lam13        0.000    (frozen)                  None   Dipolar pathway #13 probability                        
 lam14        0.000    (frozen)                  None   Dipolar pathway #14 probability                        
 lam15        0.003    (0.000,0.156)             None   Dipolar pathway #15 probability                        
 lam16        0.001    (0.000,0.001)             None   Dipolar pathway #16 probability                        
 lam17        0.000    (frozen)                  None   Dipolar pathway #17 probability                        
 lam18        0.000    (frozen)                  None   Dipolar pathway #18 probability                        
 lam19        0.000    (frozen)                  None   Dipolar pathway #19 probability                        
 lam20        0.000    (frozen)                  None   Dipolar pathway #20 probability                        
 lam21        0.000    (frozen)                  None   Dipolar pathway #21 probability                        
 lam22        0.000    (frozen)                  None   Dipolar pathway #22 probability                        
 lam23        0.000    (frozen)                  None   Dipolar pathway #23 probability                        
 lam24        0.000    (frozen)                  None   Dipolar pathway #24 probability                        
 conc_decay   0.593    (0.077,1.109)              μM    Spin concentration / Decay rate                        
 d            1.112    (1.000,1.322)              μM    Stretch factor                                         
 scale        1.049    (1.049,1.049)             None   Echo amplitude in the abscense of dipolar modulations  
============ ======== ========================= ====== =======================================================
```

---

## Triradical Rpo47¶

---

In [ ]:

```
threespin = True 
fractal = False

# Load experimental data
data = loadmat('.\\data\\triradical_Rpo47_TRIER.mat')
MMMfile = r".\\MMMx_simulations\\MMMx_Rpo47_triple_labelled_sampled.mat"
t1 = np.squeeze(data['t1'])/1000 # ns -> μs
t2 = np.squeeze(data['t2'])/1000 # ns -> μs
Vexp = np.squeeze(data['V2d']).T
tau1_exp = np.squeeze(data['tau1'])/1000 # ns -> μs
tau2_exp = np.squeeze(data['tau2'])/1000 # ns -> μs
tau3_exp = np.squeeze(data['tau3'])/1000 # ns -> μs
Vexp /= np.max(Vexp) 

# Subsample the signal due to limited memory
subsampling1 = np.arange(0,len(t1),2)
subsampling2 = np.arange(0,len(t2),2)
t1_sub = t1[subsampling1]
t2_sub = t2[subsampling2]
Vexp_sub = Vexp[np.ix_(subsampling1,subsampling2)]

# Construct the three-spin TRIER model
Vmodel_TRIER = construct_threespin_dipolarmodel(t1_sub,t2_sub,tau1_exp,tau2_exp,tau3_exp,threespin=threespin,fractal=fractal)

# Use the HSC simulation to get a good starting point
from scipy.io import loadmat
MMMdata = loadmat(MMMfile)['rlist']
for n in range(MMMdata.shape[1]):
    hist,bins = np.histogram(MMMdata[:,n],bins=200)
    bins = bins[:-1] + (bins[1]-bins[0])/2 
    fit = dl.fit(dl.dd_gauss,hist,bins)
    getattr(Vmodel_TRIER,f'mean{n+1}').par0 = fit.mean
    getattr(Vmodel_TRIER,f'chol{n+1}{n+1}').par0 = fit.std
corrs = get_corrs(MMMfile)
Vmodel_TRIER.chol21.par0 = corrs[0]
Vmodel_TRIER.chol31.par0 = corrs[1]
Vmodel_TRIER.chol32.par0 = corrs[2]

# Ignore pathways refocusing outside of the detected signal
Vmodel_TRIER.lam5.freeze(0)
Vmodel_TRIER.lam6.freeze(0)
Vmodel_TRIER.lam7.freeze(0)
Vmodel_TRIER.lam8.freeze(0)
Vmodel_TRIER.lam13.freeze(0)
Vmodel_TRIER.lam14.freeze(0)

Vmodel_TRIER.lam17.freeze(0)
Vmodel_TRIER.lam18.freeze(0)
Vmodel_TRIER.lam19.freeze(0)
Vmodel_TRIER.lam20.freeze(0)
Vmodel_TRIER.lam21.freeze(0)
Vmodel_TRIER.lam22.freeze(0)
Vmodel_TRIER.lam23.freeze(0)
Vmodel_TRIER.lam24.freeze(0)

# Fit the model to the data 
results = dl.fit(Vmodel_TRIER, Vexp_sub.flatten(), reg=False, ftol=1e-4, max_nfev=300, verbose=2) 

# Print the fit summary 
print(results)

# Plot the multivariate distance distribution
means = [results.mean1, results.mean2, results.mean3]
cholesky_factors = [results.chol11,results.chol22,results.chol33,results.chol21, results.chol31, results.chol32] 
plot_multivariate(means,cholesky_factors,xlim=[0,8],saveas='TRIER_Rpo47_Pfit.svg',
        MMMfile = r"D:\lufa\projects\Multipathway  3spins\rpo47_triple_labelled_MMMx_sampled.mat")

# Plot the fitted TRIER signal
params = [t1,t2,means,cholesky_factors,results.tau1,results.tau2,results.tau3,results.lamu] + [getattr(results,f'lam{n+1}') for n in range(24)] + [results.conc_decay,results.d,threespin,fractal]
plot_trier_fit(t1,t2,Vexp,threespin_dipolarsignal,params,results.scale,saveas='TRIER_Rpo47_Vfit.svg',levels=40)
```

```
d:\lufa\projects\deerlab\deerlab\deerlab\utils\gof.py:53: RuntimeWarning: divide by zero encountered in double_scalars
  chi2red = 1/Ndof*np.linalg.norm(x - xfit)**2/sigma**2
```

```
[31-5-2022 12:48:45] Preparing the SNLLS analysis...
[31-5-2022 12:48:46] Preparations completed.
[31-5-2022 12:48:46] Non-linear least-squares routine in progress...
   Iteration     Total nfev        Cost      Cost reduction    Step norm     Optimality   
       0              1         3.9212e+00                                    1.70e+02    
       1              3         2.3134e+00      1.61e+00       7.56e-03       1.54e+02    
       2              4         5.8892e-01      1.72e+00       1.51e-02       9.54e+00    
       3              5         4.2989e-01      1.59e-01       2.57e-02       7.21e-01    
       4              6         3.8514e-01      4.47e-02       6.28e-02       4.86e+00    
       5              7         3.5830e-01      2.68e-02       2.69e-02       1.30e+00    
       6              8         3.4291e-01      1.54e-02       8.21e-02       8.86e+00    
       7              9         3.2940e-01      1.35e-02       1.53e-02       1.60e+00    
       8             10         3.0958e-01      1.98e-02       1.03e-01       5.53e+00    
       9             11         3.0115e-01      8.43e-03       5.58e-03       3.97e+00    
      10             12         2.8000e-01      2.11e-02       9.24e-02       7.75e-01    
      11             13         2.4995e-01      3.01e-02       2.06e-01       3.12e+00    
      12             14         2.4971e-01      2.31e-04       1.64e-03       8.62e-01    
      13             15         2.3872e-01      1.10e-02       2.76e-01       8.05e+00    
      14             16         2.3601e-01      2.71e-03       4.40e-04       6.14e-01    
      15             17         2.2888e-01      7.13e-03       2.19e-01       4.44e+00    
      16             18         2.2799e-01      8.92e-04       2.72e-04       1.23e+00    
      17             20         2.2618e-01      1.81e-03       3.46e-02       5.38e-01    
      18             21         2.2508e-01      1.10e-03       4.15e-02       2.11e+00    
      19             22         2.2472e-01      3.54e-04       1.15e-03       7.13e-01    
      20             25         2.2461e-01      1.11e-04       1.81e-03       1.36e+00    
      21             26         2.2456e-01      5.78e-05       4.50e-04       1.62e+00    
      22             27         2.2454e-01      1.09e-05       1.19e-04       2.53e-01    
      23             28         2.2454e-01      2.29e-06       2.46e-05       7.03e-01    
      24             32         2.2454e-01      0.00e+00       0.00e+00       7.03e-01    
`xtol` termination condition is satisfied.
Function evaluations 32, initial cost 3.9212e+00, final cost 2.2454e-01, first-order optimality 7.03e-01.
[31-5-2022 14:8:44] Least-squares routine finished.
[31-5-2022 14:8:44] Uncertainty analysis in progress...
[31-5-2022 14:15:7] Uncertainty analysis completed.
[31-5-2022 14:15:7] Model evaluation in progress...
[31-5-2022 14:18:36] Model evaluation completed.
Goodness-of-fit: 
========= ============= ============ ======= =========== 
 Dataset   Noise level   Reduced 𝛘2   RMSD       AIC     
========= ============= ============ ======= =========== 
   #1         0.009        1.703      0.012   -2678.968  
========= ============= ============ ======= =========== 
Model parameters: 
============ ======== ========================= ====== ======================================================= 
 Parameter    Value    95%-Confidence interval   Unit   Description                                            
============ ======== ========================= ====== ======================================================= 
 mean1        2.839    (2.767,2.911)              nm    Average inter-spin distance  #1                        
 mean2        4.551    (4.441,4.660)              nm    Average inter-spin distance  #2                        
 mean3        6.323    (5.678,6.969)              nm    Average inter-spin distance  #3                        
 chol11       0.398    (0.323,0.474)              nm    Cholesky factor ℓ11                                    
 chol22       0.414    (0.412,0.415)              nm    Cholesky factor ℓ22                                    
 chol33       0.325    (0.242,0.408)              nm    Cholesky factor ℓ33                                    
 chol21       -0.103   (-0.105,-0.101)            nm    Cholesky factor ℓ21                                    
 chol31       0.160    (0.077,0.243)              nm    Cholesky factor ℓ31                                    
 chol32       0.080    (0.004,0.155)              nm    Cholesky factor ℓ32                                    
 tau1         0.388    (0.380,0.395)              μs    First inter-pulse delay                                
 tau2         5.406    (5.200,5.600)              μs    Second inter-pulse delay                               
 tau3         0.431    (0.426,0.436)              μs    Third inter-pulse delay                                
 lamu         0.824    (0.392,1.257)             None   Amplitude of unmodulated pairwise pathway              
 lam1         0.035    (0.000,0.070)             None   Dipolar pathway #1 probability                         
 lam2         0.055    (0.005,0.105)             None   Dipolar pathway #2 probability                         
 lam3         0.004    (0.000,1.000)             None   Dipolar pathway #3 probability                         
 lam4         0.000    (0.000,1.000)             None   Dipolar pathway #4 probability                         
 lam5         0.000    (frozen)                  None   Dipolar pathway #5 probability                         
 lam6         0.000    (frozen)                  None   Dipolar pathway #6 probability                         
 lam7         0.000    (frozen)                  None   Dipolar pathway #7 probability                         
 lam8         0.000    (frozen)                  None   Dipolar pathway #8 probability                         
 lam9         0.000    (0.000,0.079)             None   Dipolar pathway #9 probability                         
 lam10        0.014    (0.000,0.585)             None   Dipolar pathway #10 probability                        
 lam11        0.000    (0.000,0.177)             None   Dipolar pathway #11 probability                        
 lam12        0.000    (0.000,0.129)             None   Dipolar pathway #12 probability                        
 lam13        0.000    (frozen)                  None   Dipolar pathway #13 probability                        
 lam14        0.000    (frozen)                  None   Dipolar pathway #14 probability                        
 lam15        0.004    (0.000,1.000)             None   Dipolar pathway #15 probability                        
 lam16        0.000    (0.000,1.000)             None   Dipolar pathway #16 probability                        
 lam17        0.000    (frozen)                  None   Dipolar pathway #17 probability                        
 lam18        0.000    (frozen)                  None   Dipolar pathway #18 probability                        
 lam19        0.000    (frozen)                  None   Dipolar pathway #19 probability                        
 lam20        0.000    (frozen)                  None   Dipolar pathway #20 probability                        
 lam21        0.000    (frozen)                  None   Dipolar pathway #21 probability                        
 lam22        0.000    (frozen)                  None   Dipolar pathway #22 probability                        
 lam23        0.000    (frozen)                  None   Dipolar pathway #23 probability                        
 lam24        0.000    (frozen)                  None   Dipolar pathway #24 probability                        
 conc_decay   40.665   (0.000,174.032)            μM    Spin concentration / Decay rate                        
 d            1.000    (frozen)                   μM    Stretch factor                                         
 scale        1.022    (1.022,1.022)             None   Echo amplitude in the abscense of dipolar modulations  
============ ======== ========================= ====== =======================================================
```

In [ ]:

```

```
