# Supplementary material for: Dipolar pathways in multi-spin and multi-dimensional dipolar EPR spectroscopy
Source: Phys Chem Chem Phys. 2022 Sep 8;24(37):22645–60. doi: 10.1039/d2cp03048a (PMC9516884; doi:10.1039/d2cp03048a)
Supplement: CP-024-D2CP03048A-s007 [file CP-024-D2CP03048A-s007.html]

analysis\_twospin\_DEER


In [ ]:

```
import numpy as  np 
import deerlab as dl 
import warnings
from multispin_analysis_module import *
import matplotlib.pyplot as plt
```

Analysis of the 4-pulse DEER datasets using two-spin models with non-parametric distance distributions.

The results show the effects of the incorrect modelling of multi-spin systems as two-spin systems.

---

## Triradical T111¶

---

In [ ]:

```
# Load experimental data
dBs = [7,5,4,3,2,0]
files = [f'.\\data\\triradical_T111_DEER_{dB}dB.DTA' for dB in dBs]
for n,file in enumerate(files):
    with warnings.catch_warnings():
        warnings.simplefilter("ignore")
        t,Vexp, descriptor = dl.deerload(file,full_output=True)
        t0, tau1_exp, tau2_exp = get_experimental_taus(descriptor)
    t += t0

    # Pre-processing
    Vexp = Vexp[:-10]
    t = t[:-10]
    Vexp = dl.correctphase(Vexp)
    Vexp /= np.max(Vexp) 

    # Construct the model
    r = np.linspace(2,5,80)
    experimentInfo = dl.ex_4pdeer(tau1_exp,tau2_exp,pathways=[1])
    Vmodel = dl.dipolarmodel(t,r,Bmodel=dl.bg_strexp,experiment=experimentInfo)

    # Fit the model to the data
    results = dl.fit(Vmodel,Vexp,ftol=1e-4)

    # Plot the results
    plt.figure(figsize=[8,3])
    plt.subplot(121)
    plt.plot(t,Vexp,'.',color='grey')
    plt.plot(t,results.model,'b')
    plt.xlabel('t (μs)')
    plt.ylabel('V(t) (arb.u.)')
    plt.subplot(122)
    plt.plot(r,results.P,'b')
    plt.fill_between(r,*results.PUncert.ci(95).T,color='b',alpha=0.4)
    plt.xlabel('r (nm)')
    plt.ylabel('P(r) (nm$^{-1}$)')
    plt.suptitle(f'Microwave power attenuation: {dBs[n]} dB')
    plt.tight_layout()
    plt.show()
```

---

## Triradial T011¶

---

In [ ]:

```
# Load experimental data
dBs = [11,9,8,7,5,3,0]
files = [f'.\\data\\triradical_T011_DEER_{dB}dB.DTA' for dB in dBs]
for n,file in enumerate(files):
    with warnings.catch_warnings():
        warnings.simplefilter("ignore")
        t,Vexp, descriptor = dl.deerload(file,full_output=True)
        t0, tau1_exp, tau2_exp = get_experimental_taus(descriptor)
    t += t0

    # Pre-processing
    Vexp = Vexp[:-10]
    t = t[:-10]
    Vexp = dl.correctphase(Vexp)
    Vexp /= np.max(Vexp) 

    # Construct the model
    r = np.linspace(2,5,80)
    experimentInfo = dl.ex_4pdeer(tau1_exp,tau2_exp,pathways=[1])
    Vmodel = dl.dipolarmodel(t,r,Bmodel=dl.bg_strexp,experiment=experimentInfo)

    # Fit the model to the data
    results = dl.fit(Vmodel,Vexp,ftol=1e-4)

    # Plot the results
    plt.figure(figsize=[8,3])
    plt.subplot(121)
    plt.plot(t,Vexp,'.',color='grey')
    plt.plot(t,results.model,'b')
    plt.xlabel('t (μs)')
    plt.ylabel('V(t) (arb.u.)')
    plt.subplot(122)
    plt.plot(r,results.P,'b')
    plt.fill_between(r,*results.PUncert.ci(95).T,color='b',alpha=0.4)
    plt.xlabel('r (nm)')
    plt.ylabel('P(r) (nm$^{-1}$)')
    plt.suptitle(f'Microwave power attenuation: {dBs[n]} dB')
    plt.tight_layout()
    plt.show()
```

---

## Triple MTSL-labeled Rpo47 protein complex¶

---

In [ ]:

```
# Load the experimental data
dBs = [0,6,9]
files = [f'.\\data\\triradical_rpo47_DEER_{dB}dB.DTA' for dB in dBs]
for n,file in enumerate(files):
    with warnings.catch_warnings():
        warnings.simplefilter("ignore")
        t,Vexp, descriptor = dl.deerload(file,full_output=True)
        t0, tau1_exp, tau2_exp = get_experimental_taus(descriptor)
    t += t0

    # Pre-processing
    Vexp = Vexp[:-10]
    t = t[:-10]
    Vexp = dl.correctphase(Vexp)
    Vexp /= np.max(Vexp) 

    # Construct the model
    r = np.linspace(1,8,120)
    experimentInfo = dl.ex_4pdeer(tau1_exp,tau2_exp,pathways=[1])
    Vmodel = dl.dipolarmodel(t,r,experiment=experimentInfo)

    # Fit the model to the data
    results = dl.fit(Vmodel,Vexp,ftol=1e-4)

    # Plot the results
    plt.figure(figsize=[8,3])
    plt.subplot(121)
    plt.plot(t,Vexp,'.',color='grey')
    plt.plot(t,results.model,'b')
    plt.xlabel('t (μs)')
    plt.ylabel('V(t) (arb.u.)')
    plt.subplot(122)
    plt.plot(r,results.P,'b')
    plt.fill_between(r,*results.PUncert.ci(95).T,color='b',alpha=0.4)
    plt.xlabel('r (nm)')
    plt.ylabel('P(r) (nm$^{-1}$)')
    plt.suptitle(f'Microwave power attenuation: {dBs[n]} dB')
    plt.tight_layout()
    plt.show()
```

---

## Q5¶

---

In [ ]:

```
# Load experimental data
dBs = [0,2,4,6]
files = [f'.\\data\\tetraradical_DEER_{dB}dB.DSC' for dB in dBs]
for n,file in enumerate(files):
    with warnings.catch_warnings():
        warnings.simplefilter("ignore")
        t,Vexp, descriptor = dl.deerload(file,full_output=True)
        t0, tau1_exp, tau2_exp = get_experimental_taus(descriptor)
    t += t0

    # Pre-processing
    Vexp = dl.correctphase(Vexp)
    Vexp /= np.max(Vexp) 

    # Construct the model
    r = np.linspace(1,5,80)
    experimentInfo = dl.ex_4pdeer(tau1_exp,tau2_exp,pathways=[1])
    Vmodel = dl.dipolarmodel(t,r,experiment=experimentInfo)

    # Fit the model to the data
    results = dl.fit(Vmodel,Vexp,ftol=1e-4)

    # Plot the results
    plt.figure(figsize=[8,3])
    plt.subplot(121)
    plt.plot(t,Vexp,'.',color='grey')
    plt.plot(t,results.model,'b')
    plt.xlabel('t (μs)')
    plt.ylabel('V(t) (arb.u.)')
    plt.subplot(122)
    plt.plot(r,results.P,'b')
    plt.fill_between(r,*results.PUncert.ci(95).T,color='b',alpha=0.4)
    plt.xlabel('r (nm)')
    plt.ylabel('P(r) (nm$^{-1}$)')
    plt.suptitle(f'Microwave power attenuation: {dBs[n]} dB')
    plt.tight_layout()
    plt.show()
```

In [ ]:

```

```
