# Supplementary material for: Dipolar pathways in multi-spin and multi-dimensional dipolar EPR spectroscopy
Source: Phys Chem Chem Phys. 2022 Sep 8;24(37):22645–60. doi: 10.1039/d2cp03048a (PMC9516884; doi:10.1039/d2cp03048a)
Supplement: CP-024-D2CP03048A-s063 [file CP-024-D2CP03048A-s063.pdf]

Electronic Supplementary Material (ESI) for Physical Chemistry Chemical Physics.
This journal is © the Owner Societies 2022

Dipolar pathways in multi-spin and multi-dimensional dipolar EPR spectroscopy (Supporting Information)

Luis Fábregas-Ibáñez(1), Valerie Mertens(1), Irina Ritsch (1), Tona von Hagens(1), Stefan Stoll(2), and Gunnar Jeschke(1)

1) ETH Zurich, Laboratory of Physical Chemistry, Vladimir-Prelog-Weg 2, Zurich, Switzerland

2) University of Washington, Department of Chemistry, Seattle, WA 98195, Washington, USA

Table of contents

I. Files and data
II. Materials and methods
III. Results of the analysis: Parameter tables
IV. Results of the analysis: Full traces
V. Two-spin model analyses

I. Files and data

This **`SI.zip`** package contains the following files and folders:

- **`SI.pdf`**
  This file.

- **`data/*`**
  Folder containing all experimental datasets presented in the publication.

- **`HSC_simulations/*`**
  Folder containing the Harmonic Segmented Chain (HSC) simulation script, as well as the data corresponding to the simulations presented in the publication.

- **`MMMx_simulations/*`**
  Folder containing the MMMx simulation script, as well as the data corresponding to the simulations presented in the publication.

- **`multispin_dipolar_pathway_calculator.ipynb`**
  Jupyter notebook with the function for calculating all possible theoretical dipolar pathways for arbitrary pulse sequences and multi-spin systems.

- **`multispin_analysis_module.py`**
  Python module containing the collection of custom functions required to run the analysis scripts

- **`analysis_multispin_DEER.ipynb/.py/.html`**
  Jupyter notebook containing the analysis of the DEER data including scripts, figures, and fits with confidence intervals. A Python script copy is provided for executing the contents of the Jupyter notebook if Jupyter is not installed. An HTML copy is provided for reading the contents of the Jupyter notebook if Python and Jupyter are not installed.

- **`analysis_multispin_TRIER.ipynb/.py/.html`**
  Jupyter notebook containing the analysis of the TRIER data including scripts, figures, and fits with confidence intervals. A Python script copy is provided for executing the contents of the Jupyter notebook if Jupyter is not installed. An HTML copy is provided for reading the contents of the Jupyter notebook if Python and Jupyter are not installed.

- **`analysis_twospin_DEER.ipynb/.py/.html`**
  Jupyter notebook containing the local and global analysis of the 4-pulse DEER data using two-spin models with non-parametric distance distributions. including scripts, figures, and fits with confidence intervals.
  A Python script copy is provided for executing the contents of the Jupyter notebook if Jupyter is not installed. An HTML copy is provided for reading the contents of the Jupyter notebook if Python and Jupyter are not installed.

- **LICENSE**
  License disclaimer pertaining all code provided here.

The following software were used to run the scripts provided in this SI:

- Windows10
- Python 3.8 (specific packages are listed in the scripts)
- DeerLab v0.14.3
- Matlab 2019b
- MMMx 1.0

II. Materials and methods

OligoPPE samples

Sample preparation

The oligoPPE substances T011 and T111 were synthesized in the group of Prof. Adelheidt Godt, Bielefeld University, Germany [Jeschke2009]. The samples with total nitroxide concentrations of 100 − 300 µmol/L were produced by dissolving 0.1 mg of the pure sample substance in perdeuterated o-terphenyl, synthesized by Herbert Zimmermann, MPI for Medical Research Heidelberg, heated slightly above the melting point of 60°C. The crystallized samples were powdered and filled into EPR quartz tubes with maximum diameters of 3 mm (for DEER measurements) or 1.6 mm (for TRIER measurements). To avoid crystallization of the solvent and thus intermolecular aggregation, immediately before introducing the sample tube into the pre-cooled EPR probehead (50 K), the samples were melted again in the hot air stream of a heat gun and shock-frozen in liquid nitrogen. The samples were stored at 4°C. The solvents of all model compounds were deuterated, as this extends the accessible upper distance limit and improves the resolution of the DEER and TRIER experiments. Detailed descriptions of the syntheses, the spin labeling procedure and further characteristics of those samples can be found in the main text and in the Supporting Information of [Jeschke2009, Jeschke2010, Sajid2009].

The tetraradical Q5 was synthesized by Olav Schiemann and published by Bode et al. in [Bode2007].

Measurements

Four-pulse DEER experiments were measured with the standard pulse sequence shown in Figure 6.1 on either a Bruker Elexsys II E580 or a Bruker Elexsys E680 pulse EPR spectrometer (Bruker Biospin, Karlsruhe, Germany), both operating at at X-band frequencies (9.3 GHz), equipped with a Bruker Flexline 3 mm split-ring resonator overcoupled to Q ≈ 100. A temperature of 50 K was used with a shot repetition time of 4 ms. For the Elexsys E580, the second microwave frequency (ELDOR frequency channel ν_{pump}) was provided by an E8257D PSG Analog Signal Generator (Agilent, Santa Clara, CA) limited to a power setting of 17 dBm and fed into one of the mw pulse forming units of the spectrometer. For the Elexsys II E580 and the E680 spectrometers, the built-in second frequency source was used. The DEER experiments were performed with the pump frequency ν_{pump} at the maximum of the nitroxide spectrum. The maximum was determined for each substance individually with a field-swept electron spin echo (FSESE) experiment. The frequency of the observer pulse was increased by 65 MHz relative to the frequency of the pump channel. Observer pulse lengths were 32 ns for the π/2 and π pulses. In all experiments, receiver offset was cancelled by a [(+x)-(-x)] phase cycle applied to the first excitation pulse. Variation of the inversion efficiency of the DEER experiment was achieved by variation of the pump pulse power and subsequent phase readjustment of the observer pulses.

TRIER experiments were performed on a home-built high-power ultra-wideband Q-Band spectrometer [Doll2014], that is equipped with a 8 GSa/s arbitrary waveform generator and a 200 W traveling wavetube amplifier. Essential for TRIER experiments is the used home-built ultra-broad-band pent loop-gap resonator with a loaded quality factor around 120 that accepts capillaries with an outer diameter of 1.6 nm. Both TRIER experiments were performed at 50 K and with a shot repetition time of 2 ms. The center of the nitroxide spectrum was placed in the center of the broad-band resonator and the TRIER observer frequency was set 45-50 MHz lower than the spectral maximum. Pump pulses were placed symmetrically around the observer pulses at an offset of +- 90 MHz with respect to the center of the observer pulses. For compound T011, a gaussian-shaped observer sequence and asymmetric hyperbolic secant pump pulses were employed. TRIER experiments on T111 were performed using chirped observer pulses and asymmetric hyperbolic secant pump pulses. In both cases, the amplitudes of the observer pulses were optimized for maximal TRIER echo amplitude, while pump pulse amplitudes were adjusted as a compromise between maximal inversion efficiency and a clean inversion profile without significant side-bands. To ensure a constant adiabaticity over the entire pulse excitation range, resonator

compensation was applied for all frequency-swept pulses [Doll2017]. Further details on the used pulses can be found in tables 1 and 2. For T011, TRIER inter-pulse delays were set as follows: τ_1 = 400 ns, τ_2 = 4600 ns, τ_3 = 500 ns. For T111 τ_1 = 400 ns, τ_2 = 2800 ns and τ_3 = 500 ns were used. To record the TRIER data, both pump pulses were incremented independently in steps of 40 ns. With 70 points per dimension this resulted in a TRIER signal with a total length of 2800 ns per dimension for T011. For T111, 116 points were recorded along each dimension, resulting in a total sequence length of 4640 ns along both dimensions. Phase cycling was applied as described in [Pribitzer2017].

Table S1: TRIER experimental shaped pulse parameters for the T011 triradical measurements. For each pulse, its role, shape, and length Tp are indicated. For the Gauss pulses, the full-width at half-maximum is indicated as well. For the hypersecant (HS) pulses the excitation bandwidth, the order of steepness of the left and right frequency flanks, and truncation parameter are given as well.

Pulse no.	*1*	*2*	*3*	*4*	*5*	*6*
Role	obs π/2	obs π	pump 1	obs π	pump 2	obs π
Pulse Shape	gauss	gauss	HS	gauss	HS	gauss
Pulse length [ns]	100	100	100	100	100	100
Gauss FWHM [ns]	42.47	42.47		42.47		42.47
HS bandwidth [MHz]			60		60	
HS left steep. order			6		1	
HS right steep. order			1		6	
HS truncation			10		10	

Table S2: TRIER experimental pulse parameters for T111 triradical measurements. For each pulse, its role, shape, and length Tp are indicated. For the chirp pulses, the bandwidth and rise time are given. For the hypersecant (HS) pulses the excitation bandwidth, the order of steepness of the left and right frequency flanks, and truncation parameter are given as well.

Pulse no.	*1*	*2*	*3*	*4*	*5*	*6*
Role	obs π/2	obs π	pump 1	obs π	pump 2	obs π
Pulse Shape	chirp	chirp	HS	chirp	HS	chirp
Pulse length [ns]	100	100	100	100	100	50
Bandwidth [MHz]	25	25	60	25	60	25
Chirp rise time [ns]	15	15		15		22.5
HS left steep. order			6		1	
HS right steep. order			1		6	
HS truncation			10		10	

Rpo4/7 sample

Protein purification and spin labelling

The heat-stable complex of subunits Rpo4 and Rpo7 (also known as F and E, respectively) of the archeal RNA polymerase of M. jannaschii were purified according to established protocols. [Klose2012, Werner2002]. For more details, see also the supporting information of [Pribitzer2018]. The labelling sites for the Rpo4/7 complex (Rpo4: C36, G63C; Rpo7: V49C) were selected from a larger set of sites reported in [Klose2012], where all pairwise distances from DEER experiments have been measured. The two subunits, Rpo4 and Rpo7, of the Rpo4/7 complex were individually over-expressed in Escherichia coli following the established protocols. Protein concentrations were determined with a NanoDrop Spectrophotometer ND-1000 (Witec AG) using the calculated extinction coefficients [Gasteiger2003] of ϵ = 29.34 $\mathrm{L\,mmol^{-1}cm^{-1}}$ for Rpo4/7. The individual subunits or the complex of Rpo4/7 were spin labelled with tenfold molar excess of MTSL ((1-oxyl-2,2,5,5-tetramethylpyrroline-3methyl)methanethiosulfonate, Toronto Research Chemicals) over cysteine concentration at a protein concentration of 10 µM. Unreacted spin label was washed out by repeated concentration and re-dilution in a 10 kDa MWCO centrifugal concentrator (Vivaspin-500, 10 kDa MWCO, Sigma-Aldrich). Removal of the free nitroxide label and spin label attachment was checked by CW EPR spectroscopy. The final protein samples were lyophilised and resuspended in a D_2O/d8-glycerol mixture (1:1 by volume) and transferred into the 3 mm (outer diameter) sample tube.

Measurements

Four-pulse DEER experiments were measured with the standard pulse sequence shown in Figure 6.1 on a home-built Q-band spectrometer. The detection frequency ν_d was 34.5-34.8 GHz. The pump/detection frequency offsets were: $\Delta\nu = \nu_p - \nu_d = 0.1$ GHz. Pumping was always performed at the maximum of the nitroxide spectrum. The DEER measurements were performed at 50 K with a shot repetition time of 4 ms. Pulse powers were optimized using nutation experiments. All pulses had a length of 12 ns. The value of the first refocusing delay was 400 ns. A phase cycle [+(+x)-(-x)] was applied to the π/2 observer pulse to cancel receiver offset. Variation of the inversion efficiency of the DEER experiment was again achieved by variation of the pump pulse power and subsequent phase readjustment of the observer pulses.

TRIER experiments were measured with a home-built Q-band AWG spectrometer. All details on the pulse sequence and pulse shape parameters can be found in [Pribitzer2018].

References

[Bode2007] B. E. Bode, D. Margraf, J. Plackmeyer, G. Dürner, T. F. Prisner and O. Schiemann, J. Am. Chem. Soc., 2007, 129, 6736-45.

[Doll2014] A. Doll and G. Jeschke, Journal of Magnetic Resonance, 2014,246, 18–26.

[Doll2017] A. Doll and G. Jeschke, Journal of Magnetic Resonance, 2017,280, 46-62.

[Gasteiger2003] E. Gasteiger, A. Gattiker, C. Hoogland, I. Ivanyi, R. D. Appel, and A. Bairoch, Nucleic Acids Research, 2003, 31(13), 3784-3788.

[Jeschke2009] G. Jeschke, M. Sajid, M. Schulte and A. Godt, Phys. Chem. Chem. Phys., 2009, 11, 6580-91.

[Jeschke2010] G. Jeschke, M. Sajid, M. Schulte, N. Ramezanian, A. Volkov, H. Zimmermann and A. Godt, J. Am. Chem. Soc., 2010, 132, 10107-17.

[Klose2012] D. Klose, J. P. Klare, D. Grohmann, C. W. M. Kay, F. Werner, and H.-J. Steinhoff. PLoS ONE, 7(6), 2012.

[Pribitzer2017] S. Pribitzer, M. Sajid, M. Hülsmann, A. Godt, and G. Jeschke, Journal of Magnetic Resonance, 2017, 282, 119-128.

[Pribitzer2018] S. Pribitzer, L. Fábregas Ibáñez, C. Gmeiner, I. Ritsch, D. Klose, M. Sajid, M. Hülsmann, A. Godt, and G. Jeschke, Applied Magnetic Resonance, 2018, 49(11), 1253-1279.

[Sajid2009] M. Sajid, G. Jeschke, M. Wiebcke and A. Godt, Chem. A Eur. J., 2009, 15, 12960-2.

[Werner2002] F. Werner and R. O. Weinzierl, Molecular Cell, 10(3), 2002, 1097-2765.

III. Results of the analysis: Full traces

Figures S1-S3 show the full traces for the oligoPPE 4-pulse DEER datasets (in the main text, only the first three microseconds of the trace are shown for clarity).

Analysis script: `analysis_multispin_DEER.ipynb` , `analysis_multispin_TRIER.ipynb`

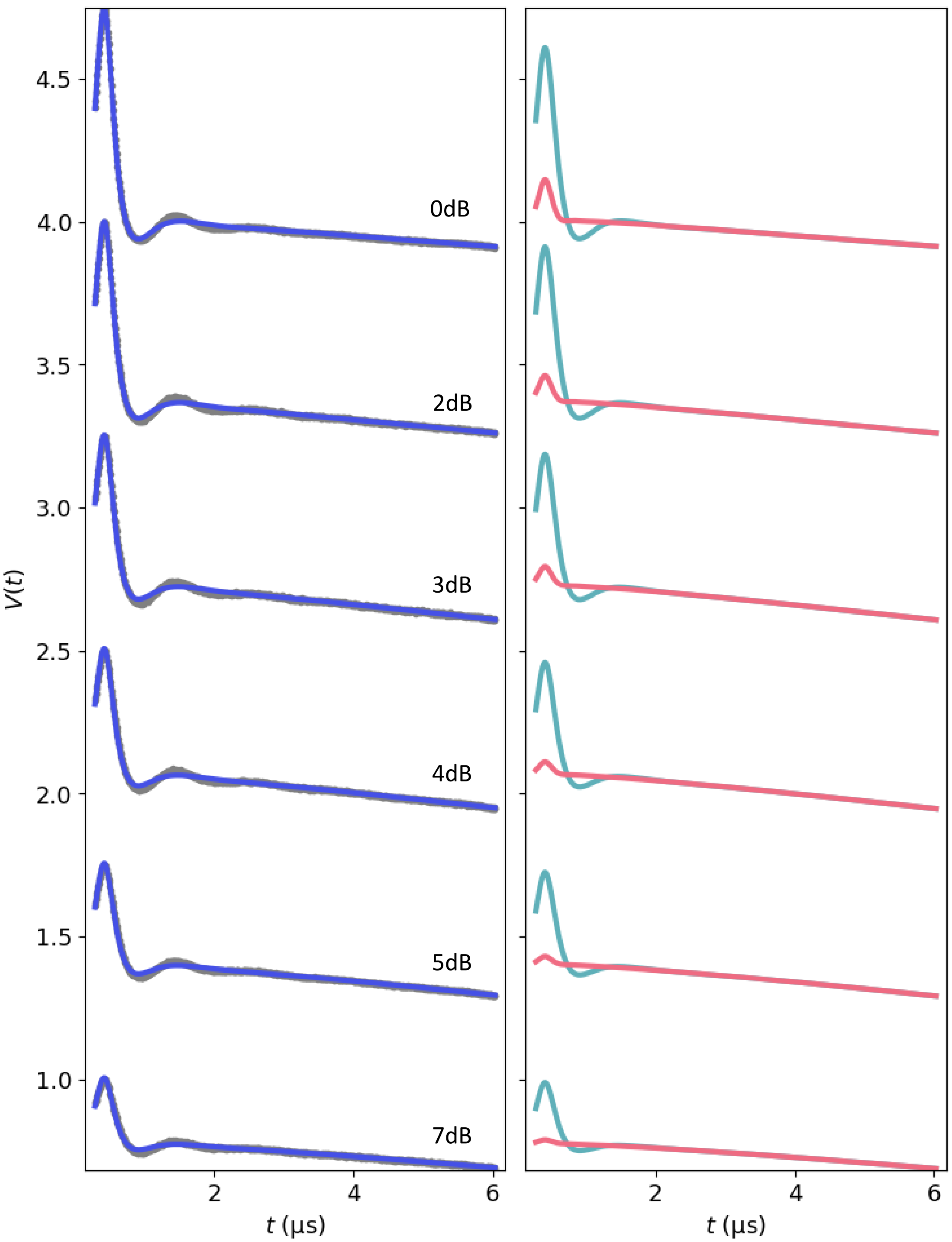

Figure S1: Global analysis with DeerLab of a series of X-band 4-pulse DEER of oligoPPE triradical T111 at different levels of microwave power attenuation (indicated next to each dataset). The experimental datasets are shown in the left panel as grey dots along with the model fits and unmodulated contributions shown as solid and dashed blue lines, respectively. The contributions from two-spin interactions are shown as turquoise lines and the contributions arising from three-spin interactions are shown as red lines. The full traces are shown in this figure.

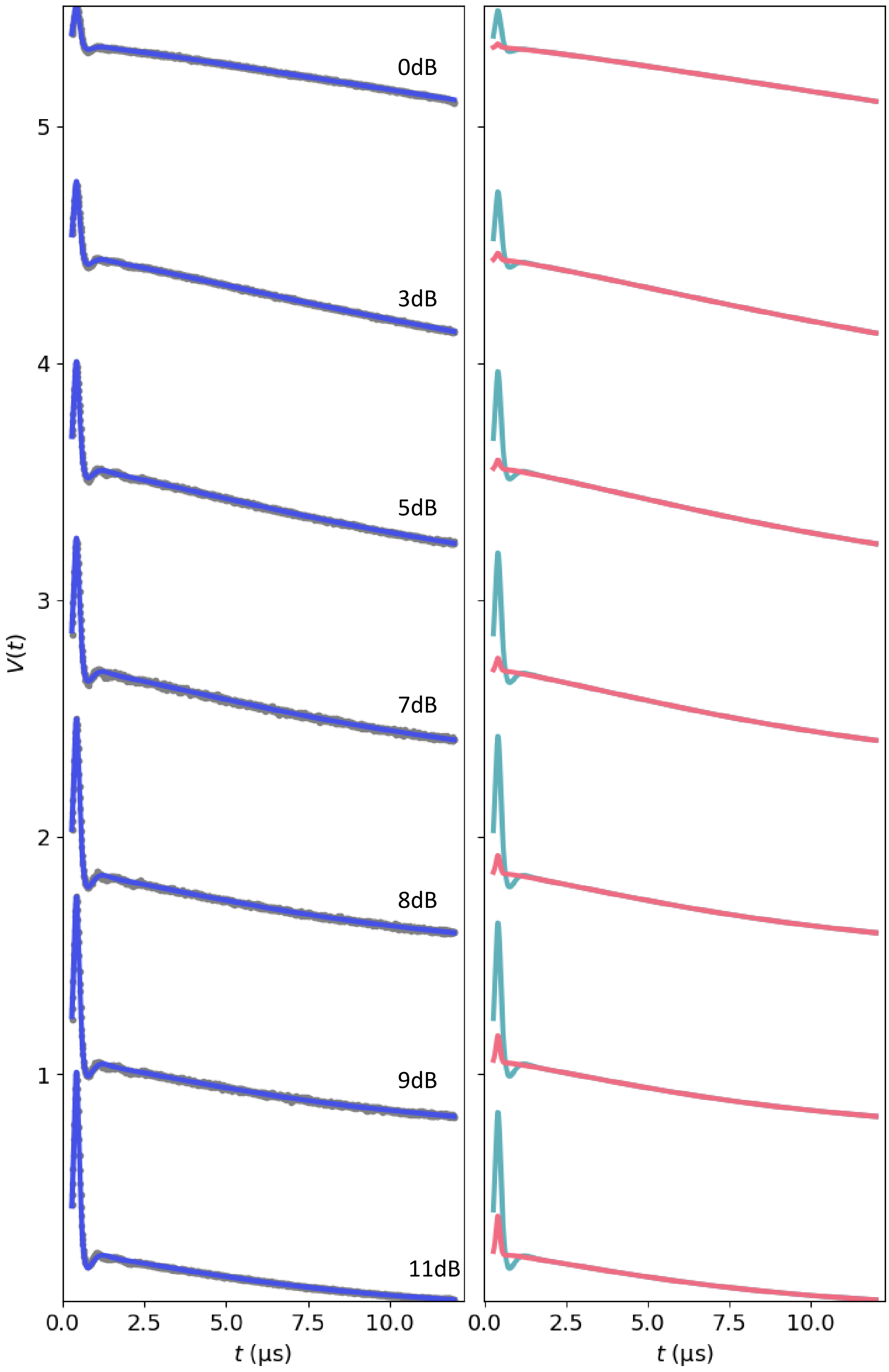

Figure S2: Global analysis with DeerLab of a series of X-band 4-pulse DEER of oligoPPE triradical T011 at different levels of microwave power attenuation (indicated next to each dataset). The experimental datasets are shown in the left panel as grey dots along with the model fits and unmodulated contributions shown as solid and dashed blue lines, respectively. The contributions from two-spin interactions are shown as turquoise lines and the contributions arising from three-spin interactions are shown as red lines. The full traces are shown in this figure.

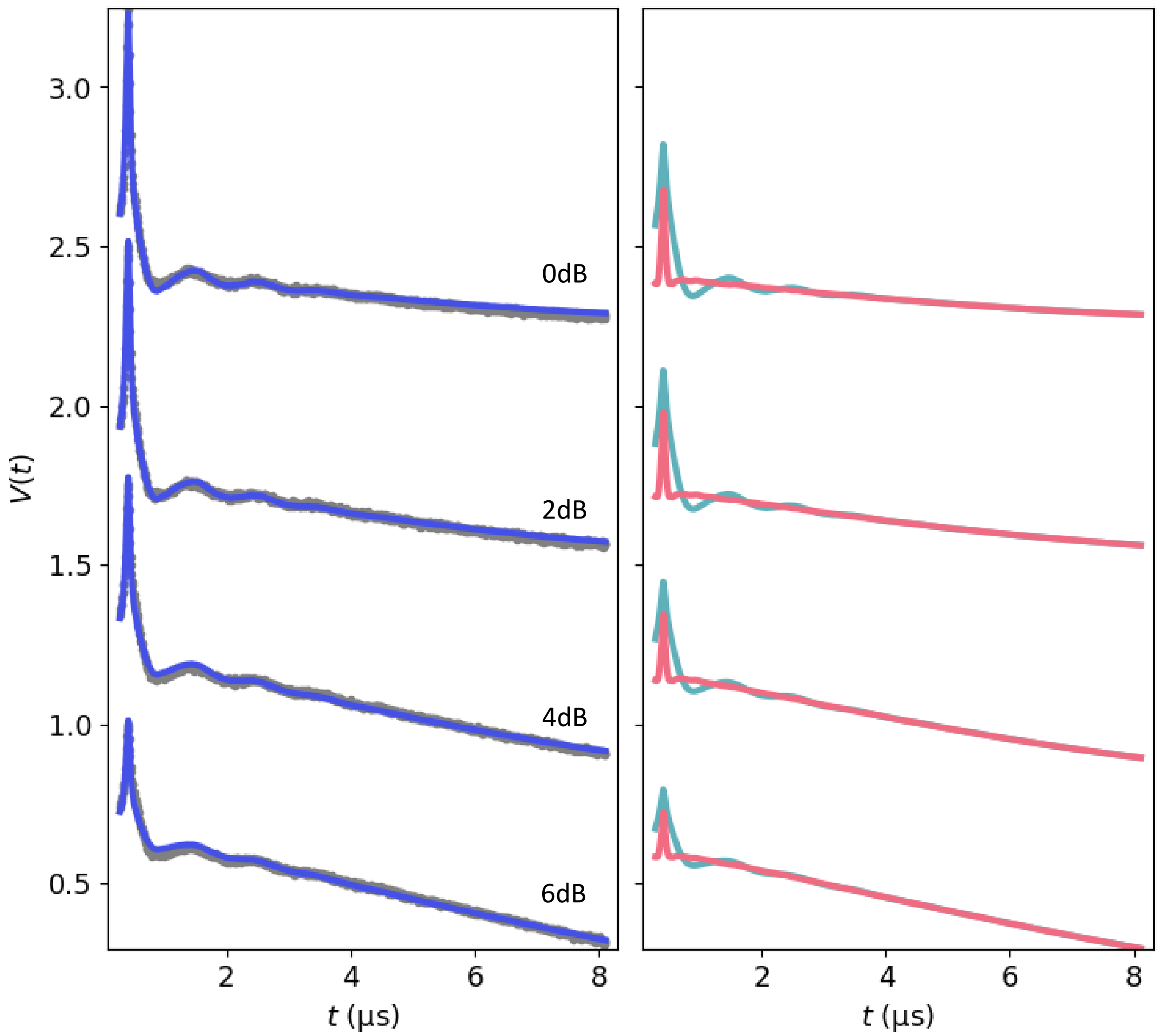

Figure S3: Global analysis with DeerLab of a series of X-band 4-pulse DEER of the oligoPPE tetraradical at different levels of microwave power attenuation (indicated next to each dataset). The experimental datasets are shown in the left panel as grey dots along with the model fits and unmodulated contributions shown as solid and dashed blue lines, respectively. The contributions from two-spin interactions are shown as turquoise lines and the contributions arising from three-spin interactions are shown as red lines. The full traces are shown in this figure.

IV. Results of the analysis: Parameter tables

Tables S3-S9 collect the DeerLab summaries for each of the global models used to analyze the 4-pulse DEER and TRIER datasets. The tables contain information on the goodness-of-fit statistics and fitted values (with 95%-confidence intervals) for each model parameter fitted to the data.

Analysis script: `analysis_multispin_DEER.ipynb` , `analysis_multispin_TRIER.ipynb`

Table S3: DeerLab's results summary of the analysis of the T111 triradical oligoPPE 4-pulse DEER data. The first table collects the estimated noise level (standard deviation), reduced chi-square, RMSD and AIC values of the individual dataset fits. The second table shows the DeerLab parameter name, fitted value, 95%-confidence intervals, unit, and description for the individual model parameters fitted during the analysis. The suffix "_n" is used to denote a parameter belonging to the *n*-th dataset.

Goodness-of-fit:

Dataset	Noise level	Reduced χ2	RMSD	AIC
#1	0.002	5.249	0.004	-1920.826
#2	0.001	11.465	0.005	-1785.179
#3	0.002	9.042	0.005	-1689.361
#4	0.002	7.355	0.006	-1600.525
#5	0.002	9.332	0.006	-1635.156
#6	0.001	16.154	0.005	-1703.425

Model parameters:

Parameter	Value	95%-Confidence interval	Unit	Description
mean1	3.481	(3.396,3.565)	nm	Average inter-spin distance #1
mean2	3.460	(3.327,3.594)	nm	Average inter-spin distance #2
mean3	3.537	(3.420,3.654)	nm	Average inter-spin distance #3
chol11	0.464	(0.464,0.464)	nm	Cholesky factor ℓ11
chol22	0.462	(0.462,0.463)	nm	Cholesky factor ℓ22
chol33	0.461	(0.461,0.461)	nm	Cholesky factor ℓ33
chol21	0.002	(0.002,0.002)	nm	Cholesky factor ℓ21
chol31	0.000	(0.000,0.000)	nm	Cholesky factor ℓ31
chol32	-0.001	(-0.001,-0.000)	nm	Cholesky factor ℓ32
tau1	0.411	(0.411,0.412)	μs	First inter-pulse delay
tau2	6.099	(6.099,6.099)	μs	Second inter-pulse delay
lamu_1	1.077	(0.720,1.433)	None	Amplitude of unmodulated pathway
lam1_1	0.031	(0.012,0.049)	None	Amplitude of pairwise pathway #1
lam23	0.000	(frozen)	None	Amplitude of pairwise pathways #2 and #3
decay	0.144	(0.128,0.160)	μs^{-1}	Decay rate
d	1.368	(1.322,1.414)	μM	Stretch factor
lamu_2	1.092	(0.831,1.353)	None	Amplitude of unmodulated pairwise pathway
lam1_2	0.045	(0.026,0.064)	None	Amplitude of pairwise pathway #1
lamu_3	1.069	(0.865,1.272)	None	Amplitude of unmodulated pairwise pathway
lam1_3	0.057	(0.038,0.076)	None	Amplitude of pairwise pathway #1
lamu_4	1.027	(0.870,1.184)	None	Amplitude of unmodulated pairwise pathway
lam1_4	0.072	(0.054,0.091)	None	Amplitude of pairwise pathway #1
lamu_5	1.034	(0.909,1.158)	None	Amplitude of unmodulated pairwise pathway
lam1_5	0.084	(0.067,0.101)	None	Amplitude of pairwise pathway #1
lamu_6	0.949	(0.873,1.026)	None	Amplitude of unmodulated pairwise pathway
lam1_6	0.111	(0.098,0.125)	None	Amplitude of pairwise pathway #1
scale_1	1.005	(1.004,1.005)	None	Echo amplitude
scale_2	1.006	(1.005,1.006)	None	Echo amplitude
scale_3	1.007	(1.006,1.009)	None	Echo amplitude
scale_4	1.003	(1.001,1.006)	None	Echo amplitude
scale_5	1.001	(0.996,1.005)	None	Echo amplitude
scale_6	0.995	(0.992,0.998)	None	Echo amplitude

Table S4: DeerLab's results summary of the analysis of the T011 triradical oligoPPE 4-pulse DEER data. The first table collects the estimated noise level (standard deviation), reduced chi-square, RMSD and AIC values of the individual dataset fits. The second table shows the DeerLab parameter name, fitted value, 95%-confidence intervals, unit, and description for the individual model parameters fitted during the analysis. The suffix "_n" is used to denote a parameter belonging to the *n*-th dataset.

Goodness-of-fit:

Dataset	Noise level	Reduced χ2	RMSD	AIC
#1	0.002	3.252	0.004	-2463.820
#2	0.004	1.673	0.005	-2162.126
#3	0.004	1.238	0.005	-2239.828
#4	0.006	1.021	0.006	-2032.513
#5	0.003	1.852	0.004	-2376.629
#6	0.003	1.869	0.003	-2583.196
#7	0.002	1.412	0.003	-2758.198

Model parameters:

Parameter	Value	95%-Confidence interval	Unit	Description
mean1	3.121	(3.093,3.150)	nm	Average inter-spin distance #1
mean2	3.123	(3.097,3.149)	nm	Average inter-spin distance #2
mean3	3.091	(3.055,3.127)	nm	Average inter-spin distance #3
chol11	0.465	(0.465,0.465)	nm	Cholesky factor ℓ11
chol22	0.436	(0.436,0.436)	nm	Cholesky factor ℓ22
chol33	0.437	(0.437,0.437)	nm	Cholesky factor ℓ33
chol21	0.035	(0.034,0.035)	nm	Cholesky factor ℓ21
chol31	-0.023	(-0.023,-0.022)	nm	Cholesky factor ℓ31
chol32	0.042	(0.042,0.042)	nm	Cholesky factor ℓ32
tau1	0.418	(0.417,0.418)	µs	First inter-pulse delay
tau2	12.099	(12.099,12.099)	µs	Second inter-pulse delay
lamu_1	0.908	(0.813,1.002)	None	Amplitude of unmodulated pairwise pathway
lam1_1	0.121	(0.102,0.140)	None	Amplitude of pairwise pathway #1
lam23	0.000	(frozen)	None	Amplitude of pairwise pathways #2 and #3
decay	0.251	(0.237,0.266)	μs^{-1}	Decay rate
d	1.246	(1.236,1.256)	µM	Stretch factor
lamu_2	1.013	(0.881,1.146)	None	Amplitude of unmodulated pathways
lam1_2	0.095	(0.075,0.115)	None	Amplitude of pairwise pathway #1
lamu_3	1.145	(0.959,1.330)	None	Amplitude of unmodulated pathways
lam1_3	0.073	(0.053,0.094)	None	Amplitude of pairwise pathway #1
lamu_4	1.176	(0.945,1.406)	None	Amplitude of unmodulated pathways
lam1_4	0.060	(0.039,0.080)	None	Amplitude of pairwise pathway #1
lamu_5	1.169	(0.900,1.438)	None	Amplitude of unmodulated pathways
lam1_5	0.050	(0.030,0.070)	None	Amplitude of pairwise pathway #1
lamu_6	1.007	(0.793,1.220)	None	Amplitude of unmodulated pathways
lam1_6	0.047	(0.030,0.065)	None	Amplitude of pairwise pathway #1
lamu_7	0.839	(0.643,1.035)	None	Amplitude of unmodulated pathways
lam1_7	0.037	(0.022,0.051)	None	Amplitude of pairwise pathway #1
scale_1	1.010	(1.008,1.012)	None	Echo amplitude
scale_2	1.001	(1.000,1.001)	None	Echo amplitude
scale_3	1.000	(0.994,1.006)	None	Echo amplitude
scale_4	1.014	(1.002,1.025)	None	Echo amplitude
scale_5	1.008	(0.991,1.024)	None	Echo amplitude
scale_6	1.018	(1.017,1.019)	None	Echo amplitude
scale_7	1.010	(1.010,1.011)	None	Echo amplitude

Table S5: DeerLab's results summary of the analysis of the Rpo47 triradical protein complex 4-pulse DEER data. The first table collects the estimated noise level (standard deviation), reduced chi-square, RMSD and AIC values of the individual dataset fits. The second table shows the DeerLab parameter name, fitted value, 95%-confidence intervals, unit, and description for the individual model parameters fitted during the analysis. The suffix "_n" is used to denote a parameter belonging to the n-th dataset.

Goodness-of-fit:

Dataset	Noise level	Reduced χ2	RMSD	AIC
#1	0.013	1.893	0.018	-1129.670
#2	0.010	1.883	0.014	-1492.525
#3	0.015	1.267	0.017	-1173.697

Model parameters:

Parameter	Value	95%-Confidence interval	Unit	Description
mean1	2.401	(2.329,2.473)	nm	Average inter-spin distance #1
mean2	4.383	(4.334,4.432)	nm	Average inter-spin distance #2
mean3	6.341	(6.266,6.415)	nm	Average inter-spin distance #3
chol11	0.415	(0.385,0.446)	nm	Cholesky factor ℓ11
chol22	0.307	(0.273,0.341)	nm	Cholesky factor ℓ22
chol33	0.294	(0.237,0.352)	nm	Cholesky factor ℓ33
chol21	-0.047	(-0.049,-0.045)	nm	Cholesky factor ℓ21
chol31	0.130	(0.083,0.177)	nm	Cholesky factor ℓ31
chol32	0.137	(0.069,0.204)	nm	Cholesky factor ℓ32
tau1	0.406	(0.403,0.409)	µs	First inter-pulse delay
tau2	9.001	(8.900,9.100)	µs	Second inter-pulse delay
lamu_1	0.989	(0.755,1.223)	None	Amplitude of unmodulated pathways
lam1_1	0.080	(0.055,0.105)	None	Amplitude of pairwise pathway #1
lam23_1	0.019	(0.003,0.035)	None	Amplitude of pairwise pathways #2 and #3
conc	100.236	(86.295,114.177)	µM	Spin concentration
lamu_2	1.013	(0.733,1.294)	None	Amplitude of unmodulated pathways
lam1_2	0.063	(0.036,0.090)	None	Amplitude of pairwise pathway #1
lam23_2	0.013	(0.001,0.025)	None	Amplitude of pairwise pathways #2 and #3
lamu_3	1.002	(0.658,1.347)	None	Amplitude of unmodulated pathways
lam1_3	0.050	(0.020,0.079)	None	Amplitude of pairwise pathway #1
lam23_3	0.006	(0.000,0.014)	None	Amplitude of pairwise pathways #2 and #3
scale_1	1.230	(1.223,1.237)	None	Echo amplitude
scale_2	1.175	(1.174,1.176)	None	Echo amplitude
scale_3	1.095	(1.094,1.097)	None	Echo amplitude

Table S6: DeerLab's results summary of the analysis of the tetraradical oligoPPE 4-pulse DEER data. The first table collects the estimated noise level (standard deviation), reduced chi-square, RMSD and AIC values of the individual dataset fits. The second table shows the DeerLab parameter name, fitted value, 95%-confidence intervals, unit, and description for the individual model parameters fitted during the analysis. The suffix "_n" is used to denote a parameter belonging to the n-th dataset.

Goodness-of-fit:

Dataset	Noise level	Reduced χ2	RMSD	AIC
#1	0.004	5.013	0.008	-1312.661
#2	0.004	3.527	0.008	-1369.426
#3	0.006	1.402	0.007	-1469.284
#4	0.004	6.440	0.009	-1303.175

Model parameters:

Parameter	Value	95%-Confidence interval	Unit	Description
mean1	3.074	(2.661,3.487)	nm	Average inter-spin distance #1
mean2	3.793	(1.000,8.000)	nm	Average inter-spin distance #2
mean3	1.899	(1.762,2.037)	nm	Average inter-spin distance #3
mean4	2.006	(1.998,2.014)	nm	Average inter-spin distance #4
mean5	3.735	(3.714,3.755)	nm	Average inter-spin distance #5
mean6	3.347	(3.280,3.414)	nm	Average inter-spin distance #6
chol11	0.332	(0.100,0.692)	nm	Cholesky factor ℓ11
chol22	0.107	(0.100,0.800)	nm	Cholesky factor ℓ22
chol33	0.465	(0.305,0.625)	nm	Cholesky factor ℓ33
chol44	0.427	(0.417,0.437)	nm	Cholesky factor ℓ44
chol55	0.106	(0.100,0.125)	nm	Cholesky factor ℓ55
chol66	0.219	(0.176,0.262)	nm	Cholesky factor ℓ66
tau1	0.412	(0.411,0.413)	μs	First inter-pulse delay
tau2	8.000	(frozen)	μs	Second inter-pulse delay
lamu_1	1.019	(0.000,10.000)	None	Amplitude of unmodulated pairwise pathway
lam1_1	0.001	(0.000,0.140)	None	Amplitude of pairwise pathway #1
lam23	0.000	(frozen)	None	Amplitude of pairwise pathways #2 and #3
decay	0.360	(0.307,0.413)	μs^{-1}	Decay rate
d	1.295	(1.255,1.336)	μM	Stretch factor
lamu_2	1.000	(0.000,10.000)	None	Amplitude of unmodulated pairwise pathway
lam1_2	0.001	(0.000,0.118)	None	Amplitude of pairwise pathway #1
lamu_3	0.994	(0.000,10.000)	None	Amplitude of unmodulated pairwise pathway
lam1_3	0.001	(0.000,0.102)	None	Amplitude of pairwise pathway #1
lamu_4	0.983	(0.000,10.000)	None	Amplitude of unmodulated pairwise pathway
lam1_4	0.001	(0.000,0.094)	None	Amplitude of pairwise pathway #1
scale_1	1.089	(1.088,1.090)	None	Echo amplitude
scale_2	1.146	(1.145,1.147)	None	Echo amplitude
scale_3	1.176	(1.175,1.177)	None	Echo amplitude
scale_4	1.157	(1.154,1.159)	None	Echo amplitude

Table S7: DeerLab's results summary of the analysis of the T111 triradical oligoPPE TRIER data. The first table collects the estimated noise level (standard deviation), reduced chi-square, RMSD and AIC values of the individual dataset fits. The second table shows the DeerLab parameter name, fitted value, 95%-confidence intervals, unit, and description for the individual model parameters fitted during the analysis.

Goodness-of-fit:

Dataset	Noise level	Reduced χ2	RMSD	AIC
#1	0.003	8.329	0.009	-3386.798

Model parameters:

Parameter	Value	95%-Confidence interval	Unit	Description
mean1	3.570	(2.910,4.230)	nm	Average inter-spin distance #1
mean2	3.610	(2.853,4.366)	nm	Average inter-spin distance #2
mean3	3.623	(2.904,4.342)	nm	Average inter-spin distance #3
chol11	0.463	(0.462,0.463)	nm	Cholesky factor ℓ11
chol22	0.459	(0.459,0.459)	nm	Cholesky factor ℓ22
chol33	0.459	(0.459,0.459)	nm	Cholesky factor ℓ33
chol21	0.001	(0.001,0.002)	nm	Cholesky factor ℓ21
chol31	-0.001	(-0.001,-0.000)	nm	Cholesky factor ℓ31
chol32	-0.001	(-0.001,-0.001)	nm	Cholesky factor ℓ32
tau1	0.428	(0.425,0.430)	μs	First inter-pulse delay
tau2	4.557	(4.400,4.800)	μs	Second inter-pulse delay
tau3	0.911	(0.909,0.914)	μs	Third inter-pulse delay
lamu	0.981	(0.216,1.746)	None	Amplitude of unmodulated pairwise pathway
lam1	0.034	(0.000,0.085)	None	Dipolar pathway #1 amplitude
lam2	0.048	(0.000,0.116)	None	Dipolar pathway #2 amplitude
lam3	0.001	(0.000,0.006)	None	Dipolar pathway #3 amplitude
lam4	0.000	(0.000,0.002)	None	Dipolar pathway #4 amplitude
lam5	0.000	(frozen)	None	Dipolar pathway #5 amplitude
lam6	0.000	(frozen)	None	Dipolar pathway #6 amplitude
lam7	0.000	(frozen)	None	Dipolar pathway #7 amplitude
lam8	0.000	(frozen)	None	Dipolar pathway #8 amplitude
lam9	0.000	(0.000,0.002)	None	Dipolar pathway #9 amplitude
lam10	0.001	(0.000,0.014)	None	Dipolar pathway #10 amplitude
lam11	0.001	(0.000,0.028)	None	Dipolar pathway #11 amplitude
lam12	0.000	(0.000,0.001)	None	Dipolar pathway #12 amplitude
lam13	0.000	(frozen)	None	Dipolar pathway #13 amplitude
lam14	0.000	(frozen)	None	Dipolar pathway #14 amplitude
lam15	0.002	(0.000,0.023)	None	Dipolar pathway #15 amplitude
lam16	0.000	(0.000,0.001)	None	Dipolar pathway #16 amplitude
lam17	0.000	(frozen)	None	Dipolar pathway #17 amplitude
lam18	0.000	(frozen)	None	Dipolar pathway #18 amplitude
lam19	0.000	(frozen)	None	Dipolar pathway #19 amplitude
lam20	0.000	(frozen)	None	Dipolar pathway #20 amplitude
lam21	0.000	(frozen)	None	Dipolar pathway #21 amplitude
lam22	0.000	(frozen)	None	Dipolar pathway #22 amplitude
lam23	0.000	(frozen)	None	Dipolar pathway #23 amplitude
lam24	0.000	(frozen)	None	Dipolar pathway #24 amplitude
decay	0.139	(0.050,0.228)	μs^{-1}	Decay rate
d	1.110	(1.000,1.344)	μM	Stretch factor
scale	1.000	(1.000,1.000)	None	Echo amplitude

Table S8: DeerLab's results summary of the analysis of the T111 triradical oligoPPE TRIER data. The first table collects the estimated noise level (standard deviation), reduced chi-square, RMSD and AIC values of the individual dataset fits. The second table shows the DeerLab parameter name, fitted value, 95%-confidence intervals, unit, and description for the individual model parameters fitted during the analysis.

Goodness-of-fit:

Dataset	Noise level	Reduced χ2	RMSD	AIC
#1	0.004	3.420	0.007	-3604.884

Model parameters:

Parameter	Value	95%-Confidence interval	Unit	Description
mean1	3.178	(2.436,3.921)	nm	Average inter-spin distance #1
mean2	3.170	(2.131,4.209)	nm	Average inter-spin distance #2
mean3	3.167	(1.997,4.338)	nm	Average inter-spin distance #3
chol11	0.465	(0.464,0.467)	nm	Cholesky factor ℓ11
chol22	0.435	(0.434,0.437)	nm	Cholesky factor ℓ22
chol33	0.441	(0.440,0.442)	nm	Cholesky factor ℓ33
chol21	0.027	(0.027,0.028)	nm	Cholesky factor ℓ21
chol31	-0.024	(-0.025,-0.024)	nm	Cholesky factor ℓ31
chol32	0.050	(-0.399,0.498)	nm	Cholesky factor ℓ32
tau1	0.387	(0.384,0.391)	μs	First inter-pulse delay
tau2	2.778	(2.701,2.856)	μs	Second inter-pulse delay
tau3	0.947	(0.945,0.949)	μs	Third inter-pulse delay
lamu	0.885	(0.416,1.354)	None	Amplitude of unmodulated pairwise pathway
lam1	0.033	(0.002,0.064)	None	Dipolar pathway #1 amplitude
lam2	0.035	(0.003,0.067)	None	Dipolar pathway #2 amplitude
lam3	0.001	(0.000,0.027)	None	Dipolar pathway #3 amplitude
lam4	0.000	(0.000,0.004)	None	Dipolar pathway #4 amplitude
lam5	0.000	(frozen)	None	Dipolar pathway #5 amplitude
lam6	0.000	(frozen)	None	Dipolar pathway #6 amplitude
lam7	0.000	(frozen)	None	Dipolar pathway #7 amplitude
lam8	0.000	(frozen)	None	Dipolar pathway #8 amplitude
lam9	0.000	(0.000,0.002)	None	Dipolar pathway #9 amplitude
lam10	0.006	(0.000,0.027)	None	Dipolar pathway #10 amplitude
lam11	0.002	(0.000,0.180)	None	Dipolar pathway #11 amplitude
lam12	0.000	(0.000,0.001)	None	Dipolar pathway #12 amplitude
lam13	0.000	(frozen)	None	Dipolar pathway #13 amplitude
lam14	0.000	(frozen)	None	Dipolar pathway #14 amplitude
lam15	0.003	(0.000,0.156)	None	Dipolar pathway #15 amplitude
lam16	0.001	(0.000,0.001)	None	Dipolar pathway #16 amplitude
lam17	0.000	(frozen)	None	Dipolar pathway #17 amplitude
lam18	0.000	(frozen)	None	Dipolar pathway #18 amplitude
lam19	0.000	(frozen)	None	Dipolar pathway #19 amplitude
lam20	0.000	(frozen)	None	Dipolar pathway #20 amplitude
lam21	0.000	(frozen)	None	Dipolar pathway #21 amplitude
lam22	0.000	(frozen)	None	Dipolar pathway #22 amplitude
lam23	0.000	(frozen)	None	Dipolar pathway #23 amplitude
lam24	0.000	(frozen)	None	Dipolar pathway #24 amplitude
decay	0.593	(0.077,1.109)	μs^{-1}	Decay rate
d	1.112	(1.000,1.322)	μM	Stretch factor
scale	1.049	(1.049,1.049)	None	Echo amplitude

Table S9: DeerLab's results summary of the analysis of the Rpo47 triradical protein complex TRIER data. The first table collects the estimated noise level (standard deviation), reduced chi-square, RMSD and AIC values of the individual dataset fits. The second table shows the DeerLab parameter name, fitted value, 95%-confidence intervals, unit, and description for the individual model parameters fitted during the analysis.

Goodness-of-fit:

Dataset	Noise level	Reduced χ2	RMSD	AIC
#1	0.009	1.703	0.012	-2678.968

Model parameters:

Parameter	Value	95%-Confidence interval	Unit	Description
mean1	2.839	(2.767,2.911)	nm	Average inter-spin distance #1
mean2	4.551	(4.441,4.660)	nm	Average inter-spin distance #2
mean3	6.323	(5.678,6.969)	nm	Average inter-spin distance #3
chol11	0.398	(0.323,0.474)	nm	Cholesky factor ℓ11
chol22	0.414	(0.412,0.415)	nm	Cholesky factor ℓ22
chol33	0.325	(0.242,0.408)	nm	Cholesky factor ℓ33
chol21	-0.103	(-0.105,-0.101)	nm	Cholesky factor ℓ21
chol31	0.160	(0.077,0.243)	nm	Cholesky factor ℓ31
chol32	0.080	(0.004,0.155)	nm	Cholesky factor ℓ32
tau1	0.388	(0.380,0.395)	μs	First inter-pulse delay
tau2	5.406	(5.200,5.600)	μs	Second inter-pulse delay
tau3	0.431	(0.426,0.436)	μs	Third inter-pulse delay
lamu	0.824	(0.392,1.257)	None	Amplitude of unmodulated pairwise pathway
lam1	0.035	(0.000,0.070)	None	Dipolar pathway #1 amplitude
lam2	0.055	(0.005,0.105)	None	Dipolar pathway #2 amplitude
lam3	0.004	(0.000,1.000)	None	Dipolar pathway #3 amplitude
lam4	0.000	(0.000,1.000)	None	Dipolar pathway #4 amplitude
lam5	0.000	(frozen)	None	Dipolar pathway #5 amplitude
lam6	0.000	(frozen)	None	Dipolar pathway #6 amplitude
lam7	0.000	(frozen)	None	Dipolar pathway #7 amplitude
lam8	0.000	(frozen)	None	Dipolar pathway #8 amplitude
lam9	0.000	(0.000,0.079)	None	Dipolar pathway #9 amplitude
lam10	0.014	(0.000,0.585)	None	Dipolar pathway #10 amplitude
lam11	0.000	(0.000,0.177)	None	Dipolar pathway #11 amplitude
lam12	0.000	(0.000,0.129)	None	Dipolar pathway #12 amplitude
lam13	0.000	(frozen)	None	Dipolar pathway #13 amplitude
lam14	0.000	(frozen)	None	Dipolar pathway #14 amplitude
lam15	0.004	(0.000,1.000)	None	Dipolar pathway #15 amplitude
lam16	0.000	(0.000,1.000)	None	Dipolar pathway #16 amplitude
lam17	0.000	(frozen)	None	Dipolar pathway #17 amplitude
lam18	0.000	(frozen)	None	Dipolar pathway #18 amplitude
lam19	0.000	(frozen)	None	Dipolar pathway #19 amplitude
lam20	0.000	(frozen)	None	Dipolar pathway #20 amplitude
lam21	0.000	(frozen)	None	Dipolar pathway #21 amplitude
lam22	0.000	(frozen)	None	Dipolar pathway #22 amplitude
lam23	0.000	(frozen)	None	Dipolar pathway #23 amplitude
lam24	0.000	(frozen)	None	Dipolar pathway #24 amplitude
conc	40.665	(0.000,174.032)	μM	Spin concentration
d	1.000	(frozen)	μM	Stretch factor
scale	1.022	(1.022,1.022)	None	Echo amplitude

V. Two-spin model analyses

Analysis of the 4-pulse DEER datasets using two-spin models with non-parametric distance distributions with DeerLab. Figures S4-S7 show the results of a global analysis over all database with a global distance distribution. Figures S8-S11 show the results of the local analyses of the individual datasets.

The results show the effects of the incorrect modelling of multi-spin systems as two-spin systems.

Analysis script: `analysis_twospin_DEER.ipynb`

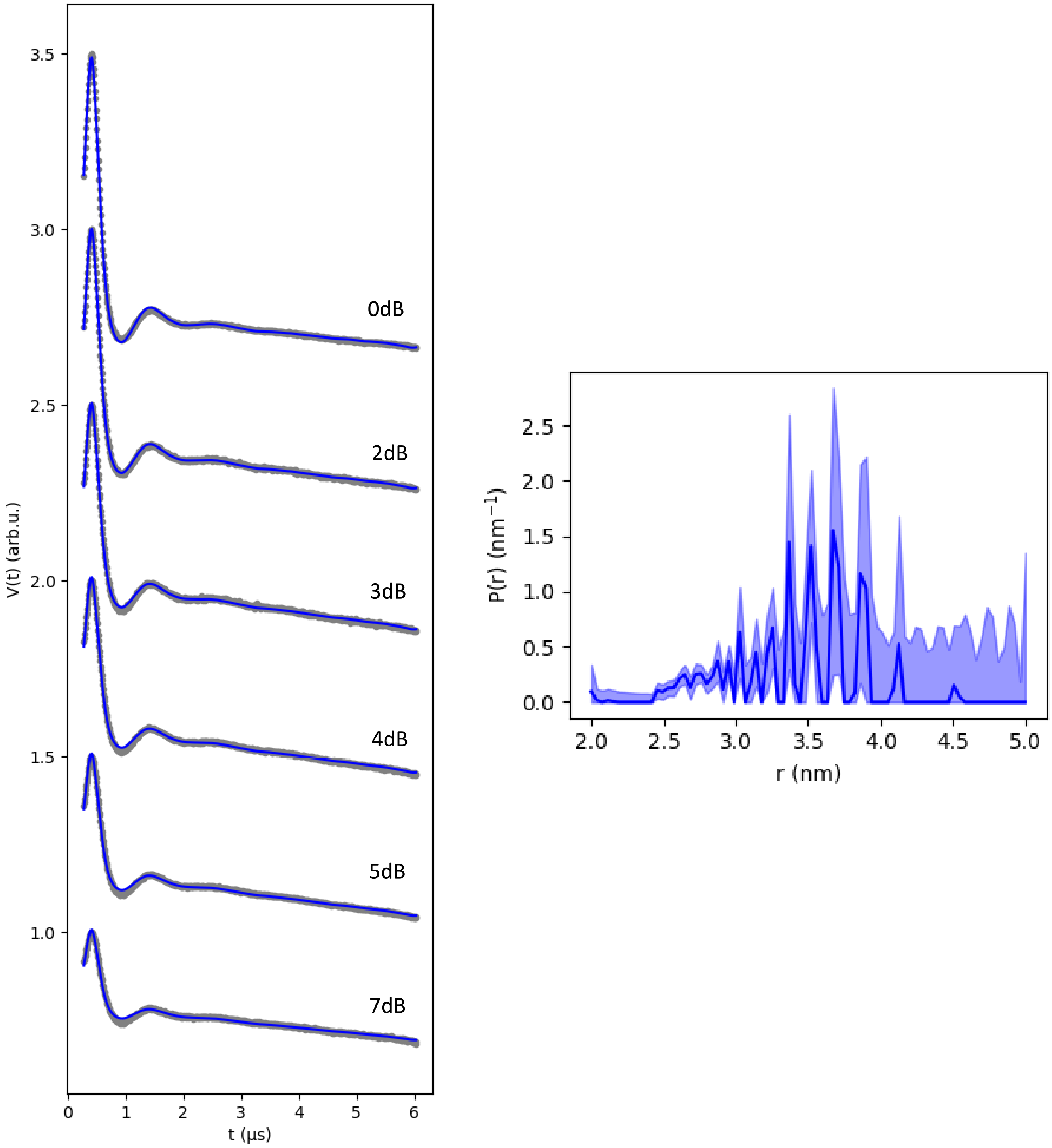

Figure S4: Global analysis with DeerLab of the T111 triradical oligoPPE 4-pulse DEER data acquired at different power attenuation levels (indicated next to each dataset) using a two-spin model with a non-parametric distance distribution. The left panel shows the experimental data as grey dots and the fitted model as a blue line. The right panel shows the global non-parametric distance distribution as a blue line with its 95%-confidence intervals shown as a blue shaded area.

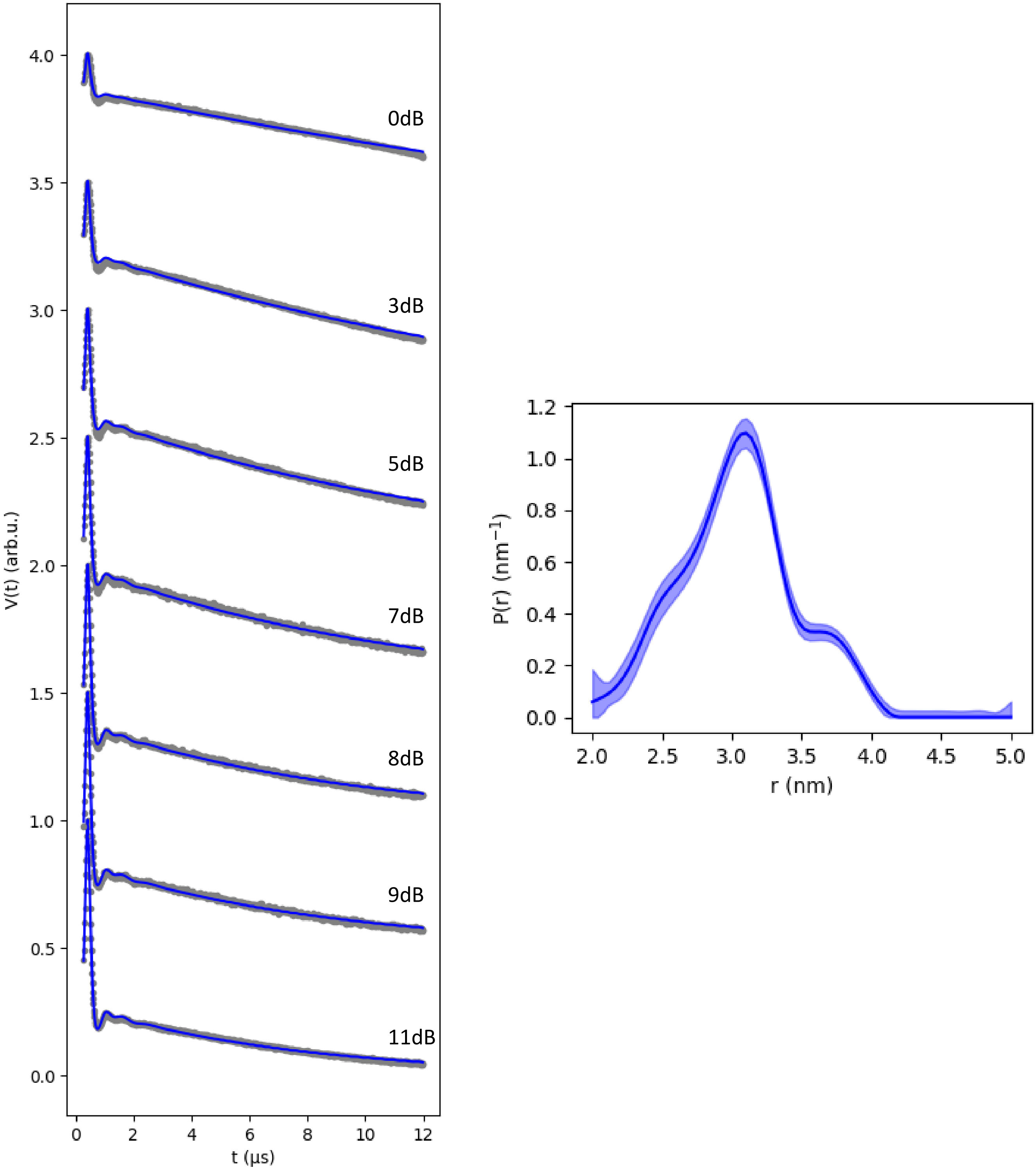

Figure S5: Global analysis with DeerLab of the T011 triradical oligoPPE 4-pulse DEER data acquired at different power attenuation levels (indicated next to each dataset) using a two-spin model with a non-parametric distance distribution. The left panel shows the experimental data as grey dots and the fitted model as a blue line. The right panel shows the global non-parametric distance distribution as a blue line with its 95%-confidence intervals shown as a blue shaded area.

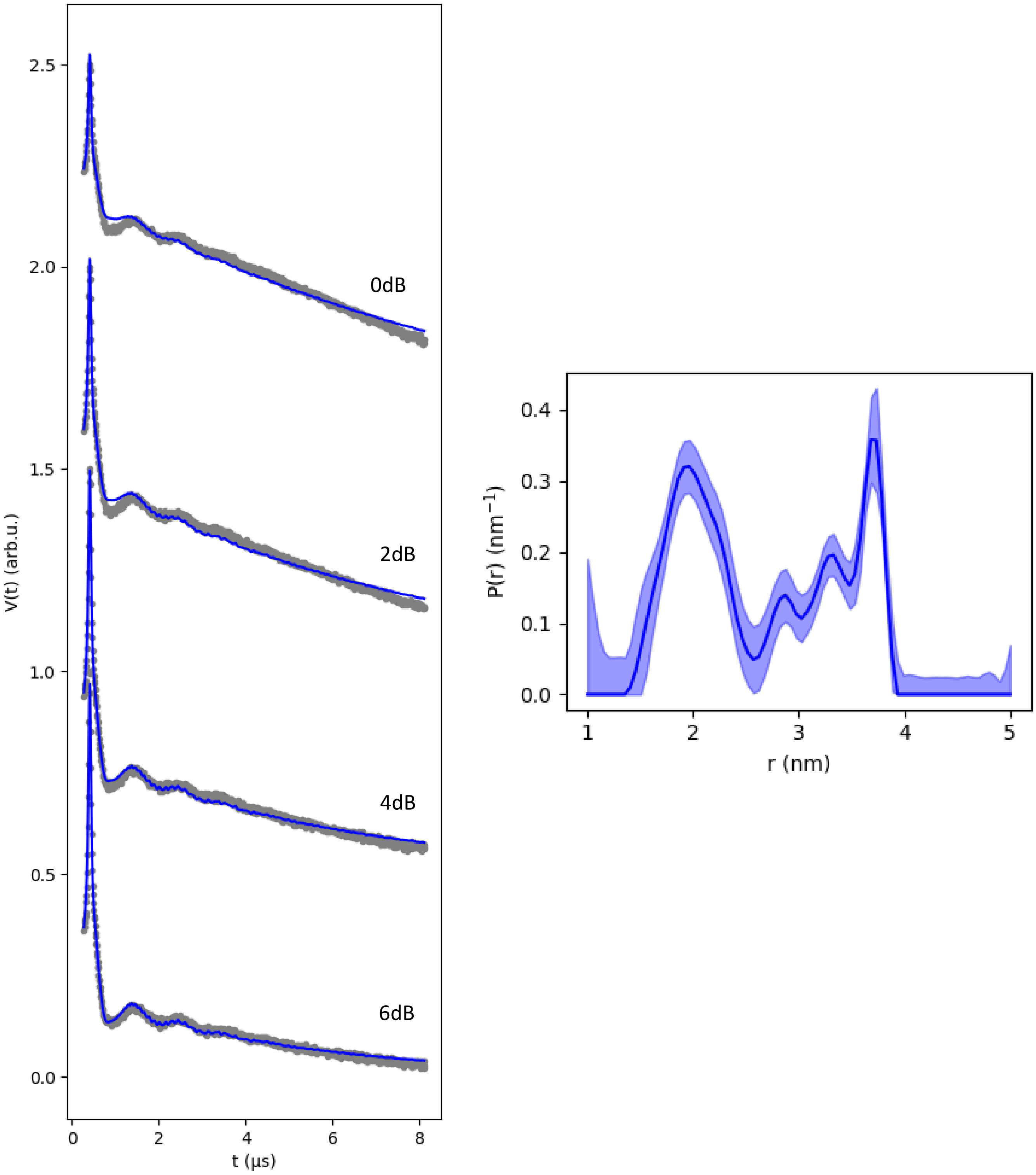

Figure S6: Global analysis with DeerLab of the tetraradical oligoPPE 4-pulse DEER data acquired at different power attenuation levels (indicated next to each dataset) using a two-spin model with a non-parametric distance distribution. The left panel shows the experimental data as grey dots and the fitted model as a blue line. The right panel shows the global non-parametric distance distribution as a blue line with its 95%-confidence intervals shown as a blue shaded area.

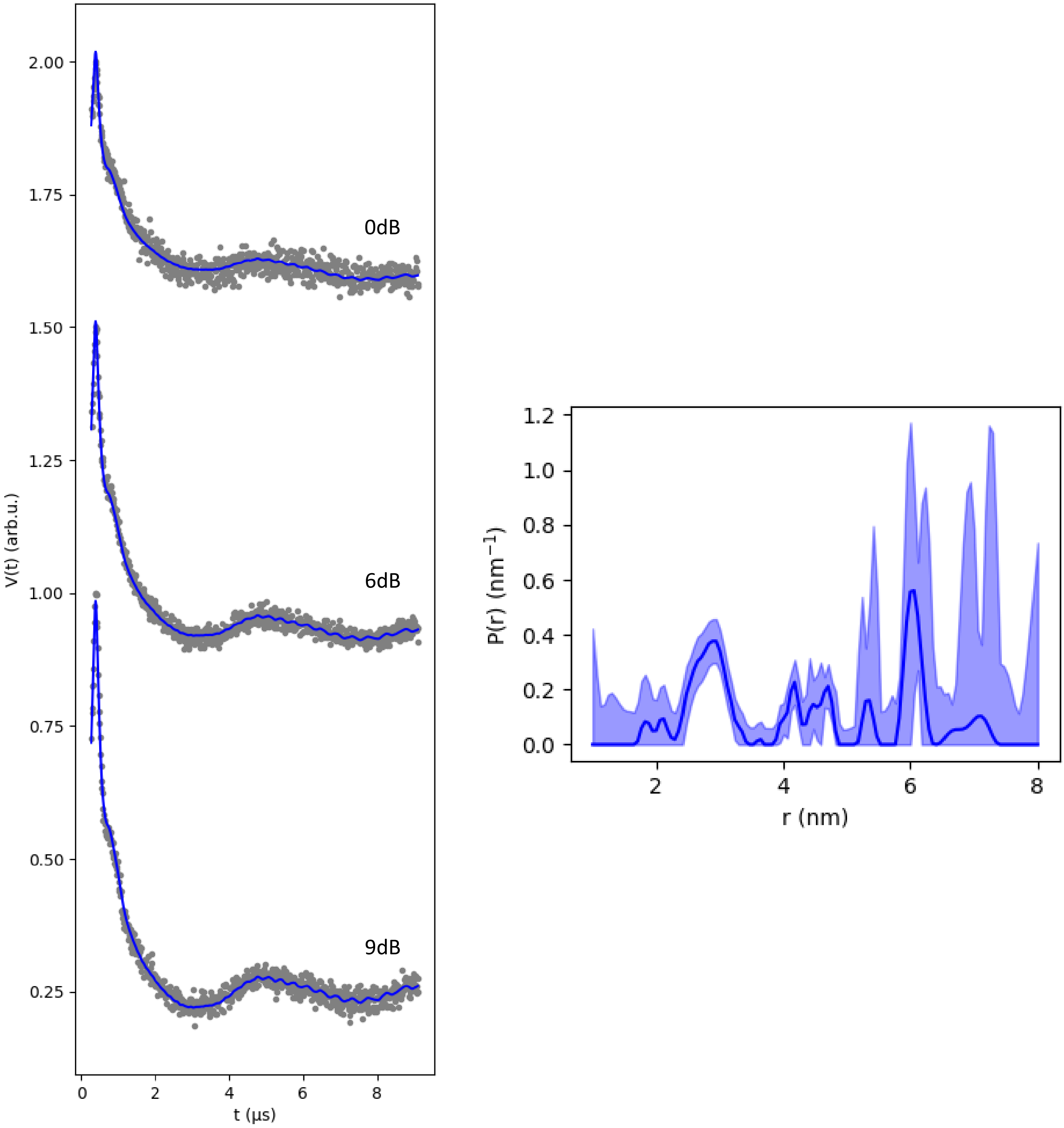

Figure S7: Global analysis with DeerLab of the Rpo47 triradical protein complex 4-pulse DEER data acquired at different power attenuation levels (indicated next to each dataset) using a two-spin model with a non-parametric distance distribution. The left panel shows the experimental data as grey dots and the fitted model as a blue line. The right panel shows the global non-parametric distance distribution as a blue line with its 95%-confidence intervals shown as a blue shaded area.

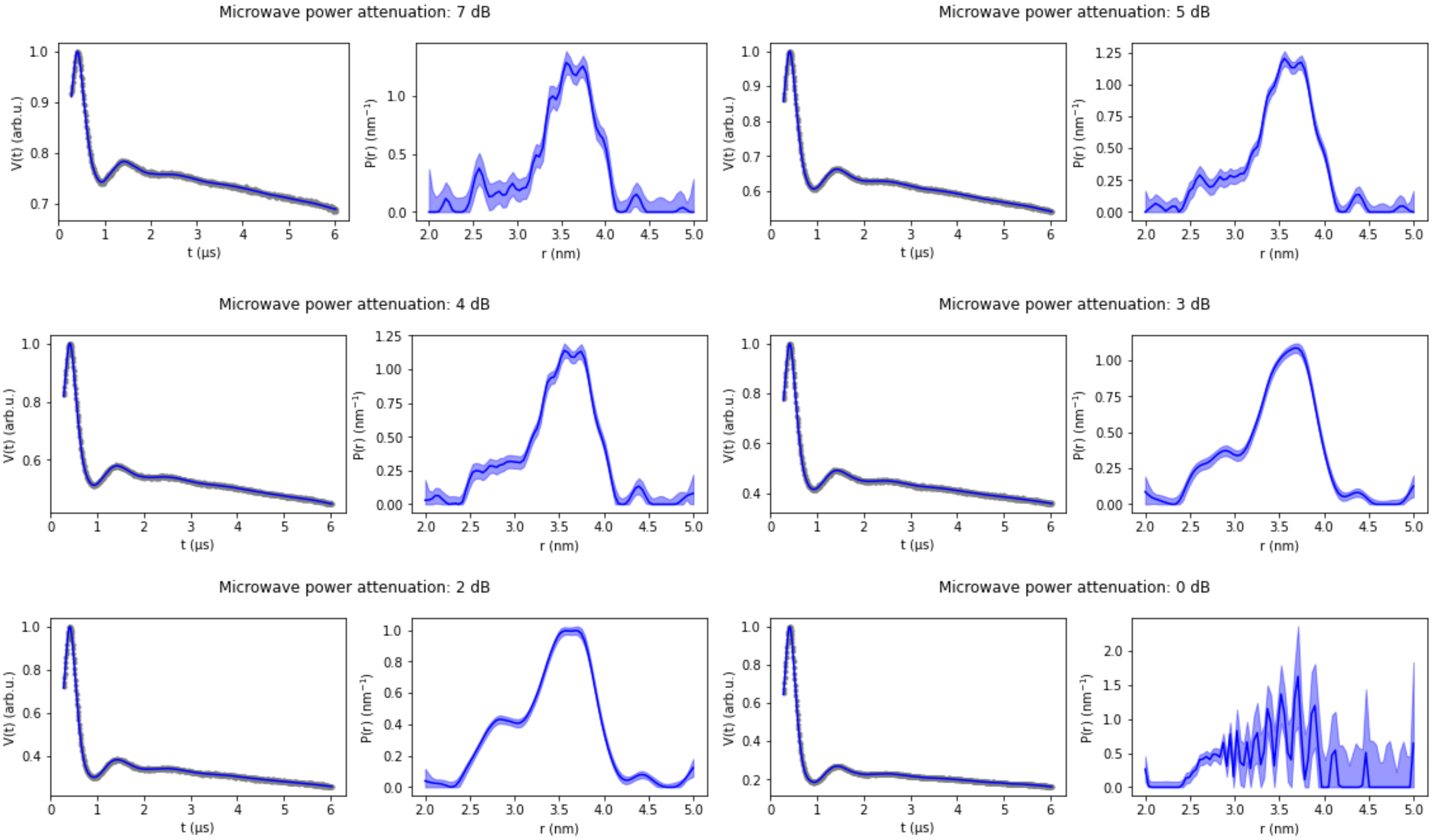

Figure S8: Local analysis with DeerLab of the T111 triradical oligoPPE 4-pulse DEER data using a two-spin model with a non-parametric distance distribution. For each dataset, the left panels show the experimental data as grey dots and the fitted model as a blue line. The right panels show the corresponding non-parametric distance distributions as a blue line with their 95%-confidence intervals shown as a blue shaded area.

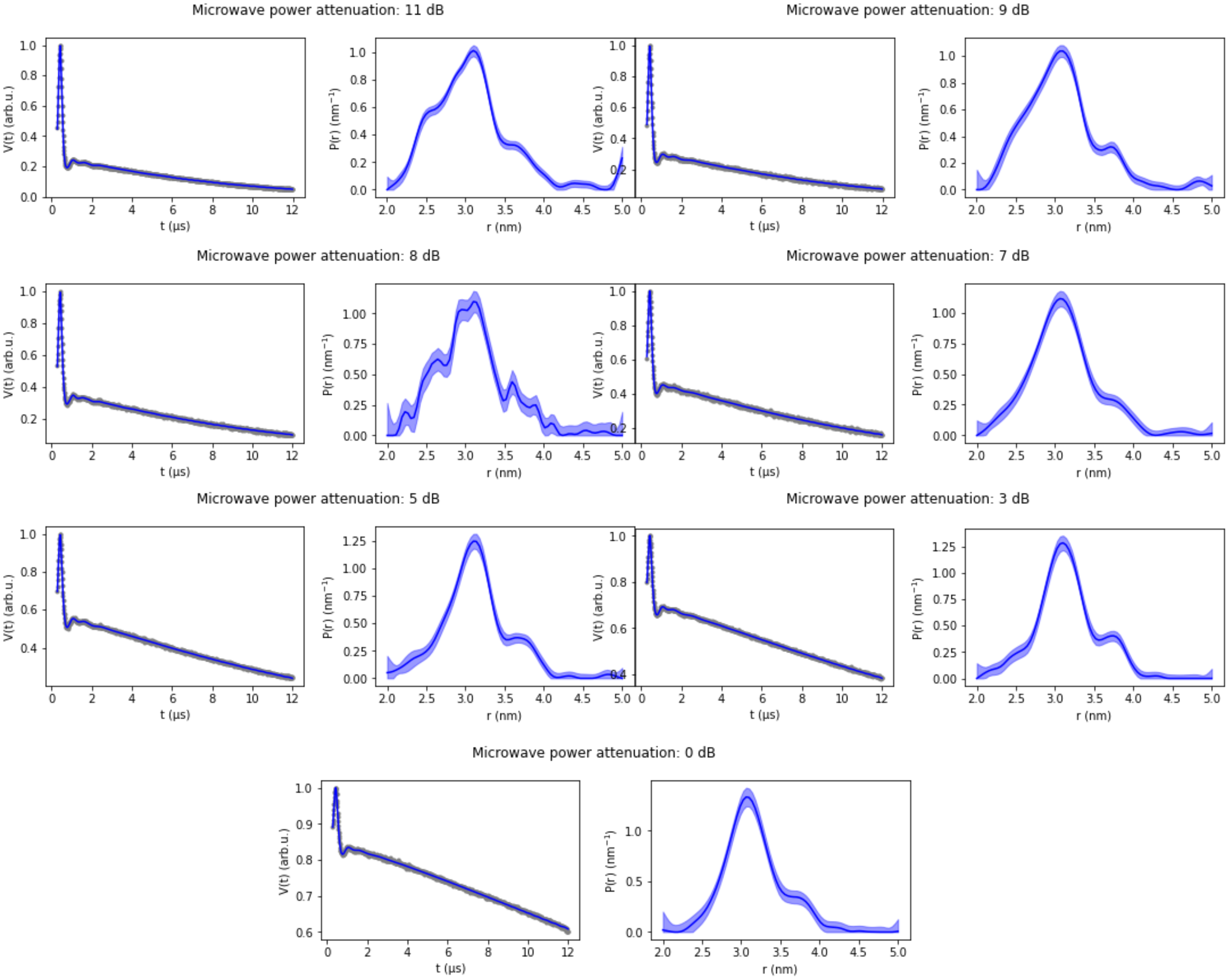

Figure S9: Local analysis with DeerLab of the T011 triradical oligoPPE 4-pulse DEER data using a two-spin model with a non-parametric distance distribution. For each dataset, the left panels show the experimental data as grey dots and the fitted model as a blue line. The right panels show the corresponding non-parametric distance distributions as a blue line with their 95%-confidence intervals shown as a blue shaded area.

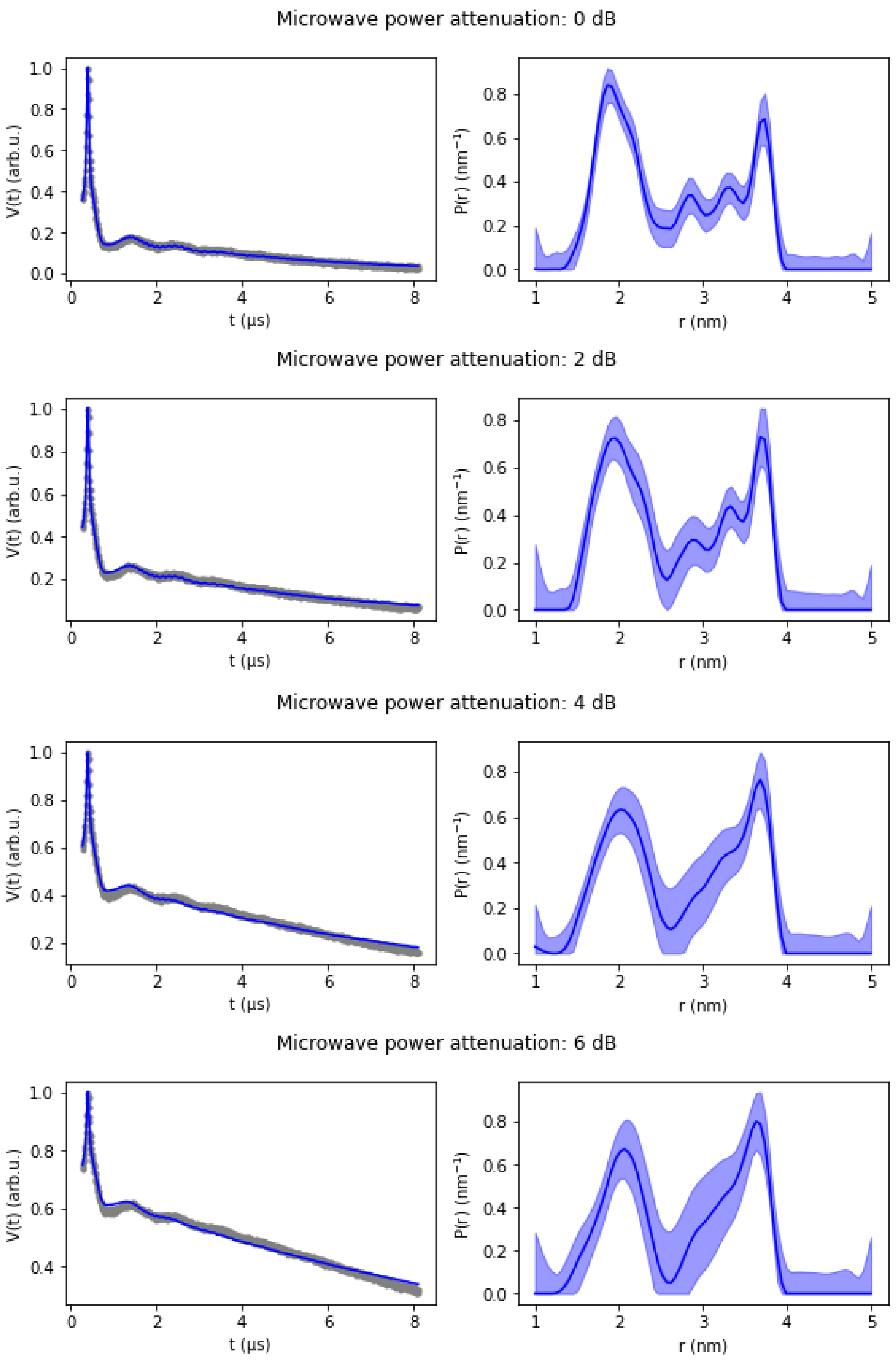

Figure S10: Local analysis with DeerLab of the tetraradical oligoPPE 4-pulse DEER data using a two-spin model with a non-parametric distance distribution. For each dataset, the left panels show the experimental data as grey dots and the fitted model as a blue line. The right panels show the corresponding non-parametric distance distributions as a blue line with their 95%-confidence intervals shown as a blue shaded area.

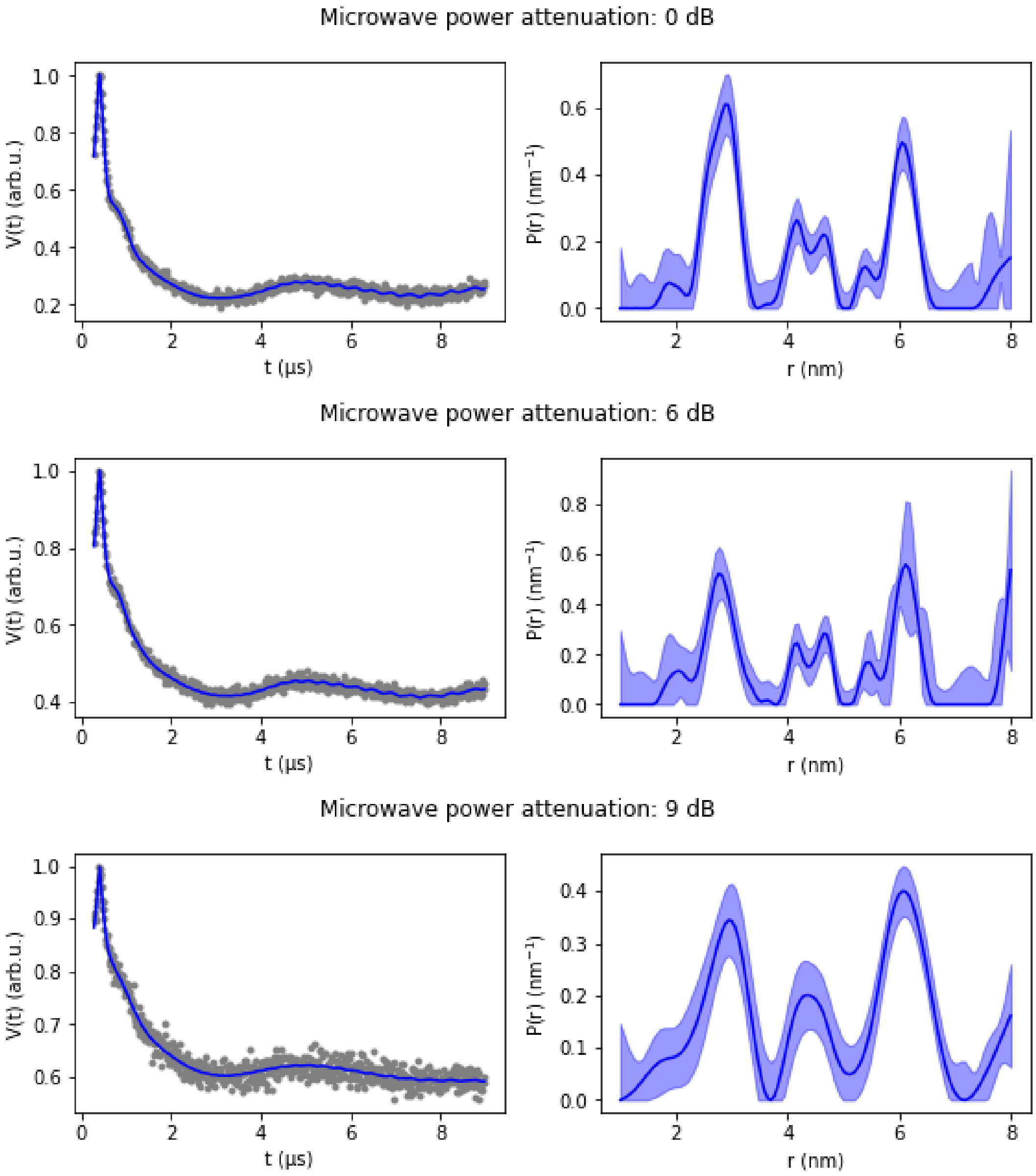

Figure S11: Local analysis with DeerLab of the Rpo47 protein complex 4-pulse DEER data using a two-spin model with a non-parametric distance distribution. For each dataset, the left panels show the experimental data as grey dots and the fitted model as a blue line. The right panels show the corresponding non-parametric distance distributions as a blue line with their 95%-confidence intervals shown as a blue shaded area.
